# Understanding Providers’ Attitude Toward AI in India’s Informal Health Care Sector: Survey Study

**DOI:** 10.2196/54156

**Published:** 2025-02-10

**Authors:** Sumeet Kumar, Snehil Rayal, Raghuram Bommaraju, Navya Pratyusha Varasala, Sirisha Papineni, Sarang Deo

**Affiliations:** 1 Indian School of Business, Gachibowli, ISB Road, Hyderabad, India, +91 7075969318

**Keywords:** artificial intelligence, tuberculosis, health care providers, cross-sectional studies, trust, x-rays, India

## Abstract

**Background:**

Tuberculosis (TB) is a major global health concern, causing 1.5 million deaths in 2020. Diagnostic tests for TB are often inaccurate, expensive, and inaccessible, making chest x-rays augmented with artificial intelligence (AI) a promising solution. However, whether providers are willing to adopt AI is not apparent.

**Objective:**

The study seeks to understand the attitude of Ayurveda, Yoga and Naturopathy, Unani, Siddha, and Homoeopathy (AYUSH) and informal health care providers, who we jointly call AIPs, toward adopting AI for TB diagnosis. We chose to study these providers as they are the first point of contact for a majority of TB patients in India.

**Methods:**

We conducted a cross-sectional survey of 406 AIPs across the states of Jharkhand (162 participants) and Gujarat (244 participants) in India. We designed the survey questionnaire to assess the AIPs’ confidence in treating presumptive TB patients, their trust in local radiologists’ reading of the chest x-ray images, their beliefs regarding the diagnostic capabilities of AI, and their willingness to adopt AI for TB diagnosis.

**Results:**

We found that 93.7% (270/288) of AIPs believed that AI could improve the accuracy of TB diagnosis, and for those who believed in AI, 71.9% (194/270) were willing to try AI. Among all AIPs, 69.4% (200/288) were willing to try AI. However, we found significant differences in AIPs’ willingness to try AI across the 2 states. Specifically, in Gujarat, a state with better and more accessible health care infrastructure, 73.4% (155/211) were willing to try AI, and in Jharkhand, 58.4% (45/77) were willing to try AI. Moreover, AIPs in Gujarat who showed higher trust in the local radiologists were less likely to try AI (odds ratio [OR] 0.15, 95% CI 0.03‐0.69; *P*=.02). In contrast, in Jharkhand, those who showed higher trust in the local radiologists were more likely to try AI (OR 2.11, 95% CI 0.9‐4.93; *P*=.09).

**Conclusions:**

While most AIPs believed in the potential benefits of AI-based TB diagnoses, many did not intend to try AI, indicating that the expected benefits of AI measured in terms of technological superiority may not directly translate to impact on the ground. Improving beliefs among AIPs with poor access to radiology services or those who are less confident of diagnosing TB is likely to result in a greater impact of AI on the ground. Additionally, tailored interventions addressing regional and infrastructural differences may facilitate AI adoption in India’s informal health care sector.

## Introduction

Tuberculosis (TB) remains a significant global health challenge, with over 80% of reported cases and deaths originating from low- and middle-income countries (LMICs) worldwide [1]. Among these countries, India shoulders a substantial burden, accounting for a quarter of all TB cases and resulting in approximately 89,000 deaths in the year 2019 alone [2]. The COVID-19 pandemic further worsened these global inequalities, particularly by disrupting TB diagnostic and treatment services [3,4]. Data from the World Health Organization (WHO) reveals a concerning trend in the incidence rate of TB. After experiencing a decline of around 2% per year over the past 2 decades, there has been a 3.6% increase in new TB cases per 100,000 population between 2020 and 2021, as indicated in the Global Tuberculosis Report [5]. Additionally, due to factors like underreporting, underdiagnosis, and limited access to health care for TB patients, a significant gap exists between the estimated and reported number of cases [6]. In India, 29% of TB cases were either undiagnosed or missed in 2021 [7,8]. This resurgence of TB cases [9] is particularly alarming for LMICs like India, which bear a disproportionate disease burden [10] and face numerous challenges [6] in their fragile health care supply chains.

To lessen this burden, recent advancements in diagnosis based on molecular testing and chest x-ray (CXR) scans offer promising avenues. However, the highly accurate molecular diagnostic tests for TB, such as Xpert MTB/RIF (*Mycobacterium tuberculosis*/rifampin), remain expensive, difficult to access, and challenging to maintain in LMICs [11]. Additionally, their roll-out has been slow [12]. Although WHO recommends molecular diagnostics as the preferred first-line testing method, only 38% of individuals diagnosed with TB in 2021 were tested with a WHO-recommended rapid molecular diagnostic at the initial diagnosis [12]. The other option of clinical diagnosis based on CXRs continues to be the mainstay among health care providers in India [13]. CXR is recommended by WHO for screening, triaging, and assisting in the diagnosis of TB. It is also featured in the national TB diagnostic guidelines of 19 out of 22 countries with the highest TB burden [14]. However, there is a shortage of adequately trained radiologists proficient in analyzing CXRs and producing high-quality reports [15]. This shortage has important implications, as the majority of TB care providers are not formally trained to interpret CXRs [16].

In the absence of qualified radiologists, the presence of automated artificial intelligence (AI) systems for interpreting CXRs could prove to be highly advantageous [17,18]. Employing AI-assisted interpretation of CXRs can improve the speed [19] and enhance the accuracy of TB diagnosis [20]. Addressing the delays in TB diagnosis through earlier TB detection is a key element of the WHO’s TB control and elimination strategy [21]. Moreover, the more recent deep learning–based AI systems for CXR interpretation have demonstrated on par or higher specificity than radiologists [22-24], and their usage can significantly reduce the cost of TB screening [25]. These systems are also portable and can additionally be accessed remotely via the internet, making them a good choice for providers who often serve in remote locations.

However, the successful adoption of AI for the accurate diagnosis of TB depends on many factors [26]. In particular, adoption by the private sector is needed, as it plays a significant role in treating TB cases in India, diagnosing twice as many cases as compared to the public sector, amounting to an estimated 2.2 million TB cases [27]. The private sector is fragmented, consisting of a variety of health care providers, clinics, and practitioners that lack integration, especially in small towns and villages [28]. A study conducted [29] revealed that the average Indian village has 3.2 primary health care providers, with 68% lacking formal medical training and referred to as informal health care providers (IPs). In addition, India has Ayurveda, Yoga and Naturopathy, Unani, Siddha, and Homoeopathy (AYUSH) providers formally trained in one of the 6 traditional Indian systems of medicine, constituting 22.8% of formally trained medical practitioners in India [30]. From this point forward, we will use the abbreviation “AIPs” to collectively denote both informal health care providers and AYUSH practitioners. For faster and more accurate TB diagnosis, AIPs need to adopt AI systems. However, the adoption will only happen if AIPs perceive value in AI, which has not been examined in prior research.

In this paper, we conducted a survey study to understand the attitudes and perspectives of AIPs toward adopting AI for TB diagnosis. AIPs are the first point of contact in patient pathways for the majority of TB cases in India, so their willingness to incorporate AI-enhanced CXR systems into the decision-making is a key factor in driving AI adoption. Adopting AI could enhance AIPs’ capability to interpret CXR scans and detect TB cases earlier and more accurately. In order to assess AIPs’ beliefs in the diagnostic capabilities of AI and their willingness to adopt AI for TB diagnosis, we conducted a cross-sectional survey of 406 AIPs across the states of Jharkhand (162 participants) and Gujarat (244 participants).

## Methods

### Overview

We conducted a cross-sectional survey of AIPs in 2 Indian states (Gujarat and Jharkhand) to understand their current practices and potential barriers in adopting AI in their practice. The guidelines in the Strengthening the Reporting of Observational Studies in Epidemiology (STROBE) were followed (Checklist 1).

Jharkhand is one of the poorer states in India with lower technology penetration and lower access to radiologic facilities as well as radiologists, and overall a less developed private sector. In contrast, Gujarat is a more developed state with an active and accessible private health care infrastructure.

### Participants Recruitment and Enrollment

We collaborated with World Health Partner (WHP), a non-profit organization that has partnered with x-ray labs and AIPs in Gujarat and Jharkhand. In Gujarat, WHP worked with 73 x-ray labs and 757 AIPs, while in Jharkhand, they collaborated with 113 x-ray labs and 406 AIPs.

Multiple methods were used to identify the AIPs. Field investigators from WHP mapped the AIPs in the districts of Ranchi and East Singhbhum in Jharkhand, as well as Surat and Gandhinagar in Gujarat. Investigators queried TB patient cohorts (those in their first year of treatment) about the health care providers they visited during their treatment. Allopathic providers were interviewed to identify nearby AIPs who frequently referred cases to them. Additionally, government officials and partner organizations at the community level helped identify local health care provider networks. Radiology units offering CXR services were also consulted to locate AIPs in their vicinity. The investigators used the mobile Commcare application to map these providers, capturing details such as contact information, qualifications, outpatient department load, referral linkages, and more, which were then used to generate a unique ID for each provider (form included in Multimedia Appendix 1). Each AIP clinic functioned as an independent study or clinical setting.

For our study, we asked all identified AIPs working with WHP for their participation and enrolled 406 AIPs based on their willingness to participate in the survey. In return, we provided a mobile recharge of ₹300 (US $3.47) for completing the survey. These participants were from 18 blocks across 2 districts in Gujarat and 22 blocks spanning 2 districts in Jharkhand. The survey was conducted between February 2022 and September 2022.

### Data

We developed the initial questionnaire by leveraging questions from established scales and modifying them to suit our study’s context. This process included adapting sections on AI in TB diagnosis from existing literature [31,32] and customizing questions to assess AIPs’ comfort with technology and demographics [33]. Additionally, we adapted from the literature [34] the questions regarding AIPs’ trust in radiologists, ensuring they were pertinent to our specific research objectives.

The questionnaire underwent 7 iterative revisions in collaboration with our field partner, WHP, known for its extensive experience with TB providers and patients. Subsequently, Outline India, the data collection agency, conducted pretests that led to further refinements based on feedback from pilot studies. This phase involved translating the questionnaire into Hindi and Gujarati and making 8 additional revisions to improve clarity and relevance. Our research team oversaw the translation process to ensure its accuracy. The final, refined version of the questionnaire, which incorporated comprehensive feedback and amendments, received validation by the strategy lead at WHP.

We structured the questionnaire into multiple subsections to evaluate the following aspects: AIPs’ understanding of TB diagnosis; confidence in diagnosing TB patients; trust levels in local radiologists; beliefs and intentions regarding the adoption of AI in TB diagnosis; comfort levels with technology; and demographic details such as age, gender, state of residence, and years of experience. We conducted a survey of the registered AIPs by partnering with Outline India. This agency deployed field investigators proficient in the local languages (Hindi and Gujarati) and used specialized software for digital data collection.

We gathered data from 406 participating AIPs. The survey was conducted at the participants’ locations and lasted 10-20 minutes. Out of 406 AIPs, 39.9% (162/406) participants were from Jharkhand and 60.1% (244/406) participants were from Gujarat. In total, 85.2% (346/406) of the respondents were men, while women comprised 14.8% (60/406). The age of respondents ranged from 22 to 86 (mean 41.8, SD 11.4) years.

Responses to most of the questions were on a 5-point Likert scale as shown in Table 1. To illustrate the use of this scale, a score of “4” indicates that the participant somewhat agrees with the statement under consideration, whereas a score of “5” indicates strong agreement with the statement. For a few questions, respondents’ opinions were recorded as 1=Yes and 0=No using a dichotomous scale. The detailed questionnaire can be referenced in Multimedia Appendix 2.

**Table 1.** Likert Scale of 0‐5 capturing responses from “strongly disagree” to “strongly agree”.

Scale	Respondent’s opinion to question
5	Strongly agree
4	Somewhat agree
3	Neither agree nor disagree
2	Somewhat disagree
1	Strongly disagree

We encountered certain absent (not available) data points, stemming from incomplete surveys. These data points were eliminated before employing the data for regression, resulting in the removal of 92 observations. Out of these, 17 were from Gujarat, and 75 were from Jharkhand. The main reason for the reduction in the number of observations was the lack of responses for questions on “Trust in local radiologists” because many survey participants did not have access to radiologists.

**Figure 1.**
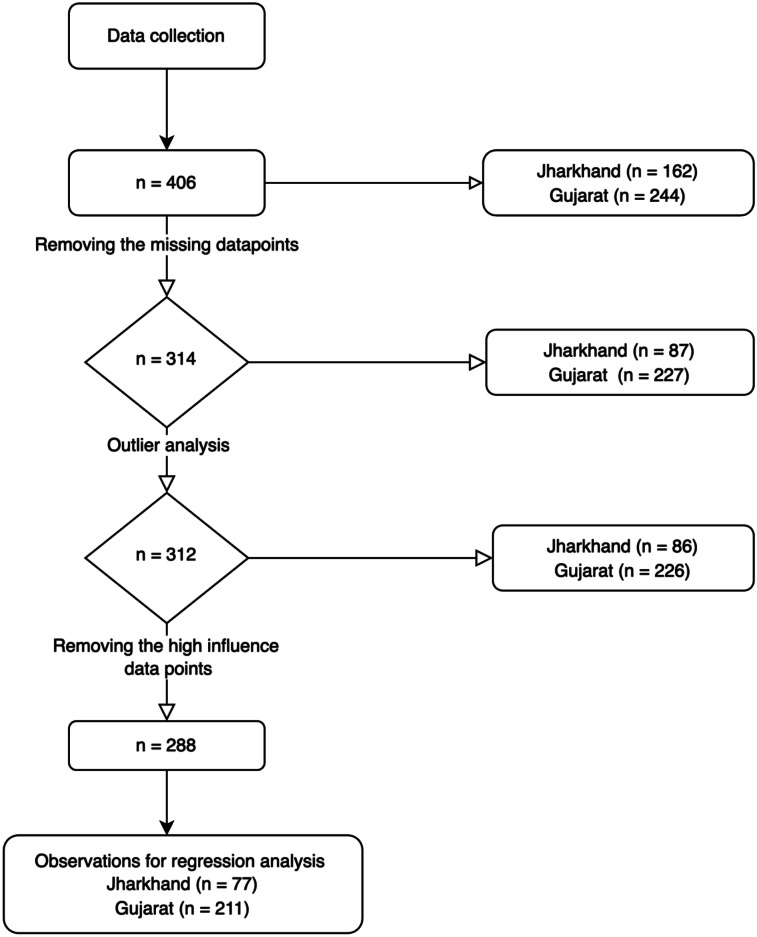
Data cleaning workflow reflecting multiple steps to arrive at the final dataset.

In addition, the outliers were identified and removed using boxplot and Cook distance analysis to eliminate any highly influential data points that could distort the results. This process resulted in a final data set consisting of 288 observations, with 211 observations from Gujarat and 77 observations from Jharkhand. We show the entire workflow for data cleaning in Figure 1. Multimedia Appendix 3 contains further information on outlier analysis and on descriptor variables for both the initial sample collected and the final sample used in the regression analysis.

### Variable Description and Analysis

In this study, we investigated two main constructs: (1) AI Intent and (2) AI Belief. Previous research on technology adoption has demonstrated that the actual utilization of a technology is preceded by factors such as “Intent to use,” “Attitude toward technology,” and considerations of “Ease of use” among others [35,36]. The ease of use of a product often evolves with the incorporation of new features and regular enhancements to the user experience, a common occurrence in the realm of internet technology products. Hence, we specifically focused on understanding individuals’ “Intent to use” AI products, which we refer to as “AI Intent”; and their “Attitude toward AI,” which we refer to as “AI Belief.”

We coded the response to the survey question, “Are you willing to try an AI software that could generate an x-ray report from an uploaded x-ray?” as a categorical variable “AI Intent” with 2 options: “Yes” or “No.” We used logistic regression to examine its association with the independent variables.

We created the “AI Belief” variable by combining the responses to 2 questions: “I believe AI algorithms can reliably detect imaging findings that suggest TB” and “I believe the use of AI could improve the accuracy of diagnosis of tuberculosis.” We calculated the mean of the responses to these questions to construct the “AI Belief” variable. It represents participants’ overall belief in the effectiveness of AI in improving TB diagnosis.

For independent variables in the regression analysis, we included “Confidence in diagnosing TB” and “Trust in local radiologists,” and demographic variables such as age, gender, and state of residence.

We coded “Confidence in diagnosing TB” as the mean of the responses to 5 questions from the survey questionnaire: “I feel confident in my ability to read an x-rays report to detect a TB case,” “I feel confident in my ability to interpret an x-rays film without an x-rays report to detect a TB case,” “I feel confident in my ability to deal with TB patients (if allowed to treat or medicate),” “I feel confident in my ability to use relevant diagnostic tests to detect a TB case,” and “I feel confident in my ability to collect relevant health-related information from patients.”

Similarly, we coded “Trust in local radiologists” as the mean of the responses to 3 questions: “The language and style of radiology reports are mostly clear,” “The radiologist who reads the x-rays and writes the report is competent,” and “I am generally satisfied with x-rays reports I receive from radiologists.” We calculated the Cronbach α using all the survey responses to assess the consistency of the scale. Gender and state of residence were treated as categorical.

We used Python (version 3.9.13) for data cleaning and data visualization, while data analysis was carried out using StataCorpSTATA SE (version 17.0) and Microsoft Excel.

### Ethical Considerations

The study protocols underwent review and approval by the Institutional Review Board at the Indian School of Business (application number ISB-IRB 2022‐04) and were approved on January 28, 2022. Informed consent was obtained from the participants before recording their responses. Information provided to participants included the purpose of the study, what participants will be asked to do if they participate, how much of the subjects’ time participation may take, and what type of information they might be asked to provide. It was also made clear that participation is voluntary, and subjects may withdraw from the study at any time. The data were deidentified to protect participants’ privacy. The consent form can be referenced in Multimedia Appendix 4.

## Results

### Descriptive Statistics

We found that over 90% of AIPs believed in the benefits of AI, with 93.8% (270/288) responding positively to “AI Improves Accuracy of TB Diagnosis,” and 90.9% (262/288) responding positively to “AI Can Detect Image Findings.” We also found that 69.4% (200/288) of AIPs were willing to try AI. For those who believed in AI, 71.9% (194/270) were willing to try AI.

Of the final 288 observations used for regression analysis, 73.3% (211/288) were from Gujarat, and 27.7% (77/288) were from Jharkhand. In the sample, 11.1% (32/288) were women, and 88.9% (256/288) were men with ages between 22 and 68 (mean 40.7, SD 9.7) years. See Table 2 for the summary statistics of the data.

**Table 2.** Summary statistics for each variable in regression analysis, for Jharkhand and Gujarat, February-September 2022.

	Jharkhand (n=77)^a^				Gujarat (n=211)^b^			
Variable	Mean	SD	Min	Max	Mean	SD	Min	Max
Age (years)	40.87	10.92	24	67	40.60	9.47	22	68
AI^c^ Intent	0.58	0.50	0	1	0.73	0.44	0	1
AI Belief	4.39	0.71	2.5	5	4.59	0.55	3	5
Confidence in diagnosing TB^d^	4.41	0.46	3	5	4.50	0.50	2.4	5
Trust in local radiologists	4.56	0.62	1.33	5	4.85	0.34	3.33	5

a Of the 77 respondents, 61 (79.2%) were male and 16 (20.8) were female.

b Of the 211 respondents, 195 (92.4%) were male and 16 (7.6%) were female.

c AI: artificial intelligence.

d TB: tuberculosis.

The mean score for “Confidence in diagnosing TB” was similar in both states, 4.50 (SD 0.50) for Gujarat and 4.41 (SD 0.46) for Jharkhand. The mean score for “Trust in local radiologists” was higher among AIPs in Gujarat (4.85, SD 0.34) than in Jharkhand (4.56, SD 0.62).

The Cronbach α value for the questions measuring “Confidence in diagnosing TB” was 0.65, while it was 0.82 for “Trust in the local radiologist” and 0.79 for “AI Belief.”

### AI Intent

The results of the logistic regression analysis of “AI Intent” on predictor variables are shown in Table 3. We found that “Confidence in diagnosing TB” was positively associated for both states but statistically significant only for Gujarat (odds ratio [OR] 1.943, 95% CI 1.038-3.637; *P*=.04) and not for Jharkhand (OR 2.186, 95% CI 0.695-6.875; *P*=.18).

**Table 3.** Regression results for dependent variable “AI Intent”, for Jharkhand and Gujarat, February-September 2022.

	Jharkhand (n=77)				Gujarat (n=211)			
Predictor variables	Odds ratio	SE	*P* value	95% CI	Odds ratio	SE	*P* value	95% CI
Age (years)	0.964	0.025	.15	0.916-1.014	0.990	0.016	.52	0.959-1.021
Gender	2.573	1.874	.20	0.617-10.728	2.073	1.252	.23	0.635-6.771
Confidence in diagnosing TB^a^	2.186	1.278	.18	0.695-6.875	1.943	0.622	.04	1.038-3.637
Trust in local radiologists	2.107	0.914	.09	0.900-4.932	0.149	0.117	.02	0.032-0.691

a TB: tuberculosis.

“Trust in local radiologists” was positively associated with “AI Intent” for AIPs in Jharkhand (OR 2.107, 95% CI 0.9-4.932; *P*=.09) but negatively associated for AIPs in Gujarat (OR 0.149, 95% CI 0.032‐0.691; *P*=.02).

### AI Belief

The results of the multivariate linear regression (ordinary least squares) analysis are shown in Table 4. First, we observed that “Confidence in diagnosing TB” was positively associated with “AI Belief” in both Jharkhand (β=0.72, *P*<.001) and Gujarat (β=0.177, *P*=.008). Further, “Trust in local radiologists” was also positively associated with “AI Belief” in both Jharkhand (β=0.215, *P*=.02) and Gujarat (β=0.66, *P*<.001).

**Table 4.** Regression results for dependent variable “AI Belief”, for Jharkhand and Gujarat, February-September 2022.

	Jharkhand (n=77)				Gujarat (n=211)			
Predictor variables	β	SE	*P* value	95% CI	β	SE	*P* value	95% CI
Age (years)	−0.007	0.006	.24	−0.020 to 0.005	0.002	0.004	.64	−0.005 to 0.009
Gender	0.166	0.196	.40	−0.225 to 0.557	0.021	0.105	.84	−0.186 to 0.227
Confidence in diagnosing TB^a^	0.720	0.137	<.001	0.447 to 0.993	0.177	0.066	.008	0.047 to 0.308
Trust in local radiologists	0.215	0.089	.02	0.037 to 0.392	0.660	0.105	<.001	0.453 to 0.866

a TB: tuberculosis.

We found that none of the demographic factors were significantly associated with “AI Belief.”

## Discussion

### Principal Findings

The integration of AI with CXRs holds promise as a cost-effective and accurate solution for TB diagnosis. However, despite this potential, the adoption of AI tools among health care providers for TB diagnosis remains limited. In this study, we conducted a cross-sectional survey of AIPs in 2 Indian states (Gujarat and Jharkhand) to understand and assess their belief in AI; their willingness to adopt AI for improved diagnosis; and the association of these with the AIPs’ confidence in diagnosing TB as well as trust in the local radiologists. We found that AIPs are mostly aware of AI and believe in AI, but the intent to adopt AI is lower and depends on AIPs’ confidence in diagnosing TB and their trust in local radiologists. Previous studies that examined the perceptions of health care professionals about AI in the medical field have yielded mixed results [37,38]. For instance, Oh et al [37] found that physicians in Korea had a favorable attitude toward AI, considering it as a complementary tool rather than a replacement for their roles. In contrast, Abdullah et al [38] discovered that health care employees in Saudi Arabia needed to be made aware of the benefits of AI and expressed concerns about being replaced by AI in their jobs.

While previous studies primarily focused on formal health care professionals [37-39], students [40], and the general public [41], our study contributes to understanding perceptions of AI among other medical professionals, specifically AIPs in India. Our findings indicate that most AIPs (over 90%) believed in AI’s capabilities; however, a significant proportion (88/288, 30.6%) were unwilling to try AI. These findings align with previous research among physicians [42], where the intention to use AI was observed only when there was a belief in the role of AI. However, unlike in prior research [42], where the participants were medical college students who received AI education as part of their curriculum, AIPs are unlikely to have similar formal educational opportunities for exposure to AI. Therefore, AIPs would require additional support, for example, in terms of training programs to know about AI and subsequently try AI. Additionally, AI systems should strive to be more explainable to make it easier to believe in AI, for example, by providing underlying reasoning that leads to their output, which is also known as explainable AI. An explainable AI approach would assist AIPs in developing trust in the system’s output and bolster their belief in AI.

Our study has uncovered a noteworthy association between AIPs’ confidence in diagnosing TB and their willingness to adopt AI, which is somewhat surprising when considering prior research has indicated that highly confident clinicians are less likely to change their decision [43]. However, AIPs differ from general clinicians in a crucial aspect, as they lack formal training in reading x-rays films. Therefore, we posit that the confidence of AIPs is likely influenced by the availability of high-quality diagnostic inputs, such as access to microbiological tests and well-prepared radiology reports. While the contextual factors may be at play in our findings, this suggests that the overall impact of AI adoption on health care outcomes might be more restrained than earlier studies have suggested. This distinction arises from the fact that those choosing to adopt AI may already have access to a high-quality diagnostic infrastructure, and thus, their adoption of AI might not significantly boost health outcome metrics like case detection rates. In light of this insight, it is advisable for companies and health care organizations to not rely exclusively on mean performance differences between AI systems and radiologists when estimating the potential enhancements in health outcomes.

Moreover, we identified a substantial difference in the intention to adopt AI between the states of Gujarat and Jharkhand. A higher willingness to try AI was observed among respondents from Gujarat compared to their counterparts in Jharkhand. The observed disparity between the 2 states could be attributed to Gujarat being a more developed state in India, with higher levels of education and better health care infrastructure. The presence of a well-established, technology-enabled infrastructure in Gujarat may have provided first-hand experience of the benefits of technology in health care, leading to a stronger intent to try technological products like AI. This implies that, in the context of AIPs, states with better technology-based infrastructure are the ones likely to have higher adoption when AI-based systems are implemented on the ground.

Another factor that might have affected willingness to try AI was the unavailability of qualified radiologists in less developed states like Jharkhand. One would expect AIPs to be more willing to try AI systems in the absence of qualified radiologists. However, our findings indicate that AIPs in Jharkhand were less likely to try AI. The reasons for this attitude are not immediately apparent, but experiencing lower-quality radiology reports may bias AIPs into believing that CXR technology is unreliable. To overcome such barriers to adoption, implementing agencies will need to provide additional evidence of the benefits of AI and related technologies like CXR scans.

By integrating AI into the primary health care centers, which are already actively engaged in TB service provision, one can extend AI-powered TB diagnostics to grassroots levels [15]. While AIPs acknowledge the potential of AI to enhance TB diagnosis, concerns regarding the financial impact of such technology are significant. AI’s integration into TB diagnosis through CXR screenings entails an additional cost, estimated at a modest ₹100 (approximately $1.3) per screening [44]. This cost, while relatively small, would be borne by AIPs and possibly transferred to patients. Considering India’s per capita net income was ₹98,374 (US $1138.71) in 2022‐23 [45], this expense may be manageable for a large population segment. However, affordability may pose a barrier in economically disadvantaged regions, potentially necessitating government intervention to facilitate access. The urgency of integrating AI in diagnostics must be contextualized within India’s acute radiologist shortfall, with the current ratio standing at approximately one radiologist per 100,000 people, starkly below international norms [15]. This shortage underscores the broader necessity of AI deployment in augmenting health care capacity.

Our study has unveiled several important findings that are instrumental in understanding the landscape of AI in India’s TB-related health care services. First and foremost, a significant fraction of AIPs have a positive attitude toward AI, with many expressing a readiness to integrate AI into their practice. However, we have identified a discernible gap between those who acknowledge AI’s benefits and those who are prepared to embrace the technology. This discrepancy is further nuanced by a variation in attitudes based on providers’ self-assessed diagnostic competencies, providers with greater confidence in their abilities tend to be more receptive to AI. Additionally, there is noticeable heterogeneity in the willingness to adopt AI across different geographic regions, which points to the influence of local health care infrastructure and socioeconomic factors on technology acceptance.

The implications of these findings are multifaceted and suggest several actionable strategies for enhancing AI adoption in health care settings. To translate the positive attitudes of AIPs into actual use of AI, tailored initiatives that address barriers to implementation are necessary. This might include providing more robust evidence of AI’s effectiveness, integrating AI education into continuous professional development programs, and offering financial incentives. As diagnostic pathways in TB are majorly driven by the private sector [27], steps should be taken to make the integration of AI in the TB care cascade profitable for providers to ensure its sustainability. Moreover, there is a compelling need to target AIPs with suboptimal diagnostic capabilities, as enhancements in this group’s diagnostic services could positively impact overall health care outcomes.

To ensure these strategies are effective across India’s diverse health care landscape, future research should replicate this study in multiple geographies. Such research should also examine the intricacies of trust and financial relationships between AIPs and radiologists to uncover deeper insights into the factors that influence AI adoption and to devise informed interventions that can bolster the technology’s uptake.

### Limitations

Like other survey-based studies, this study too has a few limitations. First, we had to remove many observations for the state of Jharkhand because many AIPs in Jharkhand did not respond to questions on “Trust in local radiologists.” The lower response rate to these questions was because of AIPs’ limited access to radiologists. To ascertain if the removal of such AIPs from the data impacted the findings, we compared the demographic variables of the dataset before and after the removal and did not find any statistically significant difference (more information in Multimedia Appendix 5).

Second, the responses in the questionnaire that measured opinions about AI may have been subjected to social desirability bias among AIPs. To mitigate this effect, we informed the participants about the anonymity of the survey and the separation of personal information from their responses.

Third, the AIPs’ perception of AI in TB diagnosis may have been influenced by their overall opinion of AI and the extent of their knowledge of AI. To allow more informed responses, participants were apprised of the AI approach to recognize TB cases.

### Conclusions

Our study provides insights into the attitudes of AYUSH and informal health care providers toward AI in TB diagnosis. It contributes to the understanding that adopting AI is not solely determined by a belief in AI but also relates to factors like confidence in diagnosing TB and other local factors like the quality of radiology services. Furthermore, regional variations between Gujarat and Jharkhand underscore the importance of tailored interventions to effectively integrate AI into diagnostic practices, with implications beyond TB diagnosis.

## Supplementary material

10.2196/54156 Multimedia Appendix 1 Application form to map Ayurveda, Yoga and Naturopathy, Unani, Siddha, and Homoeopathy practitioners and informal health care providers.

10.2196/54156 Multimedia Appendix 2 Survey Questionnaire.

10.2196/54156 Multimedia Appendix 3 Outlier analysis.

10.2196/54156 Multimedia Appendix 4 Institutional review board application and approval documents.

10.2196/54156 Multimedia Appendix 5 Descriptor variables.

10.2196/54156 Checklist 1 STROBE (Strengthening the Reporting of Observational Studies in Epidemiology) checklist.

## Abbreviations

AI: artificial intelligence
AIP: Ayurveda, yoga and naturopathy, Unani, Siddha, and homoeopathy practitioner and informal health care provider
AYUSH: Ayurveda, Yoga and Naturopathy, Unani, Siddha, and Homoeopathy
CXR: chest x-ray
LMIC: low- and middle-income country
MTB/RIF: *Mycobacterium tuberculosis*/rifampin
OR: odds ratio
TB: tuberculosis
WHO: World Health Organization
WHP: World Health Partner
