# Supplementary material for: Understanding Providers’ Attitude Toward AI in India’s Informal Health Care Sector: Survey Study
Source: JMIR Form Res. 2025 Feb 10;9:e54156. doi: 10.2196/54156 (PMC11832356; doi:10.2196/54156)
Supplement: Multimedia Appendix 1 [file formative-v9-e54156-s001.pdf]

Mapping of Healthcare Providers

1. Name of facility: ____
2. Number of Providers (Below data fields triggered for each provider): _______________
3. Name of provider: ____
4. Address: ____
5. Landmark: ____
6. Mobile number #1:______________________Mobile number #2:_______________
7. Qualification (MD- Internal Medicine/MD-Pulmonologist/ DTCD/ID physician/MBBS/other)
    1. ______________________ (Please write if other)
8. Days/Hours of operation: ____
9. Estimated number of patients seen/ day (overall): ______________________________
10. Estimated number of cough/fever seen per month: _____________________________
11. Estimated number of diagnosed TB cases per month: ___________________________
12. Has a HFID? (If yes, enter HFID): ____
13. Notifies TB cases to Ni-kshay? (himself/herself or through Hub agent/Or handing over to Govt. on monthly basis)
14. Does this provider have any tie up with any lab for x-ray?
    1. If yes, name of the x-ray facility: ____
15. Does this provider work at another facility?
    1. If yes, details of other facilities: ____
    2. Name of Facility: ____
    3. Address: ____
16. Targeted (yes/no): ____
17. Willing to engage? (yes/no): ____
18. Engaged (yes/no): ____
19. Status
    1. Open
    2. Shut down
    3. Relocated
20. GPS: ____
