# Supplementary material for: Understanding Providers’ Attitude Toward AI in India’s Informal Health Care Sector: Survey Study
Source: JMIR Form Res. 2025 Feb 10;9:e54156. doi: 10.2196/54156 (PMC11832356; doi:10.2196/54156)
Supplement: Multimedia Appendix 2 [file formative-v9-e54156-s002.pdf]

Question by Question Guide for ISB- TB Study

आईएसबी-टीबी अध्ययन के लिए प्रश्न द्वारा प्रश्न गाइड

Enumerator Name<br><br>सर्वेयर का नाम<br><br>નામ		You need to write your name in the section provided.<br><br>आपको दिए गए सेक्शन में अपना नाम लिखना होगा।
Respondent ID<br><br>प्रतिवादी आईडी<br><br>પ્રતિવાદી ID		Please enter the respondent ID for the given provider as per the list you have been provided with.<br><br>आपको प्रदान की गई सूची के अनुसार कृपया दिए गए प्रदाता के लिए प्रतिवादी आईडी दर्ज करें।
Date and time of interview<br><br>साक्षत्कार की तारीख और समय<br><br>ઇન્ટરવ્યુની તારીખ અને સમય		Choose today's date, month and year from the drop down menu.<br><br>तारीख लिखने के लिए ड्रॉप डाउन मेनू से आज की तारीख, महीना और साल चुनें।
Please read out the consent form to the respondent so that you can take the required consent to proceed with the interview.<br><br>कृपया प्रतिवादी को सहमति प्रपत्र पढ़ें ताकि आप साक्षात्कार के लिए आगे बढ़ने के लिए आवश्यक सहमति ले सकें.<br><br>કૃપા કરીને પ્રતિવાદીને સંમતિ ફોર્મ વાંચો જેથી તમે ઇન્ટરવ્યુમાં આગળ વધવા માટે જરૂરી સંમતિ લઈ શકો.		
INDIAN SCHOOL OF BUSINESS CONSENT FORM.<br>In this research, you will answer a few survey questions that would help the researchers to understand your attitude towards technology. You will be provided a list of questions that you can answer in order. Your answers will allow the researchers to find when an Artificial Intelligence (AI) software would be helpful for Tuberculosis (TB) diagnosis. As an incentive to participate in this survey, we will offer a mobile recharge valid for 28 days. The approximate recharge value would be somewhere between Rs 260 and Rs 360, depending on your network provider and your location. In case of any confusion, please talk to the survey administrator.		Please read this information form to the respondent to let you know who you are, the purpose of the survey, who is doing it, the ethical protocols being followed and the incentive being given.<br><br>आप कौन हैं, सर्वेक्षण का उद्देश्य, कौन कर रहा है, नैतिक प्रोटोकॉल का पालन किया जा रहा है और प्रोत्साहन दिया जा रहा है, यह बताने के लिए कृपया प्रतिवादी को यह सूचना प्रपत्र पढ़ें।

The expected duration of this survey is 10-20 minutes. The survey will be performed at the location of the participant, and those conducting the survey, will travel to the location of the participant.<br><br>No other information apart from the survey answers will be collected from the participants. All answers will be kept confidential by separating the information you provide from your personal information. Nobody other then the researcher will know what you answered. We request you to provide us with honest responses to all questions. While conducting this survey, in addition to answers, we will also take audio recordings to verify the conversations. Only the survey administrators, PIs and Co-PIs of the study will have access to the survey answers and recordings.<br><br>Participation in the research is completely voluntary. If there is any question you don't want to answer or if at any point you feel uncomfortable with the study, you have the option of quitting the study. There will be no consequences for not completing the study.<br><br>There are no known risks associated with your participation in this research beyond those of everyday life. Your participation will help the research since your views are important.<br><br>If there is anything about the study or your participation that is unclear or that you do not understand, if you have questions or wish to report a research–related problem, you may contact the Principal Investigator: Sarang Deo at phone 9640233211or email sarang_deo@isb.edu at the Indian School of Business, Gachibowli, Hyderabad – 500032, India.<br><br>For questions about your rights as a research participant, you may contact the Chair of the Institutional Review Board (IRB) at ISB: Professor Ashwini Chhatre at 040-2318-7134 or email ashwini_chhatre@isb.edu at the Indian School of Business, Gachibowli, Hyderabad – 500111, India.<br><br><br>इंडियन स्कूल ऑफ बिजनेस कंसेंट फॉर्म<br><br>इस अध्ययन में, आप कुछ प्रश्नो के उत्तर देंगे जो अध्ययनकर्ताओं को टेक्नोलॉजी के प्रति आपके रवैया<br>को समझने में मदद करेंगे। आपको प्रश्नों की एक सूची प्रदान की जाएगी जिनका आप क्रम से उत्तर देते जायेंगे। आपके उत्तर अध्ययनकर्ताओं को यह पता लगाने में मदद करेंगे कि किन परिस्थितियों में एक आर्टिफिशियल इंटेलिजेंस (एआई) सॉफ्टवेयर क्षय रोग (टीबी) के जांच के लिए सहायक होगा। इस सर्वे में भाग लेने के लिए प्रोत्साहन के रूप में, हम 28 दिनों के लिए वैध मोबाइल रिचार्ज की पेशकश करेंगे। आपके नेटवर्क प्रदाता और आपके	

स्थान के आधार पर अनुमानित रिचार्ज मूल्य 260 रुपये और 360 रुपये के बीच होगा। अगर आपको कोई भी प्रश्न हैं, कृपया सर्वे व्यवस्थापक से बात करें।

इस सर्वे की अपेक्षित अवधि 10-20 मिनट है। सर्वे प्रतिभागी के स्थान पर किया जाएगा, और सर्वे करने वाले प्रतिभागी के स्थान पर जायेंगे।

प्रतिभागियों से सर्वे के उत्तरों के अलावा कोई अन्य जानकारी एकत्र नहीं की जाएगी। आपके द्वारा प्रदान की गई जानकारी को आपकी व्यक्तिगत जानकारी से अलग करके सभी उत्तरों को गोपनीय रखा जाएगा। अध्ययनकर्ता के अलावा कोई और नहीं जान पाएगा कि आपने क्या उत्तर दिया। हम आपसे अनुरोध करते हैं कि आप हमें सभी प्रश्नों के उत्तर ईमानदारी से प्रदान करें। इस सर्वे को करते समय, हम सारे उत्तर और बातचीत के प्रमाण के रूप में ऑडियो रिकॉर्डिंग भी लेंगे। केवल सर्वे प्रशासकों, अध्ययन के पीआई और सह-पीआई के पास सर्वे के उत्तरों और रिकॉर्डिंग की जानकारी होगी।

अध्ययन में भाग लेना पूरी तरीके से स्वैच्छिक है। यदि कोई ऐसा प्रश्न है जिसका आप उत्तर नहीं देना चाहते हैं या यदि किसी भी वक़्त आप अध्ययन से असहज महसूस करते हैं, तो आपके पास अध्ययन छोड़ने का विकल्प है। अध्ययन पूरा नहीं करने का कोई परिणाम नहीं होगा।

इस अध्ययन में भाग लेने में कोई जोखिम नहीं है। आपकी भागीदारी से अध्ययन में मदद मिलेगी क्योंकि आपके विचार महत्वपूर्ण हैं।

यदि इस अध्ययन या आपकी भागीदारी के बारे में कुछ ऐसा है जो अस्पष्ट है या आपको समझ में नहीं आता है, यदि आपके कोई प्रश्न हैं या अध्ययन से संबंधित समस्या को रिपोर्ट करना चाहते हैं, तो आप प्रधान अन्वेषक: सारंग देव से फोन 9640233211 या ईमेल sarang_deo@isb.edu पर इंडियन स्कूल ऑफ बिजनेस, गचीबावली, हैदराबाद - 500032, भारत, पर संपर्क कर सकते हैं।

एक अध्ययन प्रतिभागी के रूप में अपने अधिकारों के बारे में प्रश्नों के लिए, आप आईएसबी में संस्थागत समीक्षा बोर्ड (आईआरबी) के अध्यक्ष से संपर्क कर सकते हैं: प्रोफेसर अश्विनी छत्रे 040-2318-7134 पर या इंडियन स्कूल ऑफ बिजनेस, गाचीबोवली, हैदराबाद - 500111, भारत में ashwini_chhatre@isb.edu पर ईमेल करें।

ઇન્ડિયન સ્કૂલ ઑફ બિઝનેસ કન્સેન્ટ ફોર્મ

ઈન્ડિયન સ્કૂલ ઓફ બિઝનેસ, ઈન્ડિયા દ્વારા આયોજિત આ અભ્યાસમાં ભાગ લેવા માટે સંમત થવા બદલ આભાર. આ સંશોધનમાં, તમે સર્વેક્ષણના અમુક પ્રશ્નોના જવાબ આપશો જે સંશોધકોને ટેક્નોલોજી પ્રત્યેના તમારા વલણને સમજવામાં મદદ કરશે. તમને પ્રશ્નોની સૂચિ આપવામાં આવશે જેનો તમે ક્રમમાં જવાબ આપી શકશો. તમારા જવાબો સંશોધકોને આર્ટિફિશિયલ ઈન્ટેલિજન્સ (AI) સોફ્ટવેર ટ્યુબરક્યુલોસિસના

(ટીબી) નિદાન માટે ક્યારે મદદરૂપ થશે એ શોધવામાં મદદ કરશે. આ સર્વેક્ષણમાં ભાગ લેવા માટે પ્રોત્સાહન તરીકે, અમે 28 દિવસ માટે માન્ય મોબાઇલ રિચાર્જ કરીશું. તમારા નેટવર્ક પ્રદાતા અને તમારા સ્થાનના આધારે અંદાજિત રિચાર્જ મૂલ્ય રૂ. 260 અને રૂ. 360 ની વચ્ચે હશે. કોઈપણ મૂંઝવણના કિસ્સામાં, કૃપા કરીને સર્વે એડમિનિસ્ટ્રેટર સાથે વાત કરો.

આ સર્વેક્ષણનો અપેક્ષિત સમયગાળો 10-20 મિનિટનો છે. સર્વેક્ષણ સહભાગીના સ્થાન પર કરવામાં આવશે, અને સર્વેક્ષણ હાથ ધરનારાઓ, સહભાગીના સ્થાન પર મુસાફરી કરશે.

સર્વેક્ષણના જવાબો સિવાય સહભાગીઓ પાસેથી અન્ય કોઈ માહિતી એકત્રિત કરવામાં આવશે નહીં. તમે આપેલી માહિતીને તમારી અંગત માહિતીથી અલગ કરીને તમામ જવાબો ગુપ્ત રાખવામાં આવશે. તમે શું જવાબ આપ્યો તે સંશોધક પછી અન્ય કોઈ જાણશે નહીં. અમે તમને વિનંતી કરીએ છીએ કે અમને બધા પ્રશ્નોના પ્રમાણિક જવાબો આપો. આ સર્વેક્ષણ કરતી વખતે, જવાબો ઉપરાંત, અમે વાતચીતને ચકાસવા માટે ઑડિયો રેકોર્ડિંગ પણ લઈશું. માત્ર સર્વે એડમિનિસ્ટ્રેટર્સ, પીઆઈ અને અભ્યાસના સહ-પીઆઈને જ સર્વેક્ષણના જવાબો અને રેકોર્ડિંગની ઍક્સેસ હશે.

સંશોધનમાં ભાગીદારી સંપૂર્ણપણે સ્વૈચ્છિક છે. જો એવો કોઈ પ્રશ્ન હોય કે જેનો તમે જવાબ આપવા માંગતા ન હોવ અથવા કોઈપણ સમયે તમે અભ્યાસમાં અસ્વસ્થતા અનુભવો છો, તો તમારી પાસે અભ્યાસ છોડી દેવાનો વિકલ્પ છે. અભ્યાસ પૂર્ણ ન કરવા પર કોઈ વાંધોઆવશે નહીં.

રોજિંદા જીવનની બહાર આ સંશોધનમાં તમારી સહભાગિતા સાથે સંકળાયેલા કોઈ જાણીતા જોખમો નથી. તમારી ભાગીદારી સંશોધનમાં મદદ કરશે કારણ કે તમારા મંતવ્યો મહત્વપૂર્ણ છે.

જો આ અભ્યાસ અથવા તમારી સહભાગિતા વિશે કંઈપણ અસ્પષ્ટ હોય અથવા તમે સમજી શકતા નથી, જો તમને પ્રશ્નો હોય અથવા સંશોધન-સંબંધિત સમસ્યાની જાણ કરવા માંગતા હો, તો તમે મુખ્ય તપાસનીશ: સારંગ દેવનો ફોન 9640233211 અથવા ઇમેઇલ sarang_deo@isb.edu પર સંપર્ક કરી શકો છો. ઇન્ડિયન સ્કૂલ ઑફ બિઝનેસ, ગાચીબોવલી, હૈદરાબાદ – 500032, ભારત ખાતે.

સંશોધન સહભાગી તરીકે તમારા અધિકારો વિશેના પ્રશ્નો માટે, તમે ISB પર સંસ્થાકીય સમીક્ષા બોર્ડ (IRB) ના અધ્યક્ષનો સંપર્ક કરી શકો છો: પ્રોફેસર અશ્વિની છત્રેનો 040-2318-7134 પર અથવા ashwini_chhatre@isb.edu પર ઇમેઇલ કરો. ઇન્ડિયન સ્કૂલ ઑફ બિઝનેસ, ગચીબોવલી ખાતે હૈદરાબાદ – 500111, ભારત.
મહત્વપૂર્ણ છે.

જો આ અભ્યાસ અથવા તમારી સહભાગિતા વિશે કંઈપણ અસ્પષ્ટ હોય અથવા તમે સમજી શકતા નથી, જો તમને પ્રશ્નો હોય અથવા સંશોધન-સંબંધિત સમસ્યાની જાણ કરવા માંગતા હો, તો તમે મુખ્ય તપાસનીશ: સારંગ દેવનો ફોન 9640233211 અથવા ઇમેઇલ sarang_deo@isb.edu પર સંપર્ક કરી શકો છો. ઇન્ડિયન સ્કૂલ ઑફ

<table>
<tr><td colspan="4">બિઝનેસ, ગાચીબોવલી, હૈદરાબાદ – 500032, ભારત ખાતે.

સંશોધન સહભાગી તરીકે તમારા અધિકારો વિશેના પ્રશ્નો માટે, તમે ISB પર સંસ્થાકીય સમીક્ષા બોર્ડ (IRB) ના અધ્યક્ષનો સંપર્ક કરી શકો છો: પ્રોફેસર અશ્વિની છત્રેનો 040-2318-7134 પર અથવા ashwini_chhatre@isb.edu પર ઇમેઇલ કરો. ઇન્ડિયન સ્કૂલ ઑફ બિઝનેસ, ગચીબોવલી ખાતે હૈદરાબાદ – 500111, ભારત.</td><td></td></tr>
<tr><td colspan="2" rowspan="2">Do you give consent to participate in the study?

क्या आप अध्ययन में भाग लेने के लिए सहमति देते हैं?

શું તમે અભ્યાસમાં ભાગ લેવા માટે સંમતિ આપો છો?</td><td colspan="2">1 Yes

हाँ

હા</td><td rowspan="2">Please ask the respondent for their consent and only if they say yes , then proceed with the survey.

कृपया प्रतिवादी से उनकी सहमति के लिए पूछें और यदि वे हाँ कहते हैं, तो ही सर्वेक्षण के लिए आगे बढ़ें।</td></tr>
<tr><td colspan="2">2 No

नहीं

ना</td></tr>
<tr><td colspan="5">Section:1 TB Knowledge

अनुभाग: 1 टीबी के बारे में जानकारी

વિભાગ 1: ટીબીનું જ્ઞાન</td></tr>
<tr><td>S.No</td><td>Questions

प्रशन</td><td colspan="2">Options

विकल्प</td><td>Notes for Enumerators

सर्वेयर के लिए नोट्स</td></tr>
<tr><td rowspan="3">1.0</td><td rowspan="3">Which of the following is NOT a symptom of TB?

निम्न में से कौन-सा टीबी का लक्षण नहीं है?

નીચેનામાંથી કયું ટીબીનું લક્ષણ નથી?</td><td>1</td><td>Chest pain

सीने में दर्द

છાતીમાં દુખાવો</td><td rowspan="3">Please understand that Tuberculosis (TB) is a potentially serious infectious disease that mainly affects the lungs. The bacteria that cause tuberculosis are spread from person to person through tiny droplets released into the air via coughs and sneezes.

कृपया समझें कि क्षय रोग (टीबी) एक सभावित गंभीर संक्रामक रोग है जो मुख्य रूप से फेफड़ों को प्रभावित करता है। तपेदिक का कारण बनने वाले जीवाणु खांसी और छींक के माध्यम से हवा में छोड़ी गई छोटी बूंदों के माध्यम से एक व्यक्ति से दूसरे व्यक्ति में फैलते हैं।</td></tr>
<tr><td>2</td><td>Fatigue

थकान

થાક</td></tr>
<tr><td>3</td><td>Shortness of breath

सांस की तकलीफ

શ્વાસની તકલીફ</td></tr>
</table>

		4	Weight gain/Increased appetite<br><br>वजन बढ़ना / भूख में वृद्धि<br><br>વજન વધવું/ભૂખમાં વધારો	This question aims to know which of the following is not a symptom of TB.<br><br>इस प्रश्न का उद्देश्य यह जानना है कि निम्न में से कौन-सा टीबी का लक्षण नहीं है।<br><br>Please ask the respondent which of the following is not a symptom of TB.<br><br>कृपया प्रतिवादी से पूछें कि निम्न में से कौन-सा टीबी का लक्षण नहीं है।<br><br>*Read out the options to the respondent and ask him/her to choose accordingly-*<br><br>1. Chest pain<br>2. Fatigue- Please note that fatigue is a condition where the person feels extremely tired.<br>3. Shortness of breath<br>4. Weight gain/Increased appetite<br><br>प्रतिवादी के लिए विकल्पों को पढ़ें और उसे तदनुसार चुनने के लिए कहें-<br><br>1. सीने में दर्द<br>2. थकान<br>3. सांस की तकलीफ<br>4. वजन बढ़ना / भूख में वृद्धि
1.1	What is the recommended duration of the TB treatment for a pulmonary drug-sensitive TB case?<br><br>पल्मोनरी ड्रग-सेंसिटिव टीबी केस के इलाज का अनुमानित समय क्या है?<br><br>પલ્મોનરી ડ્રગ-સંવેદનશીલ ટીબી કેસની સારવાર માટે અંદાજી સમય કેટલો છે?	1	<3 months<br><br><3 महीने<br><br><3 મહિના	**Pulmonary TB (PTB)** refers to any bacteriologically confirmed or clinically diagnosed case of TB involving the lung.<br><br>पल्मोनरी टीबी (पीटीबी) फेफड़े से जुड़े टीबी के किसी भी बैक्टीरियोलॉजिकल रूप से पुष्टि या नैदानिक रूप से निदान किए गए मामले को संदर्भित करता है।<br><br>This question aims to understand the duration of TB treatment for a Pulmonary drug-sensitive TB case.
		2	3-5 months<br><br>3-5 महीने<br><br>3-5 મહિના	
		3	6 months	

			6 महीने<br><br>6 મહિના	इस प्रश्न का उद्देश्य पल्मोनरी ड्रग-सेंसिटिव टीबी केस के लिए टीबी उपचार की अवधि को समझना है।<br><br>Please ask the respondent what is the recommended duration of treatment for a person suffering from a pulmonary drug-sensitive TB case.<br><br>कृपया प्रतिवादी से पूछें कि फुफ्फुसीय दवा-संवेदनशील टीबी मामले से पीड़ित व्यक्ति के लिए उपचार की अनुशंसित अवधि क्या है।<br><br>Probe further to know for how many months the treatment continues.<br><br>कितने महीनों तक इलाज चलता है, यह जानने के लिए आगे की जांच करें।
		4	7-9 months<br><br>7-9 महीने<br><br>7-9 મહિના	
		5	>9 months<br><br>>9 महीने<br><br>>9 મહિના	
**Section: 2** ***Diagnosis and Treatment of TB***<br><br>अनुभाग***: 2*** टीबी का निदान और उपचार<br><br>વિભાગ ***2:*** ટીબીનું નિદાન અને સારવાર				
2.1	What is your opinion about chest X-Ray as a diagnostic tool for pulmonary TB?<br><br>पल्मोनरी-टीबी के निदान उपकरण के रूप में छाती के एक्स-रे के बारे में आपकी क्या राय है?<br><br>પલ્મોનરી ટીબીના નિદાનના સાધન તરીકે છાતીના એક્સ-રે વિશે તમારો શું અભિપ્રાય છે?	1	Chest X-Rays are not a good tool for diagnosing TB<br><br>टीबी के निदान के लिए चेस्ट एक्स-रे एक अच्छा उपकरण नहीं है<br><br>છાતીના એક્સ-રે એ ટીબીના નિદાન માટે સારું સાધન નથી	You know what pulmonary TB is. Please understand the following terms before you ask this question to the respondent-<br><br>आप जानते हैं कि पल्मोनरी-टीबी क्या है। प्रतिवादी से यह प्रश्न पूछने से पहले कृपया निम्नलिखित शर्तों को समझ लें-<br><br>**Diagnostic tools** are equipment used for discovering what is wrong with people who are ill.<br><br>नैदानिक उपकरण ऐसे उपकरण हैं जिनका उपयोग यह पता लगाने के लिए किया जाता है कि बीमार लोगों के साथ क्या गलत है।<br><br>Chest X-ray is an imaging test used to examine the structures and organs in your chest. It helps diagnose symptoms such as-
		2	Chest X-Rays can suggest the presence of active TB in a patient<br><br>चेस्ट एक्स-रेरोगी में सक्रिय टीबी की उपस्थिति का सुझाव दे सकता है	

<table>
<tr><td rowspan="3"></td><td rowspan="3"></td><td></td><td>છાતીના એક્સ-રે દર્દીમાં સક્રિય ટીબીની હાજરી સૂચવી શકે છે</td><td rowspan="3">Breathing difficulties
Bad or persistent cough
Chest pain or injury
चेस्ट एक्स-रे एक इमेजिंग टेस्ट है जिसका उपयोग आपकी छाती में संरचनाओं और अंगों की जांच के लिए किया जाता है। यह लक्षणों का निदान करने में मदद करता है जैसे-
सांस लेने में तकलीफ
खराब या लगातार खांसी
सीने में दर्द या चोट
Here, we want to know the respondent’s opinion on how chest X-Rays work as a diagnostic tool for Pulmonary TB?

यहां, हम प्रतिवादी की राय जानना चाहते हैं कि कैसे छाती का एक्स-रे पल्मोनरी टीबी के निदान उपकरण के रूप में काम करता है?

Refer to the following explanation of options to understand how chest X-rays work as a diagnostic tool for detecting pulmonary TB.

फुफ्फुसीय टीबी का पता लगाने के लिए छाती के एक्स-रे कैसे नैदानिक उपकरण के रूप में काम करते हैं, यह समझने के लिए निम्नलिखित विकल्पों की व्याख्या देखें।
Chest X-Rays can suggest the presence of active TB in a patient- Mark this if the respondent says Chest X-Rays can confirm TB in a patient. Please note that this does not necessarily say that Chest X-Rays can successfully confirm TB.</td></tr>
<tr><td>3</td><td>Chest X-Rays can confirm active TB in a patient

चेस्ट एक्स-रे से रोगी में सक्रिय टीबी की पुष्टि हो सकती है

છાતીના એક્સ-રે દર્દીમાં સક્રિય ટીબીની પુષ્ટિ કરી શકે છે</td></tr>
<tr><td>-77</td><td>Others

अन्य

અન્ય</td></tr>
</table>

				चेस्ट एक्स-रे एक रोगी में सक्रिय टीबी की उपस्थिति का सुझाव दे सकता है- यदि प्रतिवादी कहता है कि चेस्ट एक्स-रे रोगी में टीबी की पुष्टि कर सकता है तो इसे चिह्नित करें। कृपया ध्यान दें कि यह जरूरी नहीं है कि चेस्ट एक्स-रे सफलतापूर्वक टीबी की पुष्टि कर सकता है।<br><br>2. **Chest X-**Rays can confirm active TB in a patient- You will mark this option if the respondent says chest X-rays fully confirms TB in the patient<br><br>चेस्ट एक्स-रे रोगी में सक्रिय टीबी की पुष्टि कर सकता है- यदि प्रतिवादी कहता है कि छाती का एक्स-रे रोगी में टीबी की पूरी तरह से पुष्टि करता है, तो आप इस विकल्प को चिह्नित करेंगे।<br><br>3. **Others-** You will only mark this option if the respondent says something which is not listed in the options above. Please note that if you mark this option, a box will open where you have to specify the option.<br><br>अन्य- आप इस विकल्प को केवल तभी चिह्नित करेंगे जब प्रतिवादी कुछ ऐसा कहता है जो ऊपर दिए गए विकल्पों में सूचीबद्ध नहीं है। कृपया ध्यान दें कि यदि आप इस विकल्प को चिह्नित करते हैं, तो एक बॉक्स खुलेगा जहां आपको विकल्प लिखना होगा।
2.1 a	If others, please specify<br>यदि अन्य, तो कृपया स्पष्ट करें<br><br>જો અન્ય, તો કૃપા કરીને સ્પષ્ટ કરો			This box will open if you click on ‘Others’ in the previous question. Please specify the other option.<br><br>यदि आप पिछले प्रश्न में 'अन्य' पर क्लिक करते हैं तो यह बॉक्स खुल जाएगा। कृपया विकल्प लिखे।

2.2	*Please indicate how much you agree with each of the statements.*<br><br>कृपया बताएं कि आप प्रत्येक कथन से कितना सहमत हैं?<br><br>કૃપા કરીને સૂચવો કે તમે દરેક વિધાન સાથે કેટલા સહમત છો?			This section focuses on understanding to what extent the respondent agrees with the following statements.<br><br>यह खंड यह समझने पर केंद्रित है कि प्रतिवादी किस हद तक निम्नलिखित कथनों से सहमत है।<br><br>You will have to read out the following statements to the respondent. Note that we will be explaining the statements in simple and easy language so that it becomes easier for you to explain the context of the question to the respondent.<br><br>आपको प्रतिवादी को निम्नलिखित कथनों को पढ़ना होगा। ध्यान दें कि हम कथनों को सरल और आसान भाषा में समझाएंगे ताकि आपके लिए उत्तरदाता को प्रश्न का संदर्भ समझाना आसान हो जाए।
2.2.1	I prefer to limit my contact with patients with TB<br><br>मैं टीबी के रोगियों के साथ अपने संपर्क को सीमित करना पसंद करता हूं<br><br>મને કોઈ વાંધો નથી કે, અન્ય લોકો જાણે છે કે હું ટીબીના દર્દીઓની સારવાર કરું છું	1	Strongly Disagree<br><br>पूरी तरह से असहमत<br><br>ભારપૂર્વક અસંમત	Please read out the statement to the respondent and ask her to what extent she agrees or disagrees with the statement. *Note that 'I' here signifies the respondent's view.*<br><br>कृपया प्रतिवादी को दिए गए कथन को पढ़ें और उससे पूछें कि वह कथन से किस हद तक सहमत या असहमत है। ध्यान दें कि यहां 'मैं' प्रतिवादी के विचार को दर्शाता है।<br><br>*"I prefer to reduce my contacts with TB patients"*<br><br>"मैं टीबी रोगियों के साथ अपने संपर्क कम करना पसंद करता हूं"<br><br>Refer to the explanations below to understand which options to mark accordingly even for the next set of
		2	Somewhat disagree<br><br>कुछ हद तक असहमत<br><br>થોડું બોવ અસંમત	
		3	Neither agree nor disagree<br><br>न सहमत और न ही असहमत<br><br>ન તો સંમત કે અસંમત	
		4	Somewhat agree	

			कुछ हद तक सहमत<br><br>થોડું બોવ સંમત	questions-<br><br>प्रश्नों के अगले सेट के लिए भी तदनुसार किन विकल्पों को चिह्नित करना है, यह समझने के लिए नीचे दी गई व्याख्याओं का समझ लें-<br><br>1. **Strongly Disagree-** Please mark this option if the respondent very strongly disagrees with the statement.<br><br>पूर्णतः असहमत - यदि प्रतिवादी कथन से अत्यधिक असहमत है तो कृपया इस विकल्प को चिन्हित करें।<br><br>2. **Somewhat disagree-** Mark this option if the respondent only disagrees with the statement to an extent.<br><br>कुछ हद तक असहमत - इस विकल्प को चिह्नित करें यदि प्रतिवादी केवल एक हद तक कथन से असहमत है।<br><br>3. **Neither agree nor disagree-** Please mark this option if the respondent does not agree or disagree with the statement at all.<br><br>न तो सहमत हैं और न ही असहमत- यदि प्रतिवादी कथन से बिल्कुल भी सहमत या असहमत नहीं है तो कृपया इस विकल्प को चिह्नित करें।<br><br>4. **Somewhat agree-** Please mark this option if the respondent agrees with the statement only to an extent.<br><br>कुछ हद तक सहमत - कृपया इस विकल्प को चिह्नित करें यदि प्रतिवादी केवल एक हद तक कथन
		5	Strongly Agree<br><br>पूरी तरह से सहमत<br><br>ભારપૂર્વક સંમત	

				से सहमत है।<br><br>5. **Strongly Agree-** Please mark this option if the respondent strongly agrees with the statement.<br><br>पूरी तरह से सहमत - कृपया इस विकल्प को चिह्नित करें यदि प्रतिवादी कथन से पूरी तरह से सहमत है।
2.2.2	I treat presumptive TB patients with the same kind of empathy as I would with any other patient<br><br>मैं अनुमानित टीबी रोगियों के साथ उसी तरह की हमदर्दी के साथ व्यवहार करता हूँ जैसे मैं किसी अन्य रोगी के साथ करता हूँ<br><br>હું અનુમાનિત ટીબીના દર્દીઓની એ જ પ્રકારની સહાનુભૂતિ સાથે સારવાર કરું છું જે રીતે હું અન્ય દર્દી નું કરું છું	1	Strongly Disagree<br><br>पूरी तरह से असहमत<br><br>ભારપૂર્વક અસંમત	Similar to the previous question you will read out the statement to the respondent and ask him/her to what extent they agree or disagree with the statement-<br><br>पिछले प्रश्न के समान ही आप प्रतिवादी को दिए गए कथन को पढ़ेंगे और उससे पूछेंगे कि वे कथन से किस हद तक सहमत या असहमत हैं-<br><br>*" I treat patients suffering from presumptive TB with the same kind of empathy as I would treat patients with other illness"*<br><br>"मैं अनुमानित टीबी से पीड़ित रोगियों के साथ उसी तरह की सहानुभूति के साथ व्यवहार करता हूँ जैसे मैं अन्य बीमारियों के रोगियों के साथ करता हूँ"
		2	Somewhat disagree<br><br>कुछ हद तक असहमत<br><br>થોડું બોવ અસંમત	
		3	Neither agree nor disagree<br><br>न सहमत और न ही असहमत<br><br>ન તો સંમત કે અસંમત	
		4	Somewhat agree<br><br>कुछ हद तक सहमत<br><br>થોડું બોવ સંમત	
		5	Strongly Agree<br><br>पूरी तरह से सहमत<br><br>ભારપૂર્વક સંમત	
2.2.3	I am confident that I can handle patients with TB	1	Strongly Disagree<br><br>पूरी तरह से असहमत	Read out the line to the respondent - *" I am confident that I can handle patients suffering from TB"*

	मुझे विश्वास है कि मैं टीबी के रोगियों को संभाल सकता हूँ<br><br>મને કોઈ વાંધો નથી કે, અન્ય લોકો જાણે છે કે હું ટીબીના દર્દીઓની સારવાર કરું છું		ભારપૂર્વક અસંમત	प्रतिवादी के लिए पंक्ति पढ़ें - "मुझे विश्वास है कि मैं टीबी से पीड़ित रोगियों को संभाल सकता हूँ"<br><br>Ask the respondent to what extent they agree or disagree with the statement.<br><br>प्रतिवादी से पूछें कि वे किस हद तक कथन से सहमत या असहमत हैं।
		2	Somewhat disagree<br><br>कुछ हद तक असहमत<br><br>થોડું બોવ અસંમત	
		3	Neither agree nor disagree<br><br>न सहमत और न ही असहमत<br><br>ન તો સંમત કે અસંમત	
		4	Somewhat agree<br><br>कुछ हद तक सहमत<br><br>થોડું બોવ સંમત	
		5	Strongly Agree<br><br>पूरी तरह से सहमत<br><br>ભારપૂર્વક સંમત	
2.3	*When I examine a presumptive TB patient (before confirming diagnosis)*<br><br>जब मैं एक संभावित टीबी रोगी की जांच करता हूं *(निदान की पुष्टि करने से पहले)*<br><br>જ્યારે હું ટીબીના સંભવિત દર્દીની તપાસ કરું છું *(નિદાનની પુષ્ટિ કરતા પહેલા)*			
				In the next set of questions you will ask the respondent what they are most likely to do if they find out that a person is suffering from presumptive TB.<br><br>प्रश्नों के अगले सेट में आप प्रतिवादी से पूछेंगे कि यदि उन्हें पता चलता है कि कोई व्यक्ति संभावित टीबी से पीड़ित है तो उनके क्या करने की सबसे अधिक जरुरत है।<br><br>You will have to probe separately for

<table>
<tr><td></td><td colspan="3"></td><td>each instance and ask them if they are willing to take the following steps.

आपको प्रत्येक उदाहरण के लिए अलग से जांच करनी होगी और उनसे पूछना होगा कि क्या वे निम्नलिखित कदम उठाने को तैयार हैं।</td></tr>
<tr><td rowspan="5">2.3.1</td><td rowspan="5">I refer to the nearest government facility

मैं निकटतम सरकारी सुविधा का उल्लेख करता हूं

હું નજીકની સરકારી સુવિધાનો સંદર્ભ આપું છું</td><td>1</td><td>Never

कभी नहीं

ક્યારેય નહીં</td><td rowspan="5">Please note that a Government Facility is a hospital or health service owned and managed by the Government.

कृपया ध्यान दें कि सरकारी सुविधा एक अस्पताल या स्वास्थ्य सेवा है जिसका स्वामित्व और प्रबंधन सरकार करती है।

In this question, ask the respondent if they would refer a patient to a nearest government facility if they find out that the person is suffering from presumptive TB.

इस प्रश्न में, प्रतिवादी से पूछें कि क्या वे किसी मरीज को निकटतम सरकारी सुविधा के लिए रेफर करेंगे यदि उन्हें पता चलता है कि वह व्यक्ति संभावित टीबी से पीड़ित है।

Please understand the following explanation of options below to know if the respondent is most likely willing to refer the patient to the nearest government facility if he or she is detected with TB.

कृपया यह जानने के लिए नीचे दिए गए विकल्पों के निम्नलिखित स्पष्टीकरण को समझें कि क्या प्रतिवादी रोगी को निकटतम सरकारी सुविधा में रेफर करने के लिए इच्छुक है यदि उसे टीबी का पता चला है।

1. Never- Please mark this option if the respondent says he/she</td></tr>
<tr><td>2</td><td>Rarely

शायद ही कभी

ભાગ્યે જ</td></tr>
<tr><td>3</td><td>Sometimes

कभी-कभी

ક્યારેક ક્યારેક</td></tr>
<tr><td>4</td><td>Often

अक्सर

ઘણીવાર</td></tr>
<tr><td>5</td><td>Frequently

बार-बार

વારંવાર</td></tr>
</table>

				never refers a patient to the government facility after getting detected with presumptive TB<br><br>कभी नहीं- कृपया इसे चिह्नित करें विकल्प यदि प्रतिवादी कहता है कि अनुमानित टीबी का पता चलने के बाद वह कभी भी किसी मरीज को सरकारी सुविधा के लिए रेफर नहीं करता है<br><br>2. **Rarely-** Please mark this if the respondent says he/she rarely refers a patient to a government facility after getting detected with presumptive TB.<br><br>शायद ही कभी- कृपया इसे चिह्नित करें यदि प्रतिवादी कहता है कि अनुमानित टीबी का पता चलने के बाद वह शायद ही कभी किसी मरीज को सरकारी सुविधा के लिए रेफर करता है।<br><br>3. **Sometimes-** You will mark this option only if the respondent says they only occasionally refer a person to a government facility after getting detected with presumptive TB.<br><br>कभी-कभी- आप चिह्नित करेंगे यह विकल्प केवल तभी होता है जब प्रतिवादी कहता है कि अनुमानित टीबी का पता चलने के बाद वे कभी-कभार ही किसी व्यक्ति को सरकारी सुविधा के लिए रेफर करते हैं।<br><br>4. **Often-** You will mark this option if the respondent says very often he/she refers the patient to a government facility when detected with presumptive TB.<br><br>अक्सर- आप इसे चिह्नित करेंगे विकल्प यदि प्रतिवादी कहता है कि वह रोगी को सरकारी सुविधा के लिए बहुत बार रेफर करता है, जब उसे संभावित टीबी का पता चलता है।

				5. **Frequently-** Please mark this option if the respondent says very frequently on a regular basis he/she refers a patient to a government facility if detected with presumptive TB.<br><br>बार-बार- कृपया चिह्नित करें यह विकल्प यदि प्रतिवादी नियमित आधार पर बहुत बार कहता है तो वह एक रोगी को सरकारी सुविधा के लिए संदर्भित करता है यदि उसे संभावित टीबी का पता चलता है।
2.3.2	I prescribe antibiotics (to rule out other conditions)<br><br>मैं एंटीबायोटिक्स लिखता हूँ (अन्य स्थितियों से इंकार करने के लिए)<br><br>હું અન્ય સ્થિતિઓને નકારી કાઢવા માટે એન્ટિબાયોટિક્સ લખું છું	1	Never<br><br>कभी नहीं<br><br>ક્યારેય નહીં	You need to understand **antibiotics** are medicines that are used for destroying bacteria and curing infections.<br><br>आपको यह समझने की जरूरत है कि एंटीबायोटिक्स दवाएं हैं जिनका उपयोग बैक्टीरिया को नष्ट करने और संक्रमण को ठीक करने के लिए किया जाता है<br><br>In this question, please ask the respondent how frequently he/she prescribes antibiotics to the patients after getting detected with presumptive TB.<br><br>इस प्रश्न में, कृपया प्रतिवादी से पूछें कि वह संभावित टीबी का पता चलने के बाद रोगियों को कितनी बार एंटीबायोटिक्स देता है।
		2	Rarely<br><br>शायद ही कभी<br><br>ભાગ્યે જ	
		3	Sometimes<br><br>कभी-कभी<br><br>ક્યારેક ક્યારેક	
		4	Often<br><br>अक्सर<br><br>ઘણીવાર	
		5	Frequently<br><br>बार-बार<br><br>વારંવાર	
2.3.3	I order diagnostic tests<br><br>मैं नैदानिक परीक्षणों का आदेश देता हूँ	1	Never<br><br>कभी नहीं	**Diagnostic tests** are tests that are conducted to diagnose a disease or condition in a person.

	હું ડાયગ્નોસ્ટિક ટેસ્ટસ કરાવું છું		ક્યારેય નહીં	नैदानिक परीक्षण ऐसे परीक्षण होते हैं जो किसी व्यक्ति में किसी बीमारी या स्थिति का जाँच करने के लिए किए जाते हैं।<br><br>Please ask the respondent how frequently they order the person to take diagnostic tests if they are detected with presumptive TB.<br><br>कृपया प्रतिवादी से पूछें कि यदि व्यक्ति संभावित टीबी के साथ पाए जाते हैं तो वे कितनी बार व्यक्ति को नैदानिक परीक्षण करने का आदेश देते हैं।
		2	Rarely<br><br>शायद ही कभी<br><br>ભાગ્યે જ	
		3	Sometimes<br><br>कभी-कभी<br><br>ક્યારેક ક્યારેક	
		4	Often<br><br>अक्सर<br><br>ઘણીવાર	
		5	Frequently<br><br>बार-बार<br><br>વારંવાર	
2.4	Which of the following tests do you frequently use for diagnosing pulmonary TB?<br><br>पल्मोनरी टीबी के निदान के लिए आप निम्नलिखित में से किस परीक्षण का अक्सर उपयोग करते हैं?<br><br>પલ્મોનરી ટીબીનું નિદાન કરવા માટે તમે નીચેનામાંથી કયા પરીક્ષણોનો વારંવાર ઉપયોગ કરો છો?	1	Chest X-Ray<br><br>छाती का एक्स - रे<br><br>છાતીનો એક્સ-રે	You need to understand the following terms before asking this question-<br><br>यह प्रश्न पूछने से पहले आपको निम्नलिखित शर्तों को समझना होगा-<br><br>Chest X-Ray: an imaging test used to examine the structures and organs in your chest. It helps diagnose symptoms such as-<br>● Breathing difficulties<br>● Bad or persistent cough<br>● Chest pain or injury<br><br>चेस्ट एक्स-रे एक इमेजिंग टेस्ट है जिसका उपयोग आपकी छाती में संरचनाओं और अंगों की जांच के लिए किया जाता है। यह लक्षणों का निदान करने में मदद करता है जैसे-<br><br>● सांस लेने में तकलीफ<br>● खराब या लगातार खांसी
		2	Sputum Smear microscopy<br><br>स्पुतम स्मीयर माइक्रोस्कोपी<br><br>સ્પુટમ સ્મીયર માઇક્રોસ્કોપી	
		3	Gene Xpert<br><br>जीन विशेषज्ञ<br><br>જીન એક્સ-પર્ટ	
		4	Sputum Culture	

			थूक संस्कृति<br><br>સ્પુટમ કલ્ચર	● सीने में दर्द या चोट<br><br>Sputum Smear microscopy: is a primary test conducted to confirm pulmonary tuberculosis. It helps in the rapid and reliable identification of pulmonary TB in patients. This can be used to diagnose TB in any part of the body.<br><br>स्पुतम स्मीयर माइक्रोस्कोपी: फुफ्फुसीय तपेदिक की पुष्टि के लिए आयोजित एक प्राथमिक परीक्षण है। यह रोगियों में फुफ्फुसीय टीबी की तीव्र और विश्वसनीय पहचान में मदद करता है। इसका उपयोग शरीर के किसी भी हिस्से में टीबी का निदान करने के लिए किया जा सकता है।<br><br>Gene Xpert: tested when sputum smear microscopy results are negative. This is considered a gold standard test to identify pulmonary TB.<br><br>जीन एक्सपर्ट: परीक्षण किया गया जब स्पुतम स्मीयर माइक्रोस्कोपी के परिणाम नकारात्मक होते हैं। फुफ्फुसीय टीबी की पहचान करने के लिए इसे स्वर्ण मानक परीक्षण माना जाता है।<br><br>Sputum Culture: checks for bacteria or lung infections by taking the patient's sample. This is also used to check which drugs respond to the individual to cure.<br><br>स्पुतम कल्चर: रोगी का नमूना लेकर बैक्टीरिया या फेफड़ों के संक्रमण की जांच करता है। इसका उपयोग यह जांचने के लिए भी किया जाता है कि कौन सी दवाएं व्यक्ति को ठीक करने के लिए प्रतिक्रिया करती हैं।<br><br>Blood test (CBC, ESR, etc): These are not confirmative but show the increased infection value in a
		5	Blood test (CBC, ESR, etc)<br><br>रक्त परीक्षण (सीबीसी, ईएसआर, आदि)<br><br>રક્ત પરીક્ષણો (સીબીસી, ઇએસઆર, વગેરે)	

				TB-diagnosed patient. CBC and ESR are identifications in a blood test.<br><br>रक्त परीक्षण (सीबीसी, ईएसआर, आदि): ये पुष्टिकारक नहीं हैं, लेकिन टीबी के निदान वाले रोगी में बढ़े हुए संक्रमण मूल्य को दर्शाते हैं। सीबीसी और ईएसआर रक्त परीक्षण में पहचान हैं।<br><br>Please ask the respondent which of the following tests they rely on for diagnosing pulmonary TB in a patient.<br><br>Mark the option accordingly as they answer.<br><br>कृपया प्रतिवादी से पूछें कि रोगी में फुफ्फुसीय टीबी के निदान के लिए वे निम्नलिखित में से किस परीक्षण पर भरोसा करते हैं।<br><br>उनके उत्तर के अनुसार विकल्प को चिह्नित करें।
2.5	*Please indicate how much you agree with each of the statements.*<br><br>कृपया बताएँ कि आप प्रत्येक कथन से किस हद तक सहमत हैं?<br><br>કૃપા કરીને સૂચવો કે તમે દરેક વિધાન સાથે કેટલા સહમત છો?			In the next set of questions, you will have to ask the respondent to what extent they agree or disagree with the statements.<br><br>प्रश्नों के अगले सेट में, आपको प्रतिवादी से पूछना होगा कि वे कथनों से किस हद तक सहमत या असहमत हैं।<br><br>You will have to probe separately for each respondent to understand their responses and mark accordingly.<br><br>आपको प्रत्येक प्रतिवादी के लिए उनकी प्रतिक्रियाओं को समझने और तदनुसार चिह्नित करने के लिए अलग से ध्यान करना होगा ।

2.5.1	I feel confident in my ability to collect relevant health-related information from patients<br><br>मुझे रोगियों से स्वास्थ्य संबंधी जानकारी एकत्र करने की अपनी क्षमता पर विश्वास है<br><br>દર્દીઓ પાસેથી આરોગ્ય સંબંધિત માહિતી એકત્રિત કરવાની મારી ક્ષમતામાં મને વિશ્વાસ છે	1	Strongly Disagree<br><br>पूरी तरह से असहमत<br><br>ભારપૂર્વક અસંમત	Please read out the statement to the respondent and ask her to what extent he/she agrees or disagrees with the statement-<br><br>कृपया प्रतिवादी को दिए गए कथन को पढ़ें और उससे पूछें कि वह किस हद तक इस कथन से सहमत या असहमत है-<br><br>*“ I I feel confident in my ability to collect relevant health-related information from patients”*<br><br>"मुझे रोगियों से स्वास्थ्य संबंधी प्रासंगिक जानकारी एकत्र करने की अपनी क्षमता पर विश्वास है"
		2	Somewhat disagree<br><br>कुछ हद तक असहमत<br><br>થોડું બોવ અસંમત	
		3	Neither agree nor disagree<br><br>न सहमत और न ही असहमत<br><br>ન તો સંમત કે અસંમત	
		4	Somewhat agree<br><br>कुछ हद तक सहमत<br><br>થોડું બોવ સંમત	Ask him/her the extent and mark accordingly.<br><br>उनसे पूछिए की वह किस हद तक सहमत या असहमत है और उसके अनुसार चुनिए।
		5	Strongly Agree<br><br>पूरी तरह से सहमत<br><br>ભારપૂર્વક સંમત	
2.5.2	I feel confident in my ability to collect relevant health-related information from patients<br><br>मुझे टीबी के रोग का पता लगाने हेतु आवश्यक जाँच करने के लिए अपने अनुभव पर भरोसा है | <br>ટીબી કેસ શોધવા માટે સંબંધિત ડાયગ્નોસ્ટિક ટેસ્ટનો ઉપયોગ કરવાની	1	Strongly Disagree<br><br>पूरी तरह से असहमत<br><br>ભારપૂર્વક અસંમત	You will also have to read out this question to the respondent and ask him or her to what extent they agree or disagree with the statement.<br><br>आपको प्रतिवादी को यह प्रश्न भी पढ़ना होगा और उससे पूछना होगा कि वे इस कथन से किस हद तक सहमत या असहमत हैं।<br><br>*“ I feel confident in my ability to collect relevant health-related information from patients”*
		2	Somewhat disagree<br><br>कुछ हद तक असहमत<br><br>થોડું બોવ અસંમત	
		3	Neither agree nor	

	મારી ક્ષમતામાં મને વિશ્વાસ છે		disagree<br><br>न सहमत और न ही असहमत<br><br>ન તો સંમત કે અસંમત	"मुझे रोगियों से स्वास्थ्य संबंधी प्रासंगिक जानकारी एकत्र करने की अपनी क्षमता पर विश्वास है"<br><br>Mark the option accordingly as he/she responds.<br><br>उनसे पूछिए और उसके अनुसार विकल्प चुनिए।
		4	Somewhat agree<br><br>कुछ हद तक सहमत<br><br>થોડું બોવ સંમત	
		5	Strongly Agree<br><br>पूरी तरह से सहमत<br><br>ભારપૂર્વક સંમત	
2.5.3	I feel confident in my ability to interpret an X-Ray film without an X-Ray report to detect a TB case<br><br>मुझे एक्स-रे रिपोर्ट के बिना एक्स-रे फिल्म देखकर टीबी का पता लगाने की अपनी क्षमता पर विश्वास है<br><br>મને ટીબી કેસ શોધવા માટે એક્સ-રે રિપોર્ટ વિના એક્સ-રે ફિલ્મનું અર્થઘટન કરવાની મારી ક્ષમતામાં વિશ્વાસ છે	1	Strongly Disagree<br><br>पूरी तरह से असहमत<br><br>ભારપૂર્વક અસંમત	Please understand that **X-Ray films** are photographic films used to make X-ray pictures.<br>कृपया समझें कि एक्स-रे फिल्में फोटोग्राफिक फिल्में हैं जिनका उपयोग एक्स-रे चित्र बनाने के लिए किया जाता है।<br><br>**X-Ray reports** are photographs or images obtained through the use of X-Rays.<br><br>एक्स-रे रिपोर्ट एक्स-रे के उपयोग के माध्यम से प्राप्त तस्वीरें या छवियां हैं।<br><br>Please ask the respondent to what extent he/she agrees or disagrees with this statement-<br><br>कृपया प्रतिवादी से पूछें कि वह इस कथन से किस हद तक सहमत या असहमत है-<br><br>" I feel confident enough in my ability to explain an X-Ray film without an X-Ray report to detect a TB case"
		2	Somewhat disagree<br><br>कुछ हद तक असहमत<br><br>થોડું બોવ અસંમત	
		3	Neither agree nor disagree<br><br>न सहमत और न ही असहमत<br><br>ન તો સંમત કે અસંમત	
		4	Somewhat agree<br><br>कुछ हद तक सहमत<br><br>થોડું બોવ સંમત	
		5	Strongly Agree	

			पूरी तरह से सहमत<br><br>ભારપૂર્વક સંમત	"मैं टीबी के मामले का पता लगाने के लिए एक्स-रे रिपोर्ट के बिना एक्स-रे फिल्म की व्याख्या करने की अपनी क्षमता में पर्याप्त आत्मविश्वास महसूस करता हूं"<br><br>Mark the answer accordingly as she responds.<br><br>उनसे पूछिए और उसके अनुसार विकल्प चुनिए।
2.5.4	I feel confident in my ability to read an X-Ray report to detect a TB case<br><br>मुझे टीबी के मामले का पता लगाने के लिए एक्स-रे रिपोर्ट पढ़ने की अपनी क्षमता पर भरोसा है<br><br>મને ટીબી કેસ શોધવા માટે એક્સ-રે રિપોર્ટ વાંચવાની મારી ક્ષમતામાં વિશ્વાસ છે	1	Strongly Disagree<br><br>पूरी तरह से असहमत<br><br>ભારપૂર્વક અસંમત	Similar to the previous question, please ask the respondent to what extent they agree or disagree with this statement-<br><br>पिछले प्रश्न के समान, कृपया प्रतिवादी से पूछें कि वे इस कथन से किस हद तक सहमत या असहमत हैं-<br><br>“I feel confident in my ability to read an X-Ray report to detect a TB case”<br><br>"मुझे टीबी के मामले का पता लगाने के लिए एक्स-रे रिपोर्ट पढ़ने की अपनी क्षमता पर भरोसा है"<br><br>Mark as they respond.<br><br>उनके जवाब के रूप में चिह्नित करें।
		2	Somewhat disagree<br><br>कुछ हद तक असहमत<br><br>થોડું બોવ અસંમત	
		3	Neither agree nor disagree<br><br>न सहमत और न ही असहमत<br><br>ન તો સંમત કે અસંમત	
		4	Somewhat agree<br><br>कुछ हद तक सहमत<br><br>થોડું બોવ સંમત	
		5	Strongly Agree<br><br>पूरी तरह से सहमत<br><br>ભારપૂર્વક સંમત	

2.5.5	I feel confident in my ability to deal with TB patients (if allowed to treat/medicate)<br><br>मुझे टीबी के रोगियों को संभालने की अपनी क्षमता पर भरोसा है (यदि इलाज/औषधि की अनुमति दी जाती है)<br><br>'ટીબી વિરોધી દવા લખવાની મારી ક્ષમતામાં મને વિશ્વાસ છે'.	1	Strongly Disagree<br><br>पूरी तरह से असहमत<br><br>ભારપૂર્વક અસંમત	In this question as well, read out the statement to the respondent and ask him/her to what extent he/she agrees or disagrees with the statement.<br><br>इस प्रश्न में भी, प्रतिवादी को दिए गए कथन को पढ़ें और उससे पूछें कि वह कथन से किस हद तक सहमत या असहमत है।<br><br>“I feel confident in my ability to deal with TB patients (if allowed to treat/medicate)”<br><br>"मुझे टीबी रोगियों से निपटने की अपनी क्षमता पर भरोसा है (यदि इलाज/औषधि की अनुमति दी जाती है)"
		2	Somewhat disagree<br><br>कुछ हद तक असहमत<br><br>થોડું બોવ અસંમત	
		3	Neither agree nor disagree<br><br>न सहमत और न ही असहमत<br><br>ન તો સંમત કે અસંમત	
		4	Somewhat agree<br><br>कुछ हद तक सहमत<br><br>થોડું બોવ સંમત	
		5	Strongly Agree<br><br>पूरी तरह से सहमत<br><br>ભારપૂર્વક સંમત	
2.6	How many new presumptive TB patients (with cough, fever, weight loss) do you see in a month?<br><br>आप एक महीने में कितने नए संभावित टीबी रोगी (खाँसी , बुखार, वजन घटाने के लक्षणों के साथ) देखते हैं?<br><br>મહિનામાં, તમે કેટલા નવા શંકાસ્પદ ટીબીના દર્દીઓ (ખાંસી, તાવ, વજન ઘટી જવું) જુઓ છો?	1	1-10<br><br>૧-૧૦	Our objective in this question is to know how many new presumptive TB patients the respondent attends in a month with symptoms such as cough, fever or weight loss.<br><br>इस प्रश्न में हमारा उद्देश्य यह जानना है कि प्रतिवादी एक महीने में कितने नए संभावित टीबी रोगियों में खांसी, बुखार या वजन घटने जैसे लक्षणों के साथ देखता है।<br><br>Please ask the respondent how many patients he/she sees in a month.
		2	11-20<br><br>૧૧-૨૦	
		3	21-30<br><br>૨૧-30	
		4	More than 30<br><br>30 से अधिक	

			૩૦ થી વધુ	कृपया प्रतिवादी से पूछें कि वह एक महीने में कितने रोगियों को देखता है।<br><br>Options are simple and straightforward.Mark accordingly.<br><br>विकल्प सरल और सीधे हैं। तदनुसार चिह्नित करें।
2.7	Have you referred a patient to the WHP TB program?<br><br>क्या आपने किसी मरीज को डब्ल्यूएचपी टीबी कार्यक्रम में रेफर किया है ?<br><br>શું તમે કોઈ દર્દીને ટીબી પ્રોગ્રામ/સરકારી આરોગ્ય સુવિધામાં મોકલ્યા છે?	1	Yes<br><br>हाँ<br><br>હા	Please understand that **WHP TB program** is the World health partners TB program.<br><br>Ask the respondent if they have referred any patient to a WHP TB program?<br><br>प्रतिवादी से पूछें कि क्या उन्होंने किसी मरीज को WHP TB प्रोग्राम के लिए रेफर किया है?<br><br>You will have to mark their response in 'Yes' or 'No'<br><br>आपको उनकी प्रतिक्रिया को 'हां' या 'नहीं' में चिह्नित करना होगा
		2	No<br><br>ना<br><br>ના	
2.7a	Please state why your response to the previous (2.7) question is 'No.'<br><br>कृपया कारण बताएं यदि पिछले प्रश्न (2.7) के लिए आपकी प्रतिक्रिया 'नहीं' है।<br><br>જો પાછલા (2.4) પ્રશ્નનો તમારો જવાબ 'ના' હોય તો કૃપા કરીને કારણ જણાવો	1	I don't treat TB patients.<br><br>मैं टीबी रोगियों का इलाज नहीं करता।<br><br>હું ટીબીના દર્દીઓની સારવાર કરતો નથી.	Note that this question will only open to respondents who have said that they do not refer a patient to the WHP TB program.<br><br>ध्यान दें कि यह प्रश्न केवल उन उत्तरदाताओं के लिए खुलेगा जिन्होंने कहा है कि वे किसी मरीज को WHP TB कार्यक्रम के लिए रेफर नहीं करते हैं।<br><br>Ask the respondent why he/she did not refer patients to the WHP TB program.<br><br>प्रतिवादी से पूछें कि उसने मरीजों को WHP TB कार्यक्रम के लिए क्यों नहीं रेफर किया।<br><br>Please refer to the explanation of
		2	The lab is not trustworthy.<br><br>प्रयोगशाला भरोसेमंद नहीं है।<br><br>લેબ ભરોસાપાત્ર	

			નથી.	options below to understand why they do not refer patients to the WHP TB program-<br><br>कृपया यह समझने के लिए नीचे दिए गए विकल्पों की व्याख्या देखें कि वे मरीजों को WHP TB कार्यक्रम के लिए क्यों नहीं रेफर करते हैं-<br><br>**1. I don’t treat TB patients-**<br>You will mark this if the respondent says they do not treat patients suffering from TB.<br><br>मैं टीबी के मरीजों का इलाज नहीं करता- आप इसे चिह्नित करेंगे यदि प्रतिवादी कहता है कि वे टीबी से पीड़ित रोगियों का इलाज नहीं करते हैं।<br><br>**2. The lab is not trustworthy-**<br>Please mark this if the respondent says they the labs are not reliable and as such they do not refer patients to the WHP TB program<br><br>लैब भरोसेमंद नहीं है- कृपया इसे चिह्नित करें यदि प्रतिवादी कहता है कि वे प्रयोगशालाएं विश्वसनीय नहीं हैं और इसलिए वे रोगियों को डब्ल्यूएचपी टीबी कार्यक्रम के लिए रेफर नहीं करते हैं<br><br>**3. I consider referring my patients to WHP TB program a time taking process-** Please mark this option if the respondent says referring patients to the WHP TV program is a time-taking process and as such they do not refer patients to WHP TB program.<br><br>मुझे लगता है कि मेरे मरीज़ो को WHP TB Program में रेफेर करना एक लम्बी प्रक्रिया है - कृपया इस विकल्प को चिह्नित करें यदि प्रतिवादी कहता है कि रोगियों को WHP टीवी कार्यक्रम में रेफर करना एक समय लेने वाली प्रक्रिया है और इसलिए वे
		3	I consider referring my patients to WHP TB program a time taking process.<br><br>मैं समय लेने वाली प्रक्रिया के रूप में अपने रोगियों को डब्ल्यूएचपी टीबी कार्यक्रम में भेजने पर विचार करता हूं।<br><br>હું મારા દર્દીઓને WHP TB પ્રોગ્રામમાં રેફર કરવાને સમય લેતી પ્રક્રિયા તરીકે માનું છું.	
		4	The WHP TB labs are far from the patient’s residence.<br><br>डब्ल्यूएचपी टीबी प्रयोगशालाएं मरीज के घर से बहुत दूर स्थित होती हैं।<br><br>ડબ્લ્યુએચપી ટીબી લેબ દર્દીના રહેઠાણથી દૂર સ્થિત છે.	
		5	The patients I referred to do not feel welcomed at the labs<br><br>जिन रोगियों को मैंने भेजा है वे प्रयोगशालाओं में स्वागत महसूस नहीं करते हैं ।<br><br>મેં જે દર્દીઓનો ઉલ્લેખ કર્યો છે તેઓ	

			લેબમાં આવકારદાયક નથી અનુભવતા	रोगियों को WHP TB प्रोग्राम के लिए रेफर नहीं करते हैं।<br><br>4. **The WHP TB labs are far from the patient's residence-** Please mark this option if the respondent says WHP TB labs are far from the patient's house and as such they do not refer the patients.<br><br>WHP टीबी लैब दूर हैं मरीज के घर से- कृपया इस विकल्प को चिह्नित करें यदि प्रतिवादी कहता है कि WHP TB प्रयोगशालाएं रोगी के घर से दूर हैं और इसलिए वे रोगियों को रेफर नहीं करती हैं।<br><br>5. **The patients I referred to do not feel welcomed at the labs**- You will mark this option if the respondent says that patients do not feel welcomed at the labs.<br><br>जिन रोगियों को मैंने प्रयोगशाला भेजा उन्हें वहां पर स्वागत महसूस नहीं हुआ- आप इस विकल्प को चिह्नित करेंगे यदि प्रतिवादी कहता है कि रोगी प्रयोगशालाओं में स्वागत महसूस नहीं करते हैं।<br><br>6. **The labs charge the patients an extra fee for a consultation-** Please mark this option if the respondent says that the patients are being charged an exorbitant fee for their consultation<br><br>प्रयोगशालाएं रोगियों को परामर्श के लिए एक अतिरिक्त शुल्क चार्ज करती हैं - कृपया इस विकल्प को चिह्नित करें यदि प्रतिवादी कहता है कि रोगियों से उनके परामर्श के लिए अत्यधिक शुल्क लिया जा रहा है<br><br>7. **Others-** Please mark this if the respondent says there is some other reason due to which they do not refer the patients to the WHP TB
		6	The labs charge the patients an extra fee for a consultation<br><br>प्रयोगशालाएं मरीजों से परामर्श के लिए अतिरिक्त शुल्क लेती हैं।<br><br>લેબ, દર્દીઓ પાસેથી પરામર્શ માટે વધારાની ફી વસૂલે છે.	
		-77	Others<br><br>अन्य<br><br>અન્ય	

				programs which have not been listed in the options above.<br><br>अन्य- कृपया इसे चिह्नित करें यदि प्रतिवादी का कहना है कि कुछ अन्य कारण हैं जिसके कारण वे रोगियों को डब्ल्यूएचपी टीबी कार्यक्रमों के लिए रेफर नहीं करते हैं जो कि उपरोक्त विकल्पों में सूचीबद्ध नहीं हैं।
2.7b	If others, please specify<br><br>यदि अन्य, तो कृपया स्पष्ट करें<br><br>જો અન્ય, તો કૃપા કરીને સ્પષ્ટ કરો			Note that this box will open if you have clicked on **'Others'** in the previous question.<br><br>ध्यान दें कि यदि आपने पिछले प्रश्न में 'अन्य' पर क्लिक किया है तो यह बॉक्स खुल जाएगा।<br><br>Please kindly ask the respondent to specify that 'other' reason why they do not refer the patients to the WHP TB program.<br><br>कृपया प्रतिवादी से यह बताने के लिए कहें कि 'अन्य' कारण वे मरीजों को डब्ल्यूएचपी टीबी कार्यक्रम के लिए क्यों नहीं रेफर करते हैं।
2.8	Do you use Chest X-Rays for diagnosing TB?<br><br>क्या आप टीबी के निदान के लिए चेस्ट एक्स-रे का उपयोग करते हैं?<br><br>શું તમે ટીબીના નિદાન માટે છાતીના એક્સ-રેનો ઉપયોગ કરો છો?	1	Yes<br><br>हाँ<br><br>હા	Our intent in this question is to understand if respondents rely on chest X-Rays for diagnosing TB.<br><br>इस प्रश्न में हमारा उद्देश्य यह समझना है कि क्या उत्तरदाता टीबी के निदान के लिए छाती के एक्स-रे पर भरोसा करते हैं।<br><br>Please ask the respondent if they rely on Chest X-Rays for detecting TB in patients.<br><br>कृपया प्रतिवादी से पूछें कि क्या वे रोगियों में टीबी का पता लगाने के लिए छाती के एक्स-रे पर निर्भर हैं।<br><br>Note that you will mark their response
		2	No<br><br>ना<br><br>ના	

				only in ‘Yes’ or ‘No’<br><br>ध्यान दें कि आप उनकी प्रतिक्रिया को केवल 'हां' या 'नहीं' में चिह्नित करेंगे।
2.8a	How do you use the Chest X-Rays?<br><br>आप चेस्ट एक्स-रे का उपयोग कैसे करते हैं?<br><br>તમે છાતીના એક્સ-રેનો ઉપયોગ કેવી રીતે કરો છો?	1	I examine X-Ray film<br><br>मैं एक्स-रे फिल्म की जांच करता हूं<br><br>હું એક્સ-રે ફિલ્મની તપાસ કરું છું	Please ask the respondent how they utilize the chest X-Rays from the patients.<br><br>कृपया प्रतिवादी से पूछें कि वे मरीजों के छाती के एक्स-रे का उपयोग कैसे करते हैं।<br><br>You will refer to the following explanation of options to understand how Chest X-Rays are used-<br><br>छाती के एक्स-रे का उपयोग कैसे किया जाता है, यह समझने के लिए आप विकल्पों की निम्नलिखित व्याख्या का उल्लेख करेंगे-<br><br>1. **I examine X-Ray film-**<br>Please mark this option if the respondent says he/she checks only the X-Ray film and doesn’t go through the report(we have already explained you before what is meant by an X-Ray film)<br><br>मैं एक्स-रे फिल्म की जांच करता हूं-<br>कृपया इस विकल्प को चिह्नित करें यदि प्रतिवादी कहता है कि वह केवल एक्स-रे फिल्म की जांच करता है और रिपोर्ट के माध्यम से नहीं जाता है (एक्स-रे फिल्म का क्या मतलब है, हमने आपको पहले ही समझाया है)<br><br>2. **I read the X-Ray report-**<br>You will mark this option if the respondent says he/she only relies on the X-Ray report that comes out and doesn’t go through the X-Ray film.<br><br>मैं एक्स-रे रिपोर्ट पढ़ता हूँ -<br>आप इस विकल्प को चिह्नित करेंगे यदि प्रतिवादी कहता है कि वह केवल एक्स-रे
		2	I read the X-Ray report<br><br>मैंने एक्स-रे रिपोर्ट पढ़ी<br><br>હું એક્સ-રે રિપોર્ટ વાંચું છુંહું એક્સ-રે ફિલ્મની તપાસ કરું છું અને રિપોર્ટ વાંચું છું	
		3	I examine the X-Ray film and read the report<br><br>मैं एक्स-रे फिल्म की जांच करता हूं और रिपोर्ट पढ़ता हूं<br><br>હું એક્સ-રે ફિલ્મની તપાસ કરું છું અને રિપોર્ટ વાંચું છું	

				रिपोर्ट पर निर्भर करता है जो बाहर आती है और एक्स-रे फिल्म के माध्यम से नहीं जाती है।<br><br>**3. I examine the X-Ray film and read the report-** You will mark this option if the respondent says he/she examines the X-Ray film and reads the report alongside.<br><br>मैं एक्स-रे फिल्म की जांच करता हूं और रिपोर्ट पढ़ता हूँ - आप इस विकल्प को चिह्नित करें यदि प्रतिवादी कहता है कि वह एक्स-रे फिल्म की जांच करता है और साथ में रिपोर्ट पढ़ता है।
2.8b	What do you look for in an X-Ray film or report?<br><br>आप एक्स-रे फिल्म या रिपोर्ट में क्या देखते हैं?<br><br>તમે એક્સ-રે ફિલ્મ અથવા રિપોર્ટમાં શું જુવો છો?	1	Infiltrates<br><br>इंफिल्ट्रेटस<br><br>ઘૂસણખોરી (ઇન્ફિલ્ટ્રાટેસ)	Please understand the following terms before you ask this question to the respondent-<br><br>प्रतिवादी से यह प्रश्न पूछने से पहले कृपया निम्नलिखित शर्तों को समझ लें-<br><br><br>Infiltrates: are opacities in the lungs<br>घुसपैठ: फेफड़ों में अस्पष्टता है<br>Cavities: empty spaces in the lungs<br>कैविटी: फेफड़ों में खाली जगह<br>Calcification (deposition of fibrous tissue): solidification of empty spaces in the lungs<br>कैल्सीफिकेशन (रेशेदार ऊतक का जमाव): फेफड़ों में खाली जगहों का जमना<br><br>Other specifications in the X-ray film<br><br>प्रतिवादी से यह प्रश्न पूछने से पहले कृपया निम्नलिखित शर्तों को समझ लें-<br><br><br>Ask the respondent what he/she looks for in the X-Ray film or X-Ray report after the diagnostic tests to confirm TB in a patient?<br><br>प्रतिवादी से पूछें कि वह रोगी में टीबी की
		2	Cavities<br><br>कैविटीज<br><br>પોલાણ (કેવિટીસ)	
		3	Calcification (deposition of fibrous tissue)<br><br>कैल्सीफिकेशन (रेशेदार ऊतक का जमाव)<br><br>કેલ્સિફિકેશન (તંતુમય પેશીઓનું નિરાકરણ) (ફાઇબ્રોસ તીસ્સુંએનું ડિપોઝિશન)	
		4	Other specifications in the X-ray film<br><br>एक्स-रे फिल्म में अन्य विनिर्देश	

			એક્સ-રે ફિલ્મમાં અન્ય વિશિષ્ટતાઓ	पुष्टि के लिए नैदानिक परीक्षण के बाद एक्स-रे फिल्म या एक्स-रे रिपोर्ट में क्या देखता है?<br><br>Mark the responses accordingly as they answer.<br><br>उत्तरों को वैसे ही चिह्नित करें जैसे वे उत्तर देते हैं।<br><br>**Other specifications in the X-ray film-** Please note that you will only be marking this option if the respondent says he/she looks for something else (other than **Infiltrates, Cavities or Calcification)**<br><br>कृपया ध्यान दें कि आप इस विकल्प को केवल तभी चिह्नित करेंगे जब प्रतिवादी कहता है कि वह कुछ और ढूंढ रहा है (घुसपैठ, गुहाओं या कैल्सीफिकेशन के अलावा)
2.8c	Please specify if your response to 2.8b is (4)<br><br>अगर 2.8b में आपने अन्य चुना है, तो कृपया निर्दिष्ट करें<br><br>શું તમારો પ્રતિસાદ 2.8b (4) હોય તો કૃપા કરીને સ્પષ્ટ કરો			This question will only open if you have clicked on the firth option in the previous question - Other specifications in the X-ray film<br><br>यह प्रश्न तभी खुलेगा जब आपने पिछले प्रश्न - एक्स-रे फिल्म में अन्य विशिष्टताओं के पहले विकल्प पर क्लिक किया हो<br><br>Ask the respondent to specify what is that other option which he/she looks for in the X-Ray film or X-Ray report while diagnosing a patient.<br><br>रोगी का निदान करते समय प्रतिवादी से यह बताने करने के लिए कहें कि एक्स-रे फिल्म या एक्स-रे रिपोर्ट में वह कौन सा अन्य विकल्प ढूंढ रहा है।
**Section 3: Artificial Intelligence for Diagnosing TB**<br><br>अनुभाग: **3** टीबी के निदान के लिए कृत्रिम बुद्धिमत्ता				

Note:1	**Artificial Intelligence for Diagnosing TB**<br><br>**Artificial Intelligence in Radiology:** Computer-Aided Detection algorithms such as Artificial Intelligence (AI) are increasingly used for detecting diseases, including TB. The World Health Organization has reviewed the use of chest x-ray as a screening tool for detecting TB in several populations. They have found that although a chest x-ray was not always accurate or sufficient to confirm a TB diagnosis, it played a critical role in early TB detection in children and adults.<br><br>The AI software indicates the chance that the patient has TB, which can help the radiologist make a better decision. In addition, when there is no radiologist available, the AI software can generate a report automatically. The lab can print the AI report and give it to the patient, who can bring it to the care provider. The following is an example of an AI report.<br><br>**AI-GENERATED RADIOLOGY REPORT (FOR AN ACTUAL PATIENT)**<br>**EXAM: X-RAY CHEST**<br>**CLINICAL HISTORY:**<br><br>**COMPARISON:**<br>None<br><br>**TECHNIQUE:**<br>Frontal projections of the chest were obtained<br><br>**FINDINGS:**<br>Without volume loss in the right upper zone.<br>Costophrenic angles appear normal.<br>The tracheal lucency is centrally placed.<br>The mediastinal and diaphragmatic outlines appear normal.<br>The heart shadow is normal.<br>The bony thoracic cage and soft tissues are normal.<br><br>**IMPRESSION:**<br>Without volume loss in the right upper zone, these findings are indicative of TB. Correlation with clinical findings, sputum examination and GeneXpert test is recommended.<br><br>**Date:**<br>08th Feb 2022<br><br>टीबी के निदान के लिए कृत्रिम बुद्धिमत्ता<br><br>रेडियोलॉजी में आर्टिफिशियल इंटेलिजेंस: टीबी सहित कई बीमारियों का पता लगाने के लिए आर्टिफिशियल इंटेलिजेंस (ए.आई) जैसे कंप्यूटर एडेड डिटेक्शन एल्गोरिदम का तेजी से उपयोग किया जा रहा है। विश्व स्वास्थ्य संगठन ने कुछ संख्या में टीबी का पता लगाने के लिए एक स्क्रीनिंग उपकरण के रूप में छाती के एक्स-रे के उपयोग की समीक्षा की है। उन्होंने पाया है कि टीबी के निदान की पुष्टि के लिए छाती का एक्स-रे हमेशा सटीक या पर्याप्त नहीं था, लेकिन इसने बच्चों और वयस्कों दोनों में टीबी का जल्द पता लगाने में महत्वपूर्ण भूमिका निभाई।

	ए.आई. सॉफ्टवेयर इस संभावना को इंगित करता है कि रोगी को टीबी हैजो रेडियोलॉजिस्ट को बेहतर निर्णय लेने में मदद कर सकता है। इसके अलावाजब कोई रेडियोलॉजिस्ट उपलब्ध नहीं होता हैतो ए.आई. सॉफ्टवेयर स्वचालित रूप से एक रिपोर्ट तैयार कर सकता है। लैब ए.आई. रिपोर्ट को प्रिंट कर मरीज को दे सकती हैजो इस रिपोर्ट को सेवा प्रदाता के पास ला सकता है।<br>निम्नलिखित ए.आई. रिपोर्ट का एक उदाहरण है।<br><br>ए.आई– उत्पन्नरेडियोलॉजी रिपोर्ट (एक वास्तविक रोगी के लिए)<br>परीक्षा: एक्स-रे चेस्ट<br><br>नैदानिक इतिहास:<br><br>तुलना:<br>कोई नहीं<br><br>तकनीक:<br>छाती के सामने सेअनुमान प्राप्त किए गए<br><br>जाँच - परिणाम:<br>दाएँ ऊपरी क्षेत्र में मात्रा हानि के बिना।<br>कोस्टोफ्रेनिक कोण सामान्य दिखाई देते हैं।<br>श्वासनली की ल्युसेंसी को केंद्र में रखा गया है।<br>मीडियास्टिनल और डायाफ्रामिक रूपरेखा सामान्य दिखाई देती है।<br>हृदय की छाया सामान्य है।<br>बोनी वक्ष पिंजरा और कोमल ऊतक सामान्य हैं।<br><br>प्रभाव:<br>दाहिने ऊपरी क्षेत्र में मात्रा में कमी के बिना, ये निष्कर्ष टीबी के संकेत हैं। नैदानिक निष्कर्षों, थूक परीक्षण और जेनएक्सपर्ट परीक्षण के साथ सहसंबंध की सिफारिश की जाती है।<br><br>तारीख:<br>08 फरवरी 2022<br><br>टीबीના નિદાન માટે આર્ટિફિશ્યલ ઇન્ટેલલીગેન્સ<br>રેડિયોલોજીમાં આર્ટિફિશિયલ ઇન્ટેલિજન્સ: આર્ટિફિશ્યલ ઇન્ટેલલીગેન્સ જેવા કમ્પ્યુટર-સહાયિત શોધ એલ્ગોરિધમનો ઉપયોગ ટીબી સહિતના રોગોને શોધવા માટે વધુને વધુ થાય છે. વર્લ્ડ હેલ્થ ઓર્ગેનાઈઝેશને ઘણી વસ્તીમાં ટીબી શોધવા માટે સ્ક્રીનીંગ સાધન તરીકે છાતીના એક્સ-રેના ઉપયોગની સમીક્ષા કરી છે. તેઓએ શોધ્યું છે કે છાતીનો એક્સ-રે ટીબીના નિદાનની પુષ્ટિ કરવા માટે હંમેશા પૂરતો નથી તે પરંતુ તે બાળકો અને પુખ્ત વયના લોકોમાં ટીબીની પ્રારંભિક તપાસમાં મહત્વપૂર્ણ ભૂમિકા ભજવે છે.<br><br>AI (આર્ટિફિશ્યલ ઇન્ટેલલીગેન્સ) સોફ્ટવેર દર્દીને ટીબી હોવાની સંભાવના દર્શાવે છે, જે રેડિયોલોજિસ્ટને વધુ સારો નિર્ણય લેવામાં મદદ કરી શકે છે. વધુમાં, જ્યારે કોઈ રેડિયોલોજિસ્ટ ઉપલબ્ધ ન હોય, ત્યારે AI (આર્ટિફિશ્યલ ઇન્ટેલલીગેન્સ) સોફ્ટવેર આપમેળે રિપોર્ટ જનરેટ કરી શકે છે. લેબ એઆઈ રિપોર્ટ પ્રિન્ટ કરી શકે છે અને દર્દીને આપી શકે છે, જે તેને હેલ્થ કેર પ્રોવાઇડરની પાસે લાવી શકે છે. નીચે AI (આર્ટિફિશ્યલ ઇન્ટેલલીગેન્સ) રિપોર્ટનું ઉદાહરણ છે.

<table>
<tr>
<td></td>
<td colspan="4">AI-જનરેટેડ રેડિયોલોજી રિપોર્ટ (એક વાસ્તવિક દર્દી માટે)
પરીક્ષા: એક્સ-રે ચેસ્ટ
ક્લિનિકલ હિસ્ટોરી:

સરખામણી:
કોઈ નહીં

ટેકનિક:
છાતીના આગળના અંદાજો મેળવવામાં આવ્યા હતા

તારણો:
જમણા ઉપલા ઝોનમાં વોલ્યુમ કોઈ નુકશાન નથી.
કોસ્ટોફ્રેનિક ખૂણા સામાન્ય દેખાય છે.
શ્વાસનળીની લ્યુસન્સી કેન્દ્રિય રીતે મૂકવામાં આવે છે.
મેડિયાસ્ટિનલ અને ડાયાફ્રેમેટિક રૂપરેખા સામાન્ય દેખાય છે.
હૃદયની છાયા સામાન્ય છે.
હાડકાના થોરાસિક કેજ અને સોફ્ટ તીસ્સુંએ સામાન્ય છે.

છાપ:
જમણા ઉપલા ઝોનમાં વોલ્યુમ નુકશાન નથી, તેથી આ તારણો ટીબી હોવાની સૂચના આપે છે. ક્લિનિકલ તારણો, સ્પુટમ પરીક્ષા અને જીનએક્સપર્ટ ટેસ્ટ સાથેના સંબંધ શોધવું અનિવાર્ય છે
Date:
08th Feb 2022</td>
</tr>
<tr>
<td>3.1</td>
<td colspan="3">Please indicate how much you agree with each of the statements.

कृपया बताएं कि आप प्रत्येक कथन से कितना सहमत हैं?

કૃપા કરીને સૂચવો કે તમે દરેક વિધાન સાથે કેટલા સહમત છો?</td>
<td>Similar to the previous questions, you will have to ask the respondent to what extent they agree or disagree with the statements.

पिछले प्रश्न की तरह , आपको प्रतिवादी से पूछना होगा कि वे कथनों से किस हद तक सहमत या असहमत हैं। .

Read out each statement to the respondent and ask him/her how much they agree or disagree with the statement.

प्रतिवादी को दिए गए प्रत्येक कथन को पढ़ें और उससे पूछें कि वे कथन से कितना सहमत या असहमत हैं।</td>
</tr>
<tr>
<td>3.1.1</td>
<td>I believe AI algorithms can reliably detect imaging findings that suggest TB

मेरा मानना है कि</td>
<td>1</td>
<td>Strongly Disagree

पूरी तरह से असहमत

ભારપૂર્વક અસંમત</td>
<td>You need to understand that Artificial intelligence (AI) or AI algorithms is an extended subset of machine learning that tells the computer how to learn to operate on its own.</td>
</tr>
</table>

	आर्टिफीसियल इंटेलीजेंस एल्गोरिदम सटीकता से इमेजिंग निष्कर्षों का पता लगाकर टीबी रोग के बारे सुझाव देते हैं।<br><br>હું માનું છું કે આર્ટિફિશિયલ ઇન્ટેલિજન્સ એલ્ગોરિધમ્સ ટીબી સૂચવતા ઇમેજિંગ તારણો વિશ્વસનીય રીતે શોધી શકે છે	2	Somewhat disagree<br><br>कुछ हद तक असहमत<br><br>થોડું બોવ અસંમત	आपको यह समझने की जरूरत है कि आर्टिफिशियल इंटेलिजेंस (एआई) या एआई एल्गोरिदम मशीन लर्निंग का एक विस्तारित उपसमुच्चय है जो कंप्यूटर को बताता है कि कैसे अपने आप काम करना सीखना है।<br><br>And, as such Artificial intelligence (AI) algorithms can be trained to recognise tuberculosis-related abnormalities on chest radiographs.<br><br>और, जैसे आर्टिफिशियल इंटेलिजेंस (एआई) एल्गोरिदम को छाती रेडियोग्राफ़ पर तपेदिक से संबंधित असामान्यताओं को पहचानने के लिए प्रशिक्षित किया जा सकता है.<br><br>Also please note that **Imaging Finding tests** are a type of test that makes detailed pictures of areas inside the body.<br><br>कृपया यह भी ध्यान दें कि इमेजिंग फाइंडिंग टेस्ट एक प्रकार का परीक्षण है जो शरीर के अंदर के क्षेत्रों की विस्तृत तस्वीरें बनाता है।<br><br>Please kindly read out this statement to the respondent and ask him/her to what extent he/she agrees or disagrees with the statement. *Note that 'I' here signifies the respondent's view.*<br><br>कृपया प्रतिवादी को यह कथन पढ़ें और उससे पूछें कि वह इस कथन से किस हद तक सहमत या असहमत है। ध्यान दें कि यहां 'मैं' प्रतिवादी के विचार को दर्शाता है।<br><br>"I believe AI algorithms can reliably detect detailed pictures of areas inside the body that can suggest TB in a patient"<br><br>"मेरा मानना है कि एआई एल्गोरिदम शरीर के अंदर के क्षेत्रों की विस्तृत तस्वीरों का
		3	Neither agree nor disagree<br><br>न सहमत और न ही असहमत<br><br>ન તો સંમત કે અસંમત	
		4	Somewhat agree<br><br>कुछ हद तक सहमत<br><br>થોડું બોવ સંમત	
		5	Strongly Agree<br><br>पूरी तरह से सहमत<br><br>ભારપૂર્વક સંમત	

				मज़बूती से पता लगा सकता है जो एक रोगी में टीबी का सुझाव दे सकते हैं"<br><br>Mark accordingly as he/she responds.<br><br>जैसा वह जवाब देता है, उसके अनुसार चिह्नित करें।
3.1.2	Overall, I believe the use of AI could improve the accuracy of the diagnosis of tuberculosis<br><br>कुल मिलाकर मेरा मानना है कि आर्टिफीसियल इंटेलीजेंस के प्रयोग द्वारा टीबी के उपचार की सटीकता में सुधार हो सकता है | <br>એકંદરે, હું માનું છું કે, આર્ટિફિશિયલ ઇન્ટેલિજન્સનો ઉપયોગ ટીબીની નિદાનની ચોકસાઈમાં સુધારો કરી શકે છે.	1	Strongly Disagree<br><br>पूरी तरह से असहमत<br><br>ભારપૂર્વક અસંમત	Our objective in this question is to understand to what extent the respondent thinks the use of AI can help in accurately diagnosing TB in patients.<br><br>इस प्रश्न में हमारा उद्देश्य यह समझना है कि प्रतिवादी को लगता है कि एआई का उपयोग रोगियों में टीबी का सटीक निदान करने में किस हद तक मदद कर सकता है।<br><br>Please read out the statement to the respondent and mark accordingly to what extent he/she agrees with the statement.<br><br>कृपया प्रतिवादी को दिए गए कथन को पढ़ें और तदनुसार चिह्नित करें कि वह कथन से किस हद तक सहमत है।
		2	Somewhat disagree<br><br>कुछ हद तक असहमत<br><br>થોડું બોવ અસંમત	
		3	Neither agree nor disagree<br><br>न सहमत और न ही असहमत<br><br>ન તો સંમત કે અસંમત	
		4	Somewhat agree<br><br>कुछ हद तक सहमत<br><br>થોડું બોવ સંમત	
		5	Strongly Agree<br><br>पूरी तरह से सहमत<br><br>ભારપૂર્વક સંમત	
3.2	Are you willing to try an AI software that could upload an X-ray image to generate	1	Yes<br><br>हाँ	Please understand that **Artificial intelligence or AI software** is a

	a report?<br><br>क्या आप एक ऐसा एआई सॉफ़्टवेयर आज़माने के इच्छुक हैं जो रिपोर्ट बनाने के लिए एक्स-रे छवि अपलोड कर सके?<br><br>શું તમે એઆઈ સોફ્ટવેર અજમાવવા માટે તૈયાર છો, જે રિપોર્ટ જનરેટ કરવા માટે એક્સ-રે ઈમેજ અપલોડ કરી શકે?		હા	computer application capable of intelligent behavior like learning, reasoning, and problem-solving. This software can perform a range of tasks that typically require human intelligence, from the simple job of timekeeping to the complex process of analyzing millions of data sets.<br><br>कृपया समझें कि आर्टिफिशियल इंटेलिजेंस या एआई सॉफ्टवेयर एक कंप्यूटर एप्लिकेशन है जो सीखने, तर्क करने और समस्या-समाधान जैसे बुद्धिमान व्यवहार करने में सक्षम है। यह सॉफ़्टवेयर कई प्रकार के कार्य कर सकता है, जिसमें आम तौर पर मानव बुद्धि की आवश्यकता होती है, टाइमकीपिंग के सरल कार्य से लेकर लाखों डेटा सेटों के विश्लेषण की जटिल प्रक्रिया तक।<br><br>Our intent in this question is to know if a respondent is interested in using an AI software that can generate an X-Ray report by uploading an X-Ray image?<br><br>इस प्रश्न में हमारा इरादा यह जानना है कि क्या कोई प्रतिवादी ऐसे एआई सॉफ़्टवेयर का उपयोग करने में रुचि रखता है जो एक्स-रे छवि अपलोड करके एक्स-रे रिपोर्ट तैयार कर सकता है?<br><br>Note that you have to only accept their response in 'Yes' or 'No'<br><br>ध्यान दें कि आपको उनकी प्रतिक्रिया केवल 'हां' या 'नहीं' में स्वीकार करनी होगी
		2	No<br><br>नहीं<br><br>ना	
Lab related questions<br><br>लैब संबंधित प्रश्न<br><br>લેબ સંબંધિત પ્રશ્નો				

3.3	What is the lab name to which you send patients as part of the WHP program?<br><br>उस लैब का नाम क्या है जिसमें आप मरीजों को डब्ल्यूएचपी प्रोग्राम के तहत भेजते हैं?<br><br>ડબ્લ્યુએચપી પ્રોગ્રામના ભાગ રૂપે તમે દર્દીઓને જે એક્સ-રે ફેસિલિટીમાં મોકલો છો તેનું નામ શું છે?			In this question, we want to know the name of the lab where the respondent sends patients under the WHP TB program.<br><br>इस प्रश्न में हम उस लैब का नाम जानना चाहते हैं जहां प्रतिवादी डब्ल्यूएचपी टीबी कार्यक्रम के तहत मरीजों को भेजता है।<br><br>Please ask the respondent the name of the lab where he/she sends patients who are a part of the WHP TB program.<br><br>कृपया प्रतिवादी से उस प्रयोगशाला का नाम पूछें जहां वह उन रोगियों को भेजता है जो डब्ल्यूएचपी टीबी कार्यक्रम का हिस्सा हैं।<br><br>Write the name of the lab in the section provided.<br><br>दिए गए सेक्शन में लैब का नाम लिखें।
3.4	How long have you been referring patients to this lab? (Even before the free X-Ray program of WHP)<br><br>आप कब से मरीजों को इस लैब में रेफर कर रहे हैं? (डब्ल्यूएचपी के मुफ्त एक्स-रे कार्यक्रम से पहले भी)<br><br>તમે દર્દીઓને કેટલા સમયથી આ લેબનો સંદર્ભ આપી રહ્યાં છો? (ડબ્લ્યુએચપીના મફત એક્સ-રે પ્રોગ્રામ પહેલા પણ)	1	Less than six months<br><br>छह महीने से कम<br><br>છ મહિનાથી ઓછા	Now that you know the name of the lab where the respondent refers his/her patients under the WHP program, please ask the respondent for how long they have been referring the patients to this particular lab.<br><br>अब जब आप उस प्रयोगशाला का नाम जानते हैं जहां प्रतिवादी अपने रोगियों को WHP कार्यक्रम के तहत संदर्भित करता है, तो कृपया प्रतिवादी से पूछें कि वे कितने समय से रोगियों को इस विशेष प्रयोगशाला में रेफर कर रहे हैं।<br><br>Please remind them that they can also include the period before the free X-Ray program started in WHP.<br><br>कृपया उन्हें याद दिलाएं कि वे WHP में निःशुल्क एक्स-रे कार्यक्रम शुरू होने से पहले की अवधि को भी शामिल कर सकते हैं।<br><br>Options for this question are simple.
		2	6 months-1 year<br><br>6 महीने-1 साल<br><br>૬ મહિના-૧ વર્ષ	
		3	1-3 years<br><br>1-3 वर्ष<br><br>૧-૩ વર્ષ	
		4	3-5 years<br><br>3-5 वर्ष<br><br>૩-૫ વર્ષ	
		5	More than 5 years	

			5 साल से अधिक<br><br>૫ વર્ષથી વધુ	इस प्रश्न के विकल्प सरल हैं।
3.5	How important are monetary benefits from labs?<br><br>प्रयोगशालाओं को जाँच रेफर करने के लिए आपको कितना कमीशन मिलता है ?<br><br>એક્સ-રે ફેસિલિટી તરફથી નાણાકીય લાભ કેટલો મહત્વપૂર્ણ છે?	1	I receive some monetary benefits, but they are nominal (less than 5% of my income)<br><br>मुझे कुछ मौद्रिक लाभ मिलते हैं, लेकिन वे नाममात्र हैं (मेरी आय का 5% से कम)<br><br>મને કેટલાક નાણાકીય લાભો મળે છે, પરંતુ તે નજીવા છે (મારી આવકના 5% કરતા ઓછા)	Please understand that **monetary benefits** are financial incentives paid to people for meeting certain goals.<br><br>कृपया समझें कि मौद्रिक लाभ कुछ लक्ष्यों को पूरा करने के लिए लोगों को दिए जाने वाले वित्तीय प्रोत्साहन हैं।<br><br>Ask the respondent how important are these monetary benefits that they receive from the labs.<br><br>प्रतिवादी से पूछें कि ये मौद्रिक लाभ कितने महत्वपूर्ण हैं जो उन्हें प्रयोगशालाओं से प्राप्त होते हैं।<br><br>Kindly refer to the following explanation of options to understand how important are these monetary benefits for the respondents-<br><br>1. **I receive some monetary benefits, but they are nominal (less than 5% of my income)-** You will mark this option if the respondent says he/she receives only a very small form of monetary benefits from these labs and this accounts to less than 5 % of the respondents income.<br>2. **I receive monetary benefits, and they are a small portion of my income (5-10% of my income)-** Please mark this option if the respondent says he/she receives a small portion of monetary benefits from the labs which is only 5-10% of their income.<br>3. **I receive monetary benefits, and they are a substantial portion of my income (more than 10% of my income)-** You will mark this option if the respondent says they
		2	I receive monetary benefits, and they are a small portion of my income (5-10% of my income)<br><br>मुझे मौद्रिक लाभ मिलते हैं, और वे मेरी आय का एक छोटा सा हिस्सा हैं (मेरी आय का 5-10%)<br><br>મને નાણાકીય લાભો પ્રાપ્ત થાય છે, અને તે મારી આવકનો એક નાનો હિસ્સો છે (મારી આવકના 5-10%)	
		3	I receive monetary benefits, and they are a substantial portion of my income (more than 10% of my income)	

			मुझे मौद्रिक लाभ मिलते हैं, और वे मेरी आय का एक बड़ा हिस्सा हैं (मेरी आय का 10% से अधिक)<br><br>મને નાણાકીય લાભો મળે છે, અને તે મારી આવકનો નોંધપાત્ર હિસ્સો છે (મારી આવકના 10% કરતાં વધુ)	receive quite a substantial share of monetary benefits which is almost 10% of his/her income.<br>**4. I do not receive any monetary benefits-** You will only mark this option if the respondent says he/she does not receive any monetary benefits.
		4	I do not receive any monetary benefits<br><br>मुझे कोई मौद्रिक लाभ नहीं मिलता है<br><br>મને કોઈ નાણાકીય લાભો પ્રાપ્ત થતા નથી થતા	
3.6	Do you receive monetary benefits for the X-Rays ordered through the free X-Ray voucher program?<br><br>क्या, एक्स-रे वाउचर प्रोग्राम के माध्यम से एक्स-रे के लिए ब्लूटूथ प्राप्त होते हैं?<br><br>શું તમે ફ્રી એક્સ-રે વાઉચર પ્રોગ્રામ દ્વારા ઓર્ડર કરાયેલા એક્સ-રે માટે નાણાકીય લાભો મેળવો છો?	1	Yes<br><br>हाँ<br><br>હા	In the previous question , we asked the respondents about receiving monetary benefits from labs but here , we want to know if they receive benefits for the X-Rays ordered through the free X-Ray program.<br><br>पिछले प्रश्न में, हमने उत्तरदाताओं से प्रयोगशालाओं से मौद्रिक लाभ प्राप्त करने के बारे में पूछा था, लेकिन यहां, हम जानना चाहते हैं कि क्या उन्हें मुफ्त एक्स-रे कार्यक्रम के माध्यम से ऑर्डर किए गए एक्स-रे के लिए लाभ मिलता है।<br><br>Ask if they receive monetary benefits for X-Rays ordered through the free X-Ray voucher program.<br><br>पूछें कि क्या उन्हें मुफ्त एक्स-रे वाउचर प्रोग्राम के माध्यम से ऑर्डर किए गए एक्स-रे के लिए मौद्रिक लाभ मिलते हैं।
		2	No<br><br>नहीं<br><br>ના	

				You will note their response only in 'Yes' or 'No'<br><br>आप उनकी प्रतिक्रिया केवल 'हां' या 'नहीं' में नोट करेंगे
3.7	Does the lab send radiology (X-Ray) reports?<br><br>क्या प्रयोगशाला रेडियोलॉजी (एक्स-रे) रिपोर्ट भेजती है?<br><br>શું રેડિયોલોજી ફેસિલિટી એક્સ-રે રિપોર્ટ મોકલે છે?	1	Yes, along with the X-Ray film, the lab provides a report<br><br>हाँ, एक्स-रे फिल्म के साथ, प्रयोगशाला एक रिपोर्ट प्रदान करती है<br><br>હા, એક્સ-રે ફિલ્મની સાથે, લેબ રિપોર્ટ પ્રદાન કરે છે	You need to understand that a **radiology report** is the official record of medical images that contains the interpretations and images.<br><br>आपको यह समझने की जरूरत है कि रेडियोलॉजी रिपोर्ट चिकित्सा छवियों का आधिकारिक रिकॉर्ड है जिसमें व्याख्याएं और छवियां शामिल हैं।<br><br>Please ask the respondent if the labs send them X-Ray radiology reports.<br><br>कृपया प्रतिवादी से पूछें कि क्या प्रयोगशालाएं उन्हें एक्स-रे रेडियोलॉजी रिपोर्ट भेजती हैं।<br><br>Refer to the explanation below to understand what kind of X-Ray radiology reports are sent by labs to the respondents-<br><br>प्रयोगशालाओं द्वारा उत्तरदाताओं को किस प्रकार की एक्स-रे रेडियोलॉजी रिपोर्ट भेजी जाती है, यह समझने के लिए नीचे दिए गए स्पष्टीकरण का संदर्भ लें-<br><br>1. **Yes, along with the X-Ray film, the lab provides a report-** Please mark this option if the respondent says the labs send them a X-Ray report along with X-Ray film,<br><br>हां, एक्स-रे फिल्म के साथ, लैब एक रिपोर्ट प्रदान करती है- कृपया इस विकल्प को चिह्नित करें यदि प्रतिवादी कहता है कि लैब उन्हें एक्स-रे फिल्म के साथ एक्स-रे रिपोर्ट भेजती है<br><br>2. **No, the lab only provides the X-Ray film-** Please mark this if the
		2	No, the lab only provides the X-Ray film<br><br>नहीं, लैब केवल एक्स-रे फिल्म प्रदान करती है<br><br>ના, લેબ ફક્ત એક્સ-રે ફિલ્મ જ પ્રદાન કરે છે	

				respondent says labs only send them the X-Ray film without the reports.<br><br>नहीं, लैब केवल एक्स-रे फिल्म प्रदान करती है- कृपया इसे चिह्नित करें यदि प्रतिवादी कहता है कि लैब केवल उन्हें रिपोर्ट के बिना एक्स-रे फिल्म भेजती है।
3.8	If you view radiology reports, please indicate your views on the following questions:<br><br>यदि आप रेडियोलॉजी रिपोर्ट देखते हैं, तो कृपया निम्नलिखित प्रश्नों पर अपने विचार बताएं:<br><br>જો તમે રેડિયોલોજી રિપોર્ટ્સ જુઓ છો, તો કૃપા કરીને નીચેના પ્રશ્નો પર તમારા મંતવ્યો સૂચવો:			Please note that in this next set of questions,we want to know the respondent's opinion on Radiology (X Ray) reports.<br><br>कृपया ध्यान दें कि प्रश्नों के इस अगले सेट में, हम रेडियोलॉजी (X Ray) रिपोर्ट पर प्रतिवादी की राय जानना चाहते हैं।<br><br>You will have to read out the following options to the respondent and ask him/her to what extent he/she agrees or disagrees with the statement-<br><br>आपको प्रतिवादी को निम्नलिखित विकल्पों को पढ़ना होगा और उससे पूछना होगा कि वह किस हद तक इस कथन से सहमत या असहमत है-
3.8.1	The radiology report often mentions important issues I would not have noticed myself<br><br>रेडियोलॉजी रिपोर्ट में बहुत बार उन जरूरी मुद्दों का उल्लेख किया जाता है जिन पर मैंने ख़ुद ध्यान नहीं दिया होता है ।<br><br>રેડિયોલોજીના અહેવાલોમાં ઘણીવાર મહત્વપૂર્ણ મુદ્દાઓનો ઉલ્લેખ કરવામાં આવે છે,જેના ઉપર મેં પોતે ધ્યાન નથી આપ્યું હોતું.	1	Strongly Disagree<br><br>पूरी तरह से असहमत<br><br>ભારપૂર્વક અસંમત	Please read out this statement to the respondent and ask if they agree with the statement that the radiology report often mentions important issues that he/she would not have discovered or noticed on their own.<br><br>कृपया प्रतिवादी को यह कथन पढ़ें और पूछें कि क्या वे इस कथन से सहमत हैं कि रेडियोलॉजी रिपोर्ट में अक्सर महत्वपूर्ण मुद्दों का उल्लेख होता है जिन्हें उन्होंने स्वयं खोजा या नोटिस नहीं किया होगा।<br><br>Ask to what extent they agree or disagree.<br><br>पूछें कि वे किस हद तक सहमत या असहमत हैं।
		2	Somewhat disagree<br><br>कुछ हद तक असहमत<br><br>થોડું બોવ અસંમત	
		3	Neither agree nor disagree<br><br>न सहमत और न ही असहमत<br><br>ન તો સંમત કે અસંમત	

		4	Somewhat agree<br><br>कुछ हद तक सहमत<br><br>થોડું બોવ સંમત	
		5	Strongly Agree<br><br>पूरी तरह से सहमत<br><br>ભારપૂર્વક સંમત	
3.8.2	The language and style of radiology reports are mostly clear<br><br>रेडियोलॉजी रिपोर्ट की भाषा और शैली ज्यादातर स्पष्ट होती है<br><br>રેડિયોલોજી રિપોર્ટ્સની ભાષા અને શૈલી મોટાભાગે સ્પષ્ટ છે	1	Strongly Disagree<br><br>पूरी तरह से असहमत<br><br>ભારપૂર્વક અસંમત	Ask the respondent if they think that the language and style of radiology (X-Ray) reports are simple and easy to understand.<br><br>प्रतिवादी से पूछें कि क्या उन्हें लगता है कि रेडियोलॉजी (एक्स-रे) रिपोर्ट की भाषा और शैली सरल और समझने में आसान है।
		2	Somewhat disagree<br><br>कुछ हद तक असहमत<br><br>થોડું બોવ અસંમત	
		3	Neither agree nor disagree<br><br>न सहमत और न ही असहमत<br><br>ન તો સંમત કે અસંમત	
		4	Somewhat agree<br><br>कुछ हद तक सहमत<br><br>થોડું બોવ સંમત	
		5	Strongly Agree<br><br>पूरी तरह से सहमत<br><br>ભારપૂર્વક સંમત	
3.8.3	The radiologist who reads the X-rays and writes the report is competent	1	Strongly Disagree<br><br>पूरी तरह से असहमत	Please understand that a **radiologist** is a specialist doctor who uses x-ray, MRI, ultrasound to diagnose and treat

	जो रेडियोलॉजिस्ट एक्स-रे पढ़ता है और रिपोर्ट लिखता है वह अच्छा होता है ।<br><br>જે રેડીયોલોજીસ્ટ એક્સરે વાંચે છે અને રિપોર્ટ લખે છે તે સારો હોય છે		ભારપૂર્વક અસંમત	human disease or injury.<br><br>कृपया समझें कि रेडियोलॉजिस्ट एक विशेषज्ञ डॉक्टर है जो मानव रोग या चोट के निदान और उपचार के लिए एक्स-रे, एमआरआई, अल्ट्रासाउंड का उपयोग करता है।<br><br>Ask the respondent if he thinks that the radiologist who reads and writes the X-Ray report is efficient and qualified enough.<br><br>प्रतिवादी से पूछें कि क्या उसे लगता है कि रेडियोलॉजिस्ट जो एक्स-रे रिपोर्ट पढ़ता और लिखता है, वह कुशल और योग्य है।
		2	Somewhat disagree<br><br>कुछ हद तक असहमत<br><br>થોડું બોવ અસંમત	
		3	Neither agree nor disagree<br><br>न सहमत और न ही असहमत<br><br>ન તો સંમત કે અસંમત	
		4	Somewhat agree<br><br>कुछ हद तक सहमत<br><br>થોડું બોવ સંમત	
		5	Strongly Agree<br><br>पूरी तरह से सहमत<br><br>ભારપૂર્વક સંમત	
3.8.4	I am generally satisfied with the X-ray reports I receive from the radiologist<br><br>मैं आमतौर पर रेडियोलॉजिस्ट से प्राप्त एक्स-रे रिपोर्ट से संतुष्ट होता हूँ<br><br>હું સામાન્ય રીતે રેડિયોલોજિસ્ટ પાસેથી મેળવેલા એક્સ-રે રિપોર્ટ્સથી સંતુષ્ટ છું	1	Strongly Disagree<br><br>पूरी तरह से असहमत<br><br>ભારપૂર્વક અસંમત	Please read out this statement to the respondent and ask him/her to what he/she disagrees with the statement-<br><br>कृपया प्रतिवादी को यह कथन पढ़ें और उससे पूछें कि वह किस कथन से असहमत है-<br><br>" I am generally satisfied with the X-ray reports I receive from the radiologist"<br><br>"मैं आमतौर पर रेडियोलॉजिस्ट से प्राप्त एक्स-रे रिपोर्ट से संतुष्ट होता हूँ"
		2	Somewhat disagree<br><br>कुछ हद तक असहमत<br><br>થોડું બોવ અસંમત	
		3	Neither agree nor disagree<br><br>न सहमत और न ही असहमत	

			ન તો સંમત કે અસંમત	
		4	Somewhat agree<br><br>कुछ हद तक सहमत<br><br>થોડું બોવ સંમત	
		5	Strongly Agree<br><br>पूरी तरह से सहमत<br><br>ભારપૂર્વક સંમત	
**Section 4: Free CXR program questions**<br><br>अनुभाग **4:** मुफ़्त सीएक्सआर कार्यक्रम के प्रश्न<br><br>વિભાગ **4:** મફત સીએક્સઆર કાર્યક્રમના પ્રશ્નો				
4.1	Do patients usually visit chemists or healthcare providers for a persistent cough before coming to your clinic?<br><br>क्या मरीज आपके क्लिनिक में आने से पहले लगातार खांसी के लिए केमिस्ट या स्वास्थ्य सेवा प्रदाताओं के पास जाते हैं?<br><br>તમારા ક્લિનિકમાં આવતા પહેલા દર્દીઓ સામાન્ય રીતે સતત ઉધરસ માટે કયા કેમિસ્ટ અથવા હેલ્થ કેર પ્રોવાઇડરની મુલાકાત લે છે ?	1	Yes<br><br>हाँ<br><br>હા	Please understand that A **health care provider** is an individual health professional who is licensed to provide health care diagnosis and treatment services including medication, surgery and medical devices.<br><br>कृपया समझें कि एक स्वास्थ्य देखभाल प्रदाता एक व्यक्तिगत स्वास्थ्य पेशेवर है जिसे दवा, सर्जरी और चिकित्सा उपकरणों सहित स्वास्थ्य देखभाल निदान और उपचार सेवाएं प्रदान करने के लिए लाइसेंस प्राप्त है।<br><br>And, a **chemist** is a person who sells medicines.<br><br>और, एक केमिस्ट वह व्यक्ति होता है जो दवाएँ बेचता है।<br><br>Ask the respondent if the patients visit chemists or healthcare providers for a consistent and a continuous cough before coming to the clinic.<br><br>प्रतिवादी से पूछें कि क्या रोगी क्लिनिक में आने से पहले लगातार खांसी के लिए
		2	No<br><br>नहीं<br><br>ના	

				केमिस्ट या स्वास्थ्य सेवा प्रदाताओं के पास जाते हैं।<br><br>Note their response only in 'Yes' or 'No'<br><br>उनकी प्रतिक्रिया केवल 'हां' या 'नहीं' में नोट करें
4.2	Which chemists or healthcare providers do patients usually visit for a persistent cough before coming to your clinic?<br><br>आपके क्लिनिक में आने से पहले रोगी आमतौर पर लगातार खांसी के लिए किन केमिस्ट या स्वास्थ्य सेवा प्रदाताओं के पास जाते हैं?<br><br>તમારા ક્લિનિકમાં આવતા પહેલા સતત ઉધરસ માટે દર્દીઓ સામાન્ય રીતે કયા કેમિસ્ટ અથવા હેલ્થ કેર પ્રોવાઇડરની મુલાકાત લે છે?			This question aims at understanding which chemists or health care providers do the patients visit for a continuous cough.<br><br>इस प्रश्न का उद्देश्य यह समझना है कि रोगी लगातार खांसी के लिए कौन से रसायनज्ञ या स्वास्थ्य देखभाल प्रदाता के पास जाते हैं<br><br>Please refer to the previous question to understand the meaning of a health care provider and chemists in detail.<br><br>स्वास्थ्य देखभाल प्रदाता और रसायनज्ञों के अर्थ को विस्तार से समझने के लिए कृपया पिछला प्रश्न देखें।<br><br>Accordingly please note down the name.<br>तदनुसार कृपया नाम नोट कर लें।<br><br>If the patient doesn't visit any clinic, write 'None'.<br><br>यदि रोगी किसी क्लिनिक में नहीं जाता है तो 'कोई नहीं' लिखें।
4.3	Were you ordering a CXR for patients with persistent cough or breathing issues before the free CXR program?<br><br>क्या आप मुफ्त सीएक्सआर	1	Yes<br><br>हाँ<br><br>હા	Please understand that a **CXR** is a Chest X-Ray which is an imaging test used to examine the structures and organs in your chest. It helps diagnose symptoms such as-<br>• Breathing difficulties<br>• Bad or persistent cough
		2	No	

	कार्यक्रम से पहले लगातार खांसी या सांस लेने में समस्या वाले रोगियों के लिए सीएक्सआर का आदेश दे रहे थे?<br><br>શું તમે મફત CXR પ્રોગ્રામ પહેલાં સતત ઉધરસ અથવા શ્વાસની સમસ્યા ધરાવતા દર્દીઓ માટે CXR ઑર્ડર કરી રહ્યાં હતા?		नहीं<br><br>ના	• Chest pain or injury<br><br>चेस्ट एक्स-रे एक इमेजिंग टेस्ट है जिसका उपयोग आपकी छाती में संरचनाओं और अंगों की जांच के लिए किया जाता है। यह लक्षणों का निदान करने में मदद करता है जैसे-<br><br>• सांस लेने में तकलीफ<br>• खराब या लगातार खांसी<br>• सीने में दर्द या चोट<br><br>कृपया समझें कि सीएक्सआर चेस्ट एक्स-रे है।<br><br>In this question, we want to know if the respondent usually orders the patient to get a CXR who were suffering from consistent cough and breathing issues even before the CXR program was introduced?<br><br>इस प्रश्न में, हम जानना चाहते हैं कि क्या प्रतिवादी आमतौर पर रोगी को सीएक्सआर प्राप्त करने का आदेश देता है जो सीएक्सआर कार्यक्रम शुरू होने से पहले ही लगातार खांसी और सांस लेने में समस्या से पीड़ित थे?<br><br>Please ask the respondent if he/she used to prescribe CXR to patients suffering from persistent cough or breathing issues even before the free CXR program was introduced.<br><br>कृपया प्रतिवादी से पूछें कि क्या मुफ्त सीएक्सआर कार्यक्रम शुरू होने से पहले ही वह लगातार खांसी या सांस लेने की समस्या से पीड़ित रोगियों को सीएक्सआर लिखता था।<br><br>Note their response only in 'Yes' or 'No'<br><br>उनकी प्रतिक्रिया केवल 'हां' या 'नहीं' में नोट करें

<table>
<tr><td>4.4</td><td colspan="3">Please respond if any of your following practices have changed since the free CXR program was introduced:

कृपया जवाब दें कि क्या नि:शुल्क सीएक्सआर कार्यक्रम शुरू होने के बाद आपकी निम्नलिखित में से कोई भी प्रथा बदल गई है:

મફત CXR પ્રોગ્રામ રજૂ થયા પછી તમારી નીચે આપેલી કોઈપણ પ્રેક્ટિસ બદલાઈ હોય તો કૃપા કરીને પ્રતિસાદ આપો :</td><td>In the next set of questions, we would like to know about the changes that have taken place after the free CXR program was introduced as a part of the WHP program.

प्रश्नों के अगले सेट में, हम उन परिवर्तनों के बारे में जानना चाहेंगे जो डब्ल्यूएचपी कार्यक्रम के एक भाग के रूप में मुफ्त सीएक्सआर कार्यक्रम पेश किए जाने के बाद हुए हैं।</td></tr>
<tr><td rowspan="4">4.4.1</td><td rowspan="4">Number of patients (all types) seen per month

प्रति माह देखे गए रोगियों (सभी प्रकार) की संख्या

મહિને જોવા મળતા (બધા પ્રકારના) દર્દીઓની સંખ્યા</td><td>1</td><td>Stayed the same

वही रहा

સમાન છે</td><td rowspan="4">Our objective in this question is to know if there has been a change in the number of patients that the respondent has attended after the free CXR program was introduced.

इस प्रश्न में हमारा उद्देश्य यह जानना है कि क्या मुफ्त सीएक्सआर कार्यक्रम शुरू होने के बाद प्रतिवादी ने भाग लेने वाले रोगियों की संख्या में कोई बदलाव किया है।

Ask the respondent if the number of patients he/she has attended have changed after the free CXR program was introduced.

प्रतिवादी से पूछें कि क्या मुफ्त सीएक्सआर कार्यक्रम शुरू होने के बाद उसने जिन रोगियों में भाग लिया है, उनकी संख्या बदल गई है।

You will refer to the following options to understand which option to mark accordingly-

यह समझने के लिए कि किस विकल्प को तदनुसार चिह्नित करना है, आप निम्नलिखित विकल्पों का उल्लेख करेंगे-

1. Stayed the same- You will mark this option if the respondent says there has been no change in the number of patients that the patient has attended after the free CXR program was introduced.</td></tr>
<tr><td>2</td><td>Increased

बढ़ा हुआ

વધારો થયો છે</td></tr>
<tr><td>3</td><td>Decreased

घटा हुआ

ઘટાડો થયો છે</td></tr>
<tr><td>4</td><td>N/A</td></tr>
</table>

				वही रहा- आप इस विकल्प को चिह्नित करेंगे यदि प्रतिवादी कहता है कि मुफ्त सीएक्सआर कार्यक्रम शुरू होने के बाद रोगी ने जिन रोगियों में भाग लिया है, उनकी संख्या में कोई बदलाव नहीं आया है।<br><br>2. **Increased-** Please mark this option if the respondent says the number of patients have increased ever since the new CXR program was introduced.<br>बढ़ा हुआ - कृपया इस विकल्प को चिह्नित करें यदि प्रतिवादी कहता है कि नए सीएक्सआर कार्यक्रम की शुरुआत के बाद से रोगियों की संख्या में वृद्धि हुई है।<br><br>3. **Decreased-** Please mark this if the respondent says that the number of patients have decreased after the free CXR program was introduced.<br><br>घटी हुई - कृपया इसे चिह्नित करें यदि प्रतिवादी कहता है कि मुफ्त सीएक्सआर कार्यक्रम शुरू होने के बाद रोगियों की संख्या में कमी आई है।<br><br>4. **N/A-** You will only mark this option if the respondent doesn’t want to say anything or refuses to say anything in this regard.<br><br>एन/ए- आप इस विकल्प को केवल तभी चिह्नित करेंगे जब प्रतिवादी कुछ कहना नहीं चाहता या इस संबंध में कुछ भी कहने से इंकार कर देता है।
4.4.2	Has the free CXR program impacted your practices in any other way?<br><br>क्या मुफ़्त सीएक्सआर कार्यक्रम ने आपके अभ्यासों को किसी अन्य तरीके से प्रभावित किया है?<br><br>શું ફ્રી CXR પ્રોગ્રામે તમારી		Please specify<br><br>कृपया निर्दिष्ट करें	Our objective in this question is to know how the free CXR program that was introduced has impacted the respondent’s work.<br><br>इस प्रश्न में हमारा उद्देश्य यह जानना है कि शुरू किए गए मुफ्त सीएक्सआर कार्यक्रम ने प्रतिवादी के काम को कैसे प्रभावित किया है।

	પ્રેક્ટિસને અન્ય કોઈ રીતે અસર કરી છે?			Please ask the respondent how the free CXR program has impacted the respondent's clinical practices in any way.<br><br>कृपया प्रतिवादी से पूछें कि मुफ्त सीएक्सआर कार्यक्रम ने किसी भी तरह से प्रतिवादी के नैदानिक अभ्यासों को कैसे प्रभावित किया है।<br><br>Write the answer in the section provided below.<br><br>उत्तर नीचे दिए गए अनुभाग में लिखें।
**Section 5:** ***Demographics***<br><br>अनुभाग **5:** जनसांख्यिकीय<br><br>વિભાગ **5:** વસ્તી વિષયક (ડેમોગ્રાફી)				
5.1	Clinic ID from WHP System<br><br>WHP सिस्टम से क्लिनिक आईडी<br><br>ડબ્લ્યુએચપી સિસ્ટમમાંથી ક્લિનિક ID			The enumerator would be provided with a list containing the clinic ids.<br><br>इन्यूमरेटर को क्लिनिक आईडी वाली एक सूची प्रदान की जाएगी।<br><br>Please write the Clinic ID you have been provided with from the WHP system.<br><br>कृपया वह क्लिनिक आईडी लिखें जो आपको WHP प्रणाली से प्रदान की गई है।
5.2	The name of the clinic<br><br>क्लिनिक का नाम<br><br>ક્લિનિકનું નામ			Here, you will have to write the name of the clinic where you are conducting the interview.<br><br>यहां आपको उस क्लिनिक का नाम लिखना होगा जहां आप इंटरव्यू आयोजित कर रहे हैं।
5.3	Address of the clinic<br><br>क्लिनिक का पता<br><br>ક્લિનિકનું સરનામું			Please write the address of the clinic where the respondent is practicing at. This will be the same place where you are conducting the interview at.

				कृपया उस क्लिनिक का पता लिखें जहां प्रतिवादी अभ्यास कर रहा है।<br>यह वही स्थान होगा जहां आप साक्षात्कार आयोजित कर रहे हैं।<br><br>In case you do not know the address of that particular clinic , please ask the respondent for assistance.<br><br>यदि आप उस विशेष क्लिनिक का पता नहीं जानते हैं, तो कृपया प्रतिवादी से सहायता मांगें।
5.4	Mobile Number<br><br>मोबाइल नं.<br><br>મોબાઇલ નંબર			Ask the respondent for their mobile number<br><br>प्रतिवादी से उनका मोबाइल नंबर मांगें<br><br>Make sure it's a 10-digit number<br><br>सुनिश्चित करें कि यह 10 अंकों की संख्या है<br><br>In case the respondent refuses to share his number then enter 9999999999<br><br>यदि प्रतिवादी अपना नंबर साझा करने से इनकार करता है तो 9999999999 . दर्ज करें
5.5	Name of Health Care Provider<br><br>स्वास्थ्य देखभाल प्रदाता का नाम<br><br>આરોગ્ય સંભાળ કાર્યકર્તા નુ નામ			In this question, we want to know the name of the healthcare provider i.e the respondent.<br><br>इस प्रश्न में, हम स्वास्थ्य सेवा प्रदाता यानी प्रतिवादी का नाम जानना चाहते हैं।<br><br>Please politely ask the respondent his/her name.<br><br>कृपया प्रतिवादी से विनम्रतापूर्वक उसका नाम पूछें।
5.5a	Type of provider<br><br>प्रदाता का प्रकार	1	MD (Doctor of Medicine) Ayurveda	This question aims to know the kind of a healthcare provider the respondent is.

	પ્રદાતા (હેલ્થ પ્રોવાઈડર) ના પ્રકાર		एम.डी. आयुर्वेद<br><br>એમડી(ડોક્ટર ઓફ મેડિસિન) આયુર્વેદ	इस प्रश्न का उद्देश्य यह जानना है कि प्रतिवादी किस प्रकार का स्वास्थ्य सेवा प्रदाता है।<br><br>You will have to ask the respondent what kind of a healthcare provider he/she. Please read out the options to the respondent to know their designation and profile as a healthcare provider.<br><br>आपको प्रतिवादी से पूछना होगा कि वह किस प्रकार का स्वास्थ्य सेवा प्रदाता है। कृपया प्रतिवादी के लिए एक स्वास्थ्य सेवा प्रदाता के रूप में उनका पदनाम और प्रोफ़ाइल जानने के लिए विकल्पों को पढ़ें।<br><br>Please refer to the explanation below to better understand a few of the options:<br><br>कुछ विकल्पों को बेहतर ढंग से समझने के लिए कृपया नीचे दी गई व्याख्या देखें:<br><br>**Diploma in alternative medical system (DAMS)-** Please understand that Diploma in alternative medicine could mean Diploma in Yoga. Diploma in Physiotherapy.Ask the respondent if they have done a Diploma in Alternative medical system.<br><br>वैकल्पिक चिकित्सा प्रणाली में डिप्लोमा (डीएएमएस) - कृपया समझें कि वैकल्पिक चिकित्सा में डिप्लोमा का अर्थ योग में डिप्लोमा हो सकता है। फिजियोथेरेपी में डिप्लोमा। प्रतिवादी से पूछें कि क्या उन्होंने वैकल्पिक चिकित्सा प्रणाली में डिप्लोमा किया है।<br><br>**Intermediate-** Please note that Intermediate is an academic diploma awarded after the completion of 12th grade. Please ask the respondent if they have done any intermediate
		2	MD (Doctor of Medicine) Homeopathy<br><br>एम.डी. होम्योपैथी<br><br>એમડી (ડોક્ટર ઓફ મેડિસિન) હોમિયોપેથી	
		3	Bachelor of Ayurveda, Medicine and Surgery (BAMS)<br><br>बी.ए.एम.एस.<br><br>આયુર્વેદ, મેડિસિન એન્ડ સર્જરીમાં સ્નાતક (બીએએમએસ)	
		4	Bachelor in Homeopathic Medicine and surgery (BHMS)<br><br>बी.एच.एम. एस<br><br>હોમિયોપેથિક મેડિસિન અને સર્જરીમાં સ્નાતક (બીએચએમએસ)	
		5	Bachelor of Eastern Medicine and Surgery (BEMS)<br><br>बी.यु.एम.एस<br><br>પૂર્વીય દવા અને સર્જરીમાં સ્નાતક (બીઈએમએસ)	

		6	Diploma in alternative medical system (DAMS)<br><br>डी.ए.एम.एस<br><br>વૈકલ્પિક મેડિકલ સિસ્ટમમાં ડિપ્લોમા (ડીએએમએસ)	degree.<br><br>इंटरमीडिएट- कृपया ध्यान दें कि इंटरमीडिएट एक अकादमिक डिप्लोमा है जो 12 वीं कक्षा के पूरा होने के बाद प्रदान किया जाता है। कृपया प्रतिवादी से पूछें कि क्या उन्होंने कोई इंटरमीडिएट डिग्री की है।
		7	Diploma in Homeopathic Medicine and Surgery (DHMS)<br><br>डी.एच.एम.एस.<br><br>હોમિયોપેથિક મેડિસિન એન્ડ સર્જરીમાં ડિપ્લોમા (ડીએચએમએસ)	
		8	M.Pharm (Master of Pharmacy<br><br>एम. फार्मा<br><br>એમ.ફાર્મ (માસ્ટર ઑફ ફાર્મસી)	
		9	B.Pharm (Bachelor of Pharmacy)<br><br>बी. फार्मा<br><br>.બી.ફાર્મ (ફાર્મસીમાં સ્નાતક)	
		10	D.Pharm (Diploma in Pharmacy)<br><br>डी. फार्मा<br><br>ડી.ફાર્મ (ફાર્મસીમાં ડિપ્લોમા)	
		11	Post-Graduation	

			स्नातकोत्तर<br><br>અનુસ્નાતક	
		12	Graduation<br><br>स्नातक<br><br>સ્નાતક	
		13	Intermediate<br><br>इंटरमीडिएट<br><br>ઇન્ટરમીડિયેટ	
		14	12th Pass<br><br>12वीं पास<br><br>12મું પાસ	
		15	Matric or 10th Pass<br><br>मैट्रिक या 10वीं पास<br><br>મેટ્રિક અથવા 10મું પાસ	
		16	Under Matric or upto 10th pass<br><br>मैट्रिक से कम या 9 वीं तक पास<br><br>મેટ્રિક હેઠળ અથવા 10 પાસ સુધી	
		-77	Others.<br><br>अन्य<br><br>અન્ય	
5.6	If others, please specify<br><br>यदि अन्य, तो कृपया स्पष्ट करें			Note that this will only open if you have clicked on 'Others' in the previous question.

	જો અન્ય, તો કૃપા કરીને સ્પષ્ટ કરો			ध्यान दें कि यह तभी खुलेगा जब आपने पिछले प्रश्न में 'अन्य' पर क्लिक किया हो।<br><br>Please ask the respondent to specify that other course he/she has done.<br><br>कृपया प्रतिवादी से यह निर्दिष्ट करने के लिए कहें कि उसने अन्य पाठ्यक्रम किया है।
5.7	Age (Enter in years)<br><br>आयु (वर्षों में लिखें)<br><br>आयु (वर्षों में लिखें)			Kindly ask the respondent how old they are and note down their age in years.<br><br>कृपया प्रतिवादी से पूछें कि वे कितने वर्ष के हैं और उनकी आयु वर्षों में नोट करें।<br><br>In case the respondent does not remember their exact age, ask them the year he/she was born.<br><br>यदि प्रतिवादी को उनकी सही उम्र याद नहीं है, तो उनसे पूछें कि उनका जन्म किस वर्ष हुआ था।
5.8	Gender<br><br>लिंग<br><br>લિંગ	1	Male<br><br>पुरुष<br><br>પુરુષ	You will not ask this to the respondent.<br><br>Observe and take note of it.<br><br>आप प्रतिवादी से यह नहीं पूछेंगे।<br><br>इसे देखें और नोट करें।
		2	Female<br><br>महिला<br><br>स्त्री	
		3	Other<br><br>अन्य<br><br>અન્ય	
5.8a	Do you use a smartphone?<br><br>क्या आप स्मार्ट फोन का इस्तेमाल करते हैं?	1	Yes<br><br>हाँ<br><br>હા	Ask the respondent if he/she uses a smartphone.<br><br>प्रतिवादी से पूछें कि क्या वह स्मार्टफोन का उपयोग करता है।

	શું તમે સ્માર્ટફોનનો ઉપયોગ કરો છો?	2	No<br>नहीं<br>ના	Note that you will take their response only in ‘Yes’ , ‘No’ or ‘Occasionally’<br><br>ध्यान दें कि आप उनकी प्रतिक्रिया केवल 'हां', 'नहीं' या 'कभी-कभी' में लेंगे।
		3	Occasionally<br>कभी कभी<br>ક્યારેક	
5.8b	Are you comfortable using a smartphone for messaging or chatting with other people?<br><br>क्या आप अन्य लोगों के साथ संदेश भेजने या चैट करने के लिए स्मार्टफोन का उपयोग करने में सहज हैं?<br><br>શું તમે અન્ય લોકો સાથે મેસેજિંગ અથવા ચેટિંગ માટે સ્માર્ટફોનનો ઉપયોગ કરવા માટે આરામદાયક છો?	1	Yes<br>हाँ<br>હા	Ask the respondent to what extent he/she finds it convenient to use a smartphone for messaging other people.<br><br>प्रतिवादी से पूछें कि अन्य लोगों को संदेश भेजने के लिए स्मार्टफोन का उपयोग करना उसे किस हद तक सुविधाजनक लगता है।<br><br>**Somewhat-** You will only mark this option if the respondent says he/she is only comfortable to an extent using a smartphone for messaging or chatting with other people<br><br>कुछ हद तक- आप इस विकल्प को केवल तभी चिह्नित करेंगे जब प्रतिवादी कहता है कि वह केवल एक हद तक स्मार्टफोन का उपयोग संदेश भेजने या अन्य लोगों के साथ चैट करने के लिए सहज है<br><br>Rest of the options are simple. Mark accordingly please.<br><br>बाकी विकल्प सरल हैं। कृपया तदनुसार चिह्नित करें।
		2	No<br>नहीं<br>ના	
		3	Somewhat<br>कभी कभी<br>થોડું બોવ	
5.9	Are you comfortable taking pictures using a smartphone?<br><br>क्या आप स्मार्टफोन का उपयोग करके तस्वीरें लेने में सहज हैं	1	Yes<br>हाँ<br>હા	Please ask the respondent if they are comfortable using a smartphone for taking pictures?<br><br>कृपया प्रतिवादी से पूछें कि क्या वे चित्र लेने के लिए स्मार्टफोन का उपयोग करने में सहज हैं?
		2	No<br>नहीं	

	શું તમને સ્માર્ટ ફોનથી પિકચર લેતા ફાવે/આરામદાયક છે?		ના	
5.9a	Please indicate your educational qualification<br><br>कृपया अपनी शैक्षणिक योग्यता बताएँ<br><br>કૃપા કરીને તમારી શૈક્ષણિક લાયકાત જણાવો	1	10th Standard<br><br>10वीं कक्षा<br><br>10મું	In this question , we want to know the respondent's qualification.<br><br>इस प्रश्न में, हम प्रतिवादी की योग्यता जानना चाहते हैं।<br><br>Please ask the respondent his/her last degree that has been completed.<br><br>कृपया प्रतिवादी से पूछें कि उसकी अंतिम डिग्री जो पूरी हो चुकी है।<br><br>BHMS means Bachelor in Homeopathic Medicine and Surgery,and **BAMS** means Bachelor of Ayurveda, Medicine and Surgery.<br><br>BHMS का अर्थ है बैचलर इन होम्योपैथिक मेडिसिन एंड सर्जरी, और BAMS का अर्थ है बैचलर ऑफ आयुर्वेद, मेडिसिन एंड सर्जरी।<br><br>Rest of the options are simple and easy to understand.<br><br>बाकी विकल्प सरल और समझने में आसान हैं।<br><br>**Others-** You will only mark this option if the respondent has done some other course which is not listed in the options above.<br><br>अन्य- आप इस विकल्प को केवल तभी चिह्नित करेंगे जब प्रतिवादी ने कोई अन्य पाठ्यक्रम किया हो जो ऊपर दिए गए विकल्पों में सूचीबद्ध नहीं है।
		2	12th standard<br><br>12वीं कक्षा<br><br>12મું	
		3	Diploma<br><br>डिप्लोमा<br><br>ડિપ્લોમા	
		4	Bachelor's Degree<br><br>स्नातक डिग्री<br><br>સ્નાતકની ડિગ્રી	
		5	BHMS<br><br>बीएचएमएस<br><br>બીએચએમએસ	
		6	BAMS<br><br>बीएएमएस<br><br>બીએએમએસ	
		-77	Others<br><br>अन्य<br><br>અન્ય	
5.10	If others, please specify			Please note that this box will open if you click on 'Others' in the previous

	यदि अन्य, तो कृपया स्पष्ट करें<br><br>જો અન્ય, તો કૃપા કરીને સ્પષ્ટ કરો			question.<br><br>कृपया ध्यान दें कि यदि आप पिछले प्रश्न में 'अन्य' पर क्लिक करते हैं तो यह बॉक्स खुल जाएगा।<br><br>Ask the respondent to specify that 'Other' course that they have done which is not listed in the options here.<br><br>प्रतिवादी से उस 'अन्य' पाठ्यक्रम को निर्दिष्ट करने के लिए कहें जो उन्होंने किया है जो यहां विकल्पों में सूचीबद्ध नहीं है।
5.10 a	Which of the following best describes your work experience before you started practicing medicine?<br><br>औषधि क्षेत्र में काम शुरू करने से पहले निम्नलिखित में से कौन -सा कथन आपके कार्य अनुभव का सबसे अच्छा वर्णन करता है?<br><br>તમે ક્લિનિકલ પ્રેક્ટિસ શરૂ કરી તે પહેલાં નીચેનામાંથી કયું તમારા કામના અનુભવનું શ્રેષ્ઠ વર્ણન કરે છે?	1	Compounder with a qualified physician<br><br>योग्य चिकित्सक के साथ कंपाउंडर<br><br>એક લાયક ચિકિત્સક હેઠળ કમ્પાઉન્ડર	In this question we want to know the respondent's profession before he/she started practicing medicine.<br><br>Please ask the respondent what their profession was before practicing medicine.<br><br>Refer to the following explanation to understand the options:<br><br>1. **Compounder with a qualified physician-** Please understand that a medical compounder is more like an assistant to a doctor with practical knowledge of medicines. They are health professionals who do not have the power to prescribe medicines but instead, they can check prescriptions before prescribing medications like a pharmacist.<br>Ask the respondent if he/she was employed as a compounder with a doctor at some clinic before practising full-time medicine. Mark this if Yes<br><br>2. **Lab technician-** Please understand that a lab technician is a scientific and technical worker who assists scientists in laboratories.<br>Ask the respondent if he/she was a lab technician before this.
		2	Lab technician<br><br>लैब तकनीशियन<br><br>લેબ ટેકનિશિયન	
		3	Pharmacist<br><br>फार्मासिस्ट<br><br>ફાર્માસિસ્ટ	
		4	Paramedic technician<br><br>पैरामेडिक तकनीशियन<br><br>પેરામેડિક ટેકનિશિયન	
		-77	Others<br><br>अन्य	

			અન્ય	3. **Pharmacist-** A pharmacist is a a person who is professionally qualified to prepare and dispense medicines. Ask the respondent if he/she was a pharmacist before practising medicine.<br>4. **Paramedic technician-** Please understand that a paramedic technician is a member of the emergency services who is trained to provide basic emergency medical care before a patient is taken to a hospital. Ask the respondent if he/she was a paramedic technician before practising medicine.<br><br>5. **Others -** You will mark this option if the respondent says he/she was involved in some other profession before practising medicine.<br><br>6. **Not Applicable-** You will have to mark this option if the respondent refuses to share anything or doesn't have anything to say.
		-88	Not Applicable<br><br>लागू नहीं<br><br>લાગુ પડતું નથી	
5.11	If others, please specify<br><br>यदि अन्य, तो कृपया स्पष्ट करें<br><br>જો અન્ય, તો કૃપા કરીને સ્પષ્ટ કરો			Please note that this box will only open if the respondent says he/she was involved in some other profession which is not listed in the options here.<br><br>कृपया ध्यान दें कि यह बॉक्स तभी खुलेगा जब प्रतिवादी कहता है कि वह किसी अन्य पेशे में शामिल था जो यहां विकल्पों में सूचीबद्ध नहीं है।<br><br>Ask the respondent to specify that 'other' profession he/she was engaged in before they started practising medicine.<br><br>प्रतिवादी से यह निर्दिष्ट करने के लिए कहें कि चिकित्सा का अभ्यास शुरू करने से पहले वह 'अन्य' पेशे में लगा हुआ था।
5.12	How long have you worked in the healthcare industry?		_____Years<br>___Months	In this question , you want to know for how long has the respondent worked

	आपने स्वास्थ्य सेवा उद्योग में कितने समय तक काम किया है?<br><br>તમે આરોગ્ય ક્ષેત્રમાં કેટલો સમય કામ કર્યું છે?		___ वर्ष<br>___ माह<br><br>___વર્ષ<br>___મહિનો	in the healthcare industry.<br><br>इस प्रश्न में, आप जानना चाहते हैं कि प्रतिवादी ने स्वास्थ्य सेवा उद्योग में कितने समय तक कार्य किया है।<br><br>Instances where the respondent does not clearly remember the number of years accurately, please ask them which year they have started working in this industry.<br><br>ऐसे मामले जहां प्रतिवादी को स्पष्ट रूप से वर्षों की संख्या स्पष्ट रूप से याद नहीं है, कृपया उनसे पूछें कि उन्होंने किस वर्ष इस उद्योग में काम करना शुरू किया है।<br><br>You will note their response in the number of years and months.<br><br>आप उनकी प्रतिक्रिया को वर्षों और महीनों की संख्या में नोट करेंगे।
5.13	How long have you been working as an independent health care provider? (Please enter in years.)<br><br>आप एक स्वतंत्र स्वास्थ्य सुविधा प्रदाता के रूप में कितने समय से काम कर रहे हैं?<br><br>(कृपया वर्षों में लिखें)<br><br>તમે કેટલા સમયથી સ્વતંત્ર આરોગ્ય સંભાળ પ્રદાતા તરીકે કામ કરો છો?<br><br>(કૃપા કરીને વર્ષમાં લખો.)		_____Years<br>___Months<br><br>___ वर्ष<br>___ माह<br><br>___વર્ષ<br>___મહિનો	Please understand that an **independent healthcare provider** is a person who provides healthcare services independently that is neither owned or managed by any central or state government.<br><br>कृपया समझें कि एक स्वतंत्र स्वास्थ्य सेवा प्रदाता एक ऐसा व्यक्ति है जो स्वतंत्र रूप से स्वास्थ्य सेवाएं प्रदान करता है जो न तो किसी केंद्र या राज्य सरकार के स्वामित्व या प्रबंधन के अधीन है।<br><br>Ask the respondent for how long he/she has been practising as an independent healthcare provider.<br><br>प्रतिवादी से पूछें कि वह एक स्वतंत्र स्वास्थ्य सेवा प्रदाता के रूप में कितने समय से अभ्यास कर रहा है।<br><br>Note their response in years and months.

				वर्षों और महीनों में उनकी प्रतिक्रिया नोट करें।
5.14	How many patients do you see per **day** on average?<br><br>आप प्रतिदिन औसतन कितने रोगी देखते हैं?<br><br>દરરોજના સરેરાશ કેટલા દર્દીઓ જુઓ છો?	1	1-10<br><br>1-१०	In this question , you need to ask the respondent how many patients they attend in a day on an average basis.<br><br>इस प्रश्न में, आपको प्रतिवादी से यह पूछने की आवश्यकता है कि वे औसतन एक दिन में कितने रोगियों को देखते हैं।<br><br>Options are simple and straightforward. Mark accordingly.<br><br>विकल्प सरल और सीधे हैं। तदनुसार चिह्नित करें।
		2	11-50<br><br>११-५०	
		3	51-100<br><br>५१-१००	
		4	101-200<br><br>१०१-२००	
		5	More than 200<br><br>200 से अधिक<br><br>૨૦૦થી વધુ	
5.14a	On average, how much do you charge per patient visit? (In Rupees)<br><br>आप औसतनप्रति मरीज को देखने पर कितना शुल्क लेते हैं ? (रुपये में)<br><br>તમે દર્દીની મુલાકાત દીઠ સરેરાશ કેટલો ચાર્જ કરો છો? (રૂપિયામાં)	1	Free of cost<br><br>निःशुल्क<br><br>મફત માં	This question focuses on understanding how much money is charged by the respondent from every patient that he/she sees.<br><br>यह प्रश्न यह समझने पर केंद्रित है कि प्रतिवादी द्वारा प्रत्येक रोगी से कितना पैसा वसूला जाता है, जिसे वह देखता है।<br><br>Options are simple. Mark as they respond.<br><br>विकल्प सरल हैं। उनके जवाब के रूप में चिह्नित करें।<br><br>If others, please specify- Please mark this option if the respondent mentions some other price which is not listed in the options here.<br><br>यदि अन्य हैं, तो कृपया निर्दिष्ट करें- कृपया इस विकल्प को चिह्नित करें यदि प्रतिवादी
		2	1-99<br><br>૦-૯૯	
		3	100-149<br><br>૧૦૦ -૧૪૯	
		4	150-199<br><br>૧૫૦-૧૯૯	
		5	200-249<br><br>૨૦૦-૨૪૯	
		6	<250	

			<२५०	किसी अन्य मूल्य का उल्लेख करता है जो यहां विकल्पों में सूचीबद्ध नहीं है। .
		-77	If others, please specify<br><br>यदि अन्य तो कृपया स्पष्ट करें<br><br>જો અન્ય, તો કૃપા કરીને સ્પષ્ટ કરો	
5.15	If others, please specify<br><br>यदि अन्य, तो कृपया स्पष्ट करें<br><br>જો અન્ય, તો કૃપા કરીને સ્પષ્ટ કરો			Note that this box will only open if you have clicked on ‘Others’ in the previous question.<br><br>ध्यान दें कि यह बॉक्स तभी खुलेगा जब आपने पिछले प्रश्न में 'अन्य' पर क्लिक किया हो।<br><br>Ask the respondent what is that ‘Other’ amount which he/she charges from patients?<br><br>प्रतिवादी से पूछें कि वह 'अन्य' राशि क्या है जो वह रोगियों से वसूल करता है?
