# Supplementary material for: Understanding Providers’ Attitude Toward AI in India’s Informal Health Care Sector: Survey Study
Source: JMIR Form Res. 2025 Feb 10;9:e54156. doi: 10.2196/54156 (PMC11832356; doi:10.2196/54156)
Supplement: Multimedia Appendix 3 [file formative-v9-e54156-s003.pdf]

Appendix 3: Outlier analysis

A) Data cleaning using boxplot analysis.

The analysis utilized the quantitative variable "Age," which had a wide range. We employed a boxplot analysis to detect outliers within this variable and proceeded to eliminate data points that exceeded 1.5 times the Interquartile Range (IQR). In the process, two observations, one from Gujarat and one from Jharkhand, were excluded from the dataset. Consequently, the final dataset contained information from 312 observations, comprising 226 from Gujarat and 86 from Jharkhand.

B) Data cleaning for high-influence outliers.

To ensure the robustness of the analysis, we eliminated a total of 24 outliers with high influence using Cook's distance analysis for the linear regression (OLS). This process resulted in a final dataset consisting of 288 observations, with 211 observations from Gujarat and 77 observations from Jharkhand.

Cook's distance estimates how much a regression model changes by removing the ith datapoint from the model.

$$D = \frac{\sum_{j=1}^{n}\left(y_j - y_{j(i)}\right)^2}{(P+1)\sigma^2}$$

P = number of regression coefficients

σ^2 measures mean squared error.

y_j is j^{th} fitted response value,

$y_{j(i)}$ is j^{th} fitted response value where fit doesn't include i^{th} observation.

Threshold Cook's distance D

$D < 4/(N-k-1)$

N= Observations

k = number of explanatory variables

For our analysis k = 4

For Jharkhand N = 86, D<0.05............. (1)

For Gujarat N = 226, D< 0.018............... (2)

Selecting datapoints fulfilling (1) and (2)

The final dataset contains a total of 288 observations, 211 from Gujarat and 77 from Jharkhand.
