# Supplementary material for: Understanding Providers’ Attitude Toward AI in India’s Informal Health Care Sector: Survey Study
Source: JMIR Form Res. 2025 Feb 10;9:e54156. doi: 10.2196/54156 (PMC11832356; doi:10.2196/54156)
Supplement: Multimedia Appendix 4 [file formative-v9-e54156-s004.pdf]

28th January, 2022

Sarang Deo,
Associate Professor,
Executive Director, Max Institute of Healthcare Management,
Operations Management, Indian School of Business,
Hyderabad, India

Dear Sarang Deo,

The Indian School of Business-Institutional Review Board (ISB-IRB) is pleased to inform you that your IRB application with the protocol number "**ISB-IRB 2022-04**" and with the title **"The impact of using AI based Solution on TB Diagnosis Care Cascade in India"** is granted IRB Conditional Approval.

Condition to obtain Full IRB Approval: Please submit CITI certificate of the supervisor of data collector.

Good Luck with your study!

Note:

1. Please note that the **IRB should be notified in case of any Reportable new information** **Reportable new information:** Work done outside of the approved protocol, participant problems and adverse events.
2. **The IRB should be notified before a change is made to the current study.**

Things that require IRB review and modification approval

1. Change in study design
2. Change in funding
3. Change in investigators
4. Change in approved questionnaires
5. Changes to the subject recruitment material that was approved

For questions about the need to submit modification, please contact: IRBassist@isb.edu

Regards,

Ashwini Chhatre,
Chair, Institutional Review Board,
Indian School of Business,
Hyderabad Campus, Gachibowli,
Hyderabad, India

Application for Review by Institutional Review Board - Addendum, Indian School of Business, Hyderabad

Principal Investigator (PI)

First Name :	Sarang	Last Name :	Deo
Department :	Operations		
E-mail address :	sarang_deo@isb.edu	Phone :	9640233211

Status (check one)

ISB Faculty ✓	ISB Student	Other ISB	Non ISB

Faculty Sponsor(*Required for all students or non-ISB investigators*)

First Name :	Last Name :
Department :	
E-mail address :	Phone :

Co-Investigators/Researchers

First Name :	Raghuram	Last Name :	Bommaraju
Department :	Marketing	Phone: 9779383652	

Co-Investigators/Researchers

First Name :	Sumeet	Last Name :	Kumar
Department :	Information Systems	Phone : 7075969318	

Co-Investigators/Researchers (You can add additional Investigators/Researchers)

First Name :	Sirisha	Last Name :	Papineni
Department :	World Health Partners	Phone :	+1 630 301 4093

Co- Investigators /Researchers at School's other than ISB should provide IRB approval details at their School and IRB Protocol Number. The ISB- IRB Committee would officially communicate with your School's IRB.

Project Title :

The Impact of Using an AI based Solution on TB Diagnosis Care Cascade in India

Investigator's Agreement

I agree to use procedures with respect to safeguarding human subjects in this activity that conform to state and ISB policy. If there is a significant change in the research procedure, I shall seek prior approval from IRB. If this is a continuation or renewal of ongoing program, I affirm that the procedure followed conform to this policy.

Principle Investigator Signature		Date	
Faculty Sponsor Signature		Date	

Will the project involve any of the following? (*Check all that apply*)

No	Deception (research in which subjects are purposely let to have false beliefs or assumptions)
No	More than minimal risks to subjects (i.e., risk greater than that of everyday life)
No	Investigational new drug or device exemption

Is the application for a pilot study?

	Yes
✓	No

**If yes, what is the name of funding agency, organization or ISB program?**
Not Applicable

What is the status of the request for funding?

	Submission planned in near future
✓	Submitted and pending
	Initial award
	Continuation award

	Renewal award
If already awarded what is the award or grant no?	Not Applicable

What is the title of the project as it appears in the submission for funding or award?

Effectiveness of AI for Clinical Diagnosis in Low- and Middle-Income Countries: Case of TB Diagnosis in India

1. **What are the updates to the study plan?**

In the IRB application, we proposed to survey radiologists to understand the effect of moderating factors influencing the main research hypotheses: 1) "The introduction of an AI software will increase the TB case findings", 2) "The introduction of an AI software will reduce the diagnostic delay". In this addendum, we would like to extend the survey to Informal Providers (IPs) to understand their attitude towards AI, and how that impacts the research hypotheses.

In India, IPs (who do not have a formal medical degree but provide care) are the first point of contact for many patients in rural and suburban areas. Though IPs commonly use X-Rays by IPs to screen patients for TB, they are, in general, trained to interpret an X-Ray (with or without a report) or combine the interpretation with clinical examination to make a confirmed TB diagnosis.

In the main IRB application, we proposed to study the uptake of AI software by radiologists, and how that depends on the attitude of radiologists towards AI, by conducting a survey to ascertain the perspective of radiologists. The research hypothesis examined by the radiologist survey is "Attitude, experience and skills of radiologists will moderate the use of AI for clinical decision making."Though radiologists use AI software, they do not diagnose TB cases. The X-Ray reports are handed over to IPs via patients who could study the report for diagnosing the patients. As IPs are the ones reading the X-Ray reports, any changes to the X-Ray reports (vis-à-vis radiologists using AI software) are also likely to influence IPs decision. Therefore, in addition to radiologists, the main research hypotheses also depend on the interpretation of the X-Ray report by the IPs.

Hence, we propose to conduct an IPs survey to collect data that will help us to test the following hypotheses: 1) "The effect of AI will be stronger for IPs that did not have a radiologist report available" (e.g., because radiologists are not available at X-Ray labs), 2) "The effect of AI will be weaker for IPs with higher qualification or more experience", and 3) "The effect of AI will be stronger for IPs with more trust in AI".

2. **Subject selection and recruitment**
   As this is a joint project with WHP (as described in the main IRB application), we use the set of IPs who are already engaged with WHP. 305 IPsJharkhand.

3. **Statements to subjects**

   Consent form is provided as an attachment.

4. **Procedures to be followed**
   The survey questionnaire (attached) is developed by faculty members at ISB. The questionnaire will be administered by staff at a contracted research agency (under discussion). PI and co-PIs of this project will monitor and supervise the administration of the survey and ensure that it is in compliance with the IRB guidelines.

5. **Attachments**

Please attach your Questionnaire. (Required if study has an instrument):
A complete Questionnaire, <u>as seen by the subjects</u> is required.

IPs survey for the main-study is attached.

Note: A Pre-test Questionnaire / Main- study Questionnaire should be attached separately and IRB approval should be sought for both the studies.

<u>FOR OFICE USE ONLY</u>

Approve
Reject
Pending

APPROVAL SOURCE

By e-mail
By meeting
By both

SUGGESTIONS/FEEDBACK

Application for Review by Institutional Review Board, Indian School of Business, Hyderabad

Principal Investigator (PI)

First Name : Sarang   Last Name : Deo
Department : Operations
E-mail address : sarang_deo@isb.edu   Phone : 9640233211

Status (check one)

ISB Faculty ✓   ISB Student   Other ISB   Non ISB

Faculty Sponsor(*Required for all students or non-ISB investigators*)

First Name :   Last Name :
Department :
E-mail address :   Phone :

Co-Investigators/Researchers

First Name : Raghuram   Last Name : Bommaraju
Department : Marketing   Phone: 9779383652

Co-Investigators/Researchers

First Name : Sumeet   Last Name : Kumar
Department : Information Systems   Phone : 7075969318

Co-Investigators/Researchers (You can add additional Investigators/Researchers)

First Name : Sirisha   Last Name : Papineni

Department : World Health Partners   Phone : +1 630 301 4093

Co- Investigators /Researchers at School's other than ISB should provide IRB approval details at their School and IRB Protocol Number. The ISB- IRB Committee would officially communicate with your School's IRB.

Project Title :

The Impact of Using an AI based Solution on TB Diagnosis Care Cascade in India

Investigator's Agreement

I agree to use procedures with respect to safeguarding human subjects in this activity that conform to state and ISB policy. If there is a significant change in the research procedure, I shall seek prior approval from IRB. If this is a continuation or renewal of ongoing program, I affirm that the procedure followed conform to this policy.

Principle Investigator Signature	Sarang Deo	Date	December 24th, 2021, 2021
Faculty Sponsor Signature		Date	

Will the project involve any of the following? (*Check all that apply*)

No	Deception (research in which subjects are purposely let to have false beliefs or assumptions)
No	More than minimal risks to subjects (i.e., risk greater than that of everyday life)
No	Investigational new drug or device exemption

Is the application for a pilot study?

	Yes
✓	No

**If yes, what is the name of funding agency, organization or ISB program?**
Not Applicable

What is the status of the request for funding?

	Submission planned in near future
✓	Submitted and pending
	Initial award

	Continuation award
	Renewal award
If already awarded what is the award or grant no?	Not Applicable

What is the title of the project as it appears in the submission for funding or award?

Effectiveness of AI for Clinical Diagnosis in Low- and Middle-Income Countries: Case of TB Diagnosis in India

PURPOSE OF STUDY

Please describe the purpose of your proposed research, making clear the research question. Explanation must be clear to those unfamiliar with your field. References are unnecessary.

Tuberculosis (TB) is one of India's biggest health concerns causing over 200,000 deaths per year. In this study, we plan to estimate the impact of using an AI solution on TB diagnosis care cascade in India. This study is a part of a project undertaken by World Heath Partners (WHP) to evaluate the benefits of using X-rays for TB diagnosis.

One aspect of the WHP project involves exploring the usage of AI software to generate X-Ray reports. For this, WHP plans to share AI software with X-Ray labs to understand the effect of AI-generated reports in diagnosing TB cases. Their research hypotheses are 1) "The introduction of an AI software will increase the TB case findings", 2) "The introduction of an AI software will reduce the diagnostic delay".

In this study, we support WHP on their implementation strategy on how the intervention (of allowing X-Ray labs to use an AI software) should be staggered to derive conclusive insights about the benefits of using the AI software. As the uptake of AI software also depends on the attitude of software users' (radiologists) towards AI, we will design and conduct a survey to ascertain the perspective of radiologists. The research hypothesis examined by the survey is "Attitude, experience and skills of radiologists will moderate the use of AI for clinical decision making."

Background

In India, informal providers (who do not have a formal medical degree but provide care) are the first point of contact for many patients in rural and suburban areas. Though X-Rays are commonly used tool to screen patients for TB, the informal providers (IPs) are not, in general, trained to interpret an X-Ray (with or without a report) and combine the interpretation with clinical examination to diagnose a TB case. Moreover, many of the X-Ray laboratories (labs) in India do not have a resident radiologist, which means that the X-ray

image may not be accompanied by a report, making the interpretation and diagnosis even harder for the IPs.

To address these issues, WHP (World Health Partners) is testing the use of an AI application to generate X-Ray reports (which we call AI report, henceforth). There are two use cases of these AI generated reports. In the first use case, when a radiologist is available at the X-Ray lab, he/she will see the AI report and then may use the AI recommendations to write (or update) his/her own interpretation of the X-Ray report. In this case, the report written by the radiologist will be given to the patient by the X-Ray lab and will be subsequently taken to the consulting IP. In the second use case, when radiologist is not available at an X-Ray lab, the AI report will be printed and given to the patient, clearly highlighting that the report has been generated using a software.

For the above two use cases, WHP plans to examine the hypothesis “AI will have a positive effect on the number of TB cases suggested/identified by the Radiologists”. The primary mechanism to test the hypothesis is by providing free AI software at the participating X-Ray labs. The AI software is developed by DeepTek Medical Imaging Private Limited[1], which worked with World Health Partner to provide an integrated web platform for the X-Ray labs to use the AI software (see Fig. 1).

To understand the moderating factors influencing the hypothesis outcome, we will conduct a radiologist survey that would study the following three hypotheses:
1) “The effect of AI on case findings will be weaker for Radiologists with more experience (age or qualifcation)”,
2) “The effect of AI on case findings will be weaker for Radiologists with higher self-efficacy”, and
3) “The effect of AI on case findings will be stronger for Radiologists with higher trust in AI or past experience with AI”.

[1] https://www.deeptek.ai

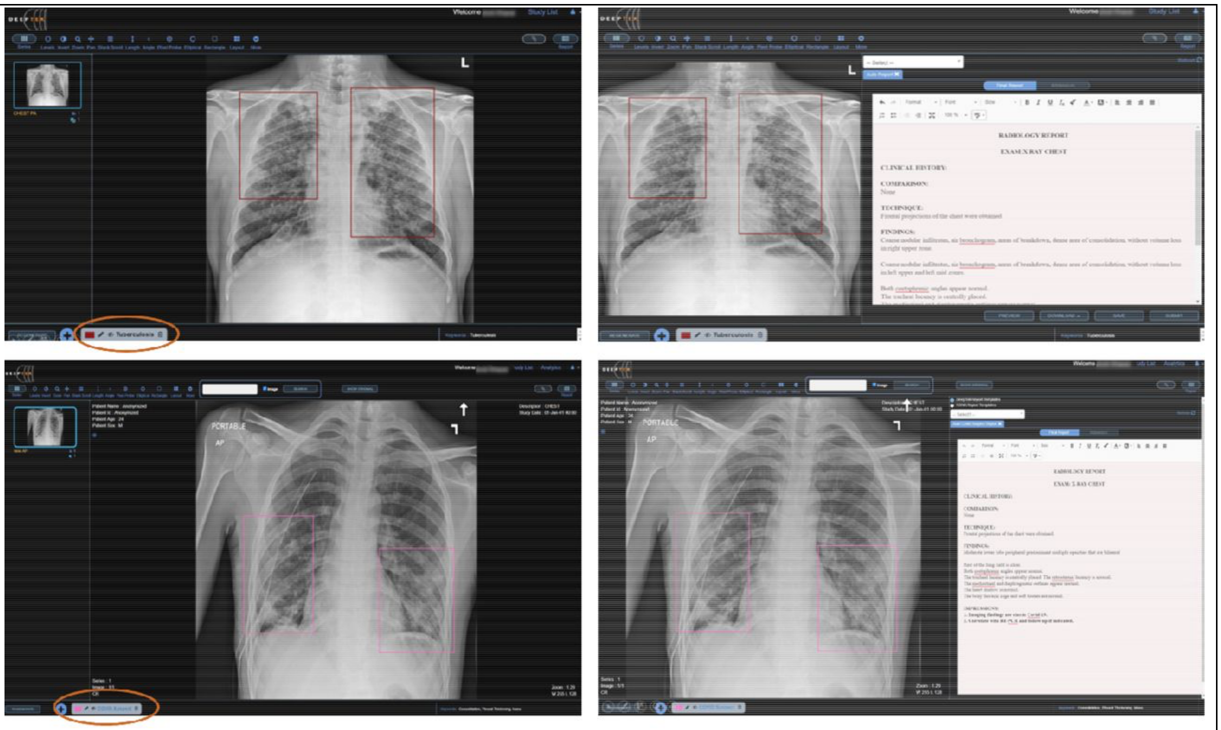

Figure 1. DeepTek User Interface showing X-Ray scans and Radiology reports
(Image source: https://www.medrxiv.org/content/10.1101/2020.10.13.20178483v1.full)

Summary:
WHP is evaluating the effect of AI on TB case findings. In this study, we will support WHP: 1) by suggesting a staggered approach of providing the AI software to X-ray labs, 2) by surveying the radiologists to understand the effect of different attitudes (trust, experience, and self-efficacy) towards AI on the uptake of the AI software, and 3) by analysing deidentified patient data shared by WHP and the data obtained via the survey of radiologists (which we will conduct), to evaluate the research hypotheses.

1. SUBJECT SELECTION AND RECRUITMENT

Describe in detail how the subject will be recruited

Selection of subjects must be equitable, scientifically justifiable and avoid any potential for coercion

What is the expected size (number of subjects to be included)?

There are two kinds of participants in our study: 1) X-Ray labs, and 2) Informal Providers (IPs)

To understand the impact of using X-Rays for TB diagnosis in India, WHP has initiated a multi-year project. For the project, WHP has recruited X-Ray labs and IPs. For our study, in which we try to understand the effect of using AI software on TB diagnosis, we plan to use the same set of X-Ray labs that are working with WHP on their project, so in that sense the selection of subjects is beyond our consideration.

Our subjects are radiologists at X-Ray labs which are also the primary intervention target. As mentioned earlier, our partner in the study, the World Health Partner (WHP), through their staff who visit X-Ray labs, has identified 186 X-Ray labs. These X-Ray labs that have agreed to participate in the project are from Gujrat and Jharkhand. We plan to survey the radiologists in these X-Ray labs for our study. Just like X-Ray labs, WHP staff members also meet IPs and enroll those IPs who are interested in their project.

The current number of X-Ray labs working with WHP is around 186, and the number of IPs is 1163. Specifically, in Gujarat, WHP is engaged with 73 X-Ray labs and 757 IPs. In Jharkhand, WHP is currently working with 113 X-Ray labs and 406 IPs.

3. **STATEMENT TO SUBJECTS**

The statement to the subject should include information on the purpose of the study, what subjects will be asked to do if they participate, where the study will be carried out, how much of the subjects' time participation may take, and what type of information they might be asked to provide. It should also make clear that participation is voluntary and that subjects may withdraw from the study at anytime.
Include a copy of any written statements or verbal scripts to be used. We recommend a consent form along the following:

Consent form is provided as another attachment.

4. HARM OR BENEFIT TO SUBJECTS

Describe any potential harm/risk or benefit to the subjects. If there is no more than minimal risk to subjects (e.g., no greater than of everyday life), then state that "there is no risk from participation beyond that of everyday life". If there is the possibility of greater than minimal risk, please include details. ***Note:*** *Gifts or payment of any kind to participants are incentives or reimbursements not benefits. If any gifts or incentives will be offered to subjects, provide details in the section on procedures. Information on incentives should be made clear in recruitment materials and consent/permission forms.*

There is no risk from participation beyond that of everyday life. Because we provide an AI software for generating X-Ray reports, radiologists and IPs will get "additional" information as an AI report, compared to baseline. Both radiologists and IPs can decide when to ignore the AI reports and fall back to baseline behavior. Because the quality of DeepTek AI algorithm (used for generating AI reports) has been studied in past and found to achieve expert level performance in diagnosing TB chest radiographs[2,3], we expect the additional information will not make the situation worse for patients.

5. PROCEDURES TO BE FOLLOWED

Describe the procedures to be followed in carrying out the project, including where the study will be conducted; what the participants will be expected to do at each stage of the project; who will supervise the participation or conduct interviews; and any incentives or reimbursements for participants

Where the study will be conducted:
WHP has identified two states as the target of their project on the use of X-Rays to improve TB diagnosis. The project implementation is planned in 1) Gujarat, 2) Jharkhand. In each of the two states, some X-Ray labs and Informal Providers have agreed to participate in their project. As mentioned earlier, in Gujarat, WHP is engaged with 73 X-Ray labs and 757 IPs. In Jharkhand, WHP is currently working with 113 X-Ray labs and 406 IPs.

What the participants are expected to do at each stage:
WHP plans to intervene at the level of X-Ray labs. At X-Ray labs with radiologists, radiologists will have access to an integrated web platform (described earlier). X-ray labs that have no radiologists, there are people handling X-Ray imaging for customers. In absence of a precise title, let's call them X-ray lab supervisors. Radiologists and X-Ray supervisors will get access to the integrated WHP platform. The web platform will allow logging in to the system to upload X-Ray images and X-Ray reports. In addition, the web platform will also allow to view the AI-based reports, make any changes to the AI-based report, and print the AI-based reports. X-Ray lab supervisors/radiologists are responsible for modifying and printing the AI reports and handing them to patients. Informal Providers (IPs) use X-Ray images and X-Ray reports that suspected TB patients bring to them for diagnosis.

As a part of this study, we will also conduct a survey to understand radiologist's' attitude towards AI and technology. The survey questions are added as an attachment.

Who will supervise the participation or conduct interviews:

[2] https://europepmc.org/article/ppr/ppr225470
[3] https://www.nature.com/articles/s41598-021-03265-0

WHP team is working with the DeepTek team to adapt the design of the AI solution to ensure a smooth user-experience for X-Ray lab supervisors and radiologists. The WHP team is also responsible for training the staff at the X-Ray labs to work with the software effectively.

The investigators at ISB are responsible for creating a survey to understand the attitude of radiologists toward the use of AI for clinical decision making. The survey questionnaire (attached) is developed by faculty members at ISB. The questionnaire will be used by staff at a contracted research agency (to be finalized), who will visit the radiologists and the IPs to get survey responses. The whole process of designing and conducting surveys will be supervised by PI and co-PIs.

Any incentives or reimbursements for participants:

There is no incentive for radiologists to participate in the survey. While recruiting X-Ray labs, WHP agreed to reimburse the X-Ray labs the cost of providing free X-Rays to patients who are referred by the participating IPs. For our study on the use of AI to diagnose TB, we use the same set of X-Ray labs that are already engaged with WHP. We are not providing any additional incentives to the X-Ray labs.

What is done with the data that gets collected?

As IPs register the suspected TB patients on the WHP platform, we would get access to patients' data via WHP in a deidentified form. WHP team also follows up with the patients to find the final diagnosis, so we would also get information on the final diagnosis of deidentified patients. We plan to use this information to test the research hypotheses.

The research agency that will conduct the radiologist survey will share the survey responses with us. We will again use these responses for answering the research questions.

6. CONFIDENTIALITY

Describe the specific methods by which confidentiality will be protected (i.e., use of data coding systems or pseudonyms). If the study includes more than one session or instrument, anonymity may be achieved by assigning code names that track participants' data from one session or document to another but are unrelated to participants' true names. If any online surveys or responses are included in the procedures, describe the methods to be used to ensure that identifying material will not be transmitted or recorded electronically (e.g., email address, IP numbers)

We will store project related information on a common OneDrive folder (created by Prof. Sumeet Kumar). The project folder would only be accessible by the project team members.<br><br>In case of sensitive/identifiable information, there would be subfolders with limited access to the PI and co-PIs responsible for collating the sensitive files. As and when the project ends, the access to the project folders will be removed for the team. All the identifiers from the data would be removed prior to any publication of results.<br><br>The sensitive data in the form of paper would have limited access and will be managed by the principal investigator.

7. **COOEPRATING INSTITUTIONS**

List all institutions expected to provide access to potential subjects, to data necessary to identify subjects, to data previously collected, or facilities where the research is to be conducted. A letter from an appropriate senior official, on letter head, should be obtained from organizations that do not have an IRB.

A	Indian School of Business
B	World Health Partners
C	

8. Attachments:

Please attach your Questionnaire. (Required if study has an instrument):
A complete Questionnaire, <u>as seen by the subjects</u> is required.

Note: A Pre-test Questionnaire / Main- study Questionnaire should be attached separately and IRB approval should be sought for both the studies.

<u>FOR OFICE USE ONLY</u>

Approve
Reject
Pending

APPROVAL SOURCE

By e-mail
By meeting
By both

SUGGESTIONS/FEEDBACK
