# Supplementary material for: Understanding Providers’ Attitude Toward AI in India’s Informal Health Care Sector: Survey Study
Source: JMIR Form Res. 2025 Feb 10;9:e54156. doi: 10.2196/54156 (PMC11832356; doi:10.2196/54156)
Supplement: Multimedia Appendix 5 [file formative-v9-e54156-s005.pdf]

Appendix 4 : Descriptor variables

Table S1. Descriptor variables for initial sample

	Jharkhand				Gujarat			
Variable	Mean	SD	Min	Max	Mean	SD	Min	Max
**Age(years)**	42.15	12.69	22	86	41.61	10.44	22	76
**No. of years in worked in healthcare**	15.43	10.43	1	47	16.21	9.8	1	50
**Gender, n(%)**								
Male	124(76.5)				222(91)			
Female	38(23.5)				22(9)			
**Education, n(%)**								
School education	32(19.8)				0(0)			
Diploma	27(16.7)				16(6.6)			
Bachelors	37(22.8)				219(89.8)			
Post Graduation	14(8.6)				1(0.4)			
Others	52(32.1)				8(3.3)			
Observations	162				244			

Table S2. Descriptor variables for the final sample (after data cleaning)

	Jharkhand				Gujarat			
Variables	Mean	SD	Min	Max	Mean	SD	Min	Max
**Age(years)**	40.87	10.92	24	67	40.48	9.33	22	68
**No. of years worked in healthcare**	15.34	10.13	3	47	15.26	8.77	1	42
**Gender, n(%)**								
Male	61(79.2)				194(92.4)			
Female	16(20.8)				16(7.6)			
**Education, n(%)**								
School education	15(19.5)				0(0)			
Diploma	12(15.6)				15(7.1)			
Bachelors	27(35.1)				190(90)			
Post Graduation	4(5.2)				1(0.5)			
Others	19(24.7)				5(2.4)			
Observations	77				211			
