# Supplementary material for: Understanding Providers’ Attitude Toward AI in India’s Informal Health Care Sector: Survey Study
Source: JMIR Form Res. 2025 Feb 10;9:e54156. doi: 10.2196/54156 (PMC11832356; doi:10.2196/54156)
Supplement: Checklist 1 [file formative-v9-e54156-s006.pdf]

STROBE Statement—checklist of items that should be included in reports of observational studies

	Item No.	Recommendation	Page No.	Relevant text from manuscript
**Title and abstract**	1	(*a*) Indicate the study's design with a commonly used term in the title or the abstract	1	A cross-sectional survey study as mentioned in the Methods section in the abstract.
		(*b*) Provide in the abstract an informative and balanced summary of what was done and what was found	1	Provided in Methods and Results section in abstract
**Introduction**				
Background/rationale	2	Explain the scientific background and rationale for the investigation being reported	1-2	Included in Introduction
Objectives	3	State specific objectives, including any prespecified hypotheses	1-2	Included in Introduction
**Methods**				
Study design	4	Present key elements of study design early in the paper	2	Included in the Methods section
Setting	5	Describe the setting, locations, and relevant dates, including periods of recruitment, exposure, follow-up, and data collection	2-4	Included in the Methods section.
Participants	6	(*a*) *Cohort study*—Give the eligibility criteria, and the sources and methods of selection of participants. Describe methods of follow-up<br>*Case-control study*—Give the eligibility criteria, and the sources and methods of case ascertainment and control selection. Give the rationale for the choice of cases and controls<br>*Cross-sectional study*—Give the eligibility criteria, and the sources and methods of selection of participants	2-4	Included in the "Participant's Recruitment and Enrolment" and "Data" subsections in Methods section.
		(*b*) *Cohort study*—For matched studies, give matching criteria and number of exposed and unexposed<br>*Case-control study*—For matched studies, give matching criteria and the number of controls per case		Not applicable
Variables	7	Clearly define all outcomes, exposures, predictors, potential confounders, and effect modifiers. Give diagnostic criteria, if applicable	4-5	Included in "Variable description and analysis" subsection in Methods
Data sources/ measurement	8*	For each variable of interest, give sources of data and details of methods of assessment (measurement). Describe comparability of assessment methods if there is more than one group	4-5	Included in "Variable description and analysis"

				subsection in Methods
Bias	9	Describe any efforts to address potential sources of bias	8	Addressed in Limitations section
Study size	10	Explain how the study size was arrived at	3	Included in "Data" subsection in Methods

Continued on next page

Quantitative variables	11	Explain how quantitative variables were handled in the analyses. If applicable, describe which groupings were chosen and why	2-5	Included in Methods
Statistical methods	12	(*a*) Describe all statistical methods, including those used to control for confounding	2-5	Included in Methods
		(*b*) Describe any methods used to examine subgroups and interactions	2-5	Included in Methods
		(*c*) Explain how missing data were addressed	3-4	Included in "Data" within Methods
		(*d*) *Cohort study*—If applicable, explain how loss to follow-up was addressed<br>*Case-control study*—If applicable, explain how matching of cases and controls was addressed<br>*Cross-sectional study*—If applicable, describe analytical methods taking account of sampling strategy		Not applicable
		(*e*) Describe any sensitivity analyses		Not applicable
**Results**				
Participants	13*	(a) Report numbers of individuals at each stage of study—eg numbers potentially eligible, examined for eligibility, confirmed eligible, included in the study, completing follow-up, and analysed	3-4	Included in "Data" subsection in Methods
		(b) Give reasons for non-participation at each stage	3-4	Included in "Data " subsection in Methods
		(c) Consider use of a flow diagram	4	Included in "Data", Figure1
Descriptive data	14*	(a) Give characteristics of study participants (eg demographic, clinical, social) and information on exposures and potential confounders	3-5	Included in "Data" and "Descriptive Statistics" under Results
		(b) Indicate number of participants with missing data for each variable of interest	3-4	Included in "Data" in Methods
		(c) *Cohort study*—Summarise follow-up time (eg, average and total amount)		Not applicable
Outcome data	15*	*Cohort study*—Report numbers of outcome events or summary measures over time		Not applicable
		Case-control study—Report numbers in each exposure category, or summary measures of exposure		Not applicable
		Cross-sectional study—Report numbers of outcome events or summary measures	5	Included in Table 2 in the Results
Main results	16	(*a*) Give unadjusted estimates and, if applicable, confounder-adjusted estimates and their precision (eg, 95% confidence interval). Make clear which confounders were adjusted for and why they were included	6	Included in Table 3,4 in the Results
		(*b*) Report category boundaries when continuous variables were categorized		Not applicable
		(*c*) If relevant, consider translating estimates of relative risk into absolute risk for a meaningful time period		Not applicable

Continued on next page

Other analyses	17	Report other analyses done—eg analyses of subgroups and interactions, and sensitivity analyses	4-6	Assessing internal consistency of scale included in Variable and analysis subsection of Methods section and Results section
**Discussion**				
Key results	18	Summarise key results with reference to study objectives	6-7	Included in Discussion
Limitations	19	Discuss limitations of the study, taking into account sources of potential bias or imprecision. Discuss both direction and magnitude of any potential bias	8	Included in Limitations
Interpretation	20	Give a cautious overall interpretation of results considering objectives, limitations, multiplicity of analyses, results from similar studies, and other relevant evidence	6-7	Included in Discussion
Generalisability	21	Discuss the generalisability (external validity) of the study results	6-8	Included in the Discussion and Conclusion
**Other information**				
Funding	22	Give the source of funding and the role of the funders for the present study and, if applicable, for the original study on which the present article is based	8	Included in Acknowledgement

*Give information separately for cases and controls in case-control studies and, if applicable, for exposed and unexposed groups in cohort and cross-sectional studies.

Note: An Explanation and Elaboration article discusses each checklist item and gives methodological background and published examples of transparent reporting. The STROBE checklist is best used in conjunction with this article (freely available on the Web sites of PLoS Medicine at http://www.plosmedicine.org/, Annals of Internal Medicine at http://www.annals.org/, and Epidemiology at http://www.epidem.com/). Information on the STROBE Initiative is available at www.strobe-statement.org.
